# The SARS-CoV-2 receptor ACE2 is expressed in mouse pericytes but not endothelial cells: Implications for COVID-19 vascular research

**DOI:** 10.1016/j.stemcr.2022.03.016

**Published:** 2022-04-21

**Authors:** Lars Muhl, Liqun He, Ying Sun, Maarja Andaloussi Mäe, Riikka Pietilä, Jianping Liu, Guillem Genové, Lei Zhang, Yuan Xie, Stefanos Leptidis, Giuseppe Mocci, Simon Stritt, Ahmed Osman, Andrey Anisimov, Karthik Amudhala Hemanthakumar, Markus Räsänen, Emil M. Hansson, Johan Björkegren, Michael Vanlandewijck, Klas Blomgren, Taija Mäkinen, Xiao-Rong Peng, Yizhou Hu, Patrik Ernfors, Thomas D. Arnold, Kari Alitalo, Urban Lendahl, Christer Betsholtz

**Affiliations:** 1 Department of Medicine, Huddinge, Karolinska Institutet, Solna, Sweden; 2 Department of Immunology, Genetics, and Pathology, Rudbeck Laboratory, Uppsala University, Uppsala, Sweden; 3 Department of Neurosurgery, Tianjin Medical University General Hospital, Tianjin Neurological Institute, Key Laboratory of Post-Neuro-injury Neuro-Repair and Regeneration in Central Nervous System, Ministry of Education and Tianjin City, Tianjin 300052, China; 4 Key Laboratory of Ministry of Education for Medicinal Plant Resource and Natural Pharmaceutical Chemistry, National Engineering Laboratory for Resource Developing of Endangered Chinese Crude Drugs in Northwest of China, College of Life Sciences, Shaanxi Normal University, Xi’an China; 5 Department of Women’s and Children’s Health, Karolinska Institutet, Solna, Sweden; 6 Wihuri Research Institute and Translational Cancer Medicine Program, Biomedicum Helsinki, University of Helsinki, Helsinki, Finland; 7 Institute of Genomics and Multiscale Biology, Department of Genetics and Genomic Sciences, Icahn School of Medicine at Mount Sinai, New York, NY 10029, USA; 8 Department of Pediatric Oncology, Karolinska University Hospital, Stockholm Sweden; 9 Cardiovascular, Renal and Metabolism, AstraZeneca BioPharmaceutical R&D, Gothenburg, Sweden; 10 Department of Medical Biochemistry and Biophysics, Karolinska Institutet, Solna, Sweden; 11 Department of Pediatrics, University of California San Francisco, San Francisco, CA 94143, USA; 12 Department of Cell and Molecular Biology, Karolinska Institutet, Solna, Sweden

**Keywords:** SARS-CoV-2, COVID-19, Angiotensin converting enzyme 2 (ACE2), pericytes, Vasculature, single-cell RNA-sequencing, Endothelial Cells

## Abstract

Humanized mouse models and mouse-adapted SARS-CoV-2 virus are increasingly used to study COVID-19 pathogenesis, so it is important to learn where the SARS-CoV-2 receptor ACE2 is expressed. Here we mapped ACE2 expression during mouse postnatal development and in adulthood. Pericytes in the CNS, heart, and pancreas express ACE2 strongly, as do perineurial and adrenal fibroblasts, whereas endothelial cells do not at any location analyzed. In a number of other organs, pericytes do not express ACE2, including in the lung where ACE2 instead is expressed in bronchial epithelium and alveolar type II cells. The onset of ACE2 expression is organ specific: in bronchial epithelium already at birth, in brain pericytes before, and in heart pericytes after postnatal day 10.5. Establishing the vascular localization of ACE2 expression is central to correctly interpret data from modeling COVID-19 in the mouse and may shed light on the cause of vascular COVID-19 complications.

## Highlights

• Detailed *Ace2/*ACE2 expression patterns are reported for multiple mouse organs
• Vascular *Ace2*/ACE2 expression occurs in pericytes but not endothelial cells
• *Ace2*/ACE2 expression is organotypic and developmentally regulated
• *Ace2/*ACE2 expression in pericytes may suggest their involvement in COVID-19

## Teaser

Through application of scRNA-seq and tissue imaging, we provide a detailed mapping of the expression of *Ace2,* the mouse ortholog of human ACE2/SARS-CoV-2 receptor. We demonstrate prominent but organotypic expression of *Ace2* in certain pericytes, fibroblasts, and epithelial cells, but not in any investigated type of endothelial cells. Knowledge about *Ace2* expression is important for COVID-19 modeling in mouse.

## Introduction

COVID-19 is caused by the severe acute respiratory syndrome coronavirus 2 (SARS-CoV-2). SARS-CoV-2 infects cells through binding of its spike (S) protein to cell surface receptors (angiotensin converting enzyme 2 [ACE2]), followed by S protein cleavage (priming) by the transmembrane serine protease 2 (TMPRSS2) or cathepsins L or B (CTSL and CTSB), fusion of viral and cellular membranes, and viral RNA entry into the cytoplasm (Hoffmann et al., 2020). The initial range of COVID-19 symptoms is explained by expression of ACE2 and TMPRSS2 or CTSL/B in several types of epithelial cells in the nasal cavities, lung, gastrointestinal tract, and eye (Muus et al., 2021; Sungnak et al., 2020; Ziegler et al., 2020). Later in the disease course, some COVID-19 patients however develop additional symptoms, including systemic inflammation, venous and arterial thrombosis with pulmonary embolism, myocardial infarction and stroke, acute kidney injury, and neurological manifestations (for review see Burkert et al., 2021).

The pathophysiological basis of the vascular problems in COVID-19 remains poorly understood, but endothelial injury appears to play an important role. Thrombosis as well as elevated circulating levels of coagulation-promoting factors such as von Willebrand factor (VWF) and pro-inflammatory cyto/chemokines including angiopoietin-2 (Ackermann et al., 2020; Dupont et al., 2021; McGonagle et al., 2021) suggest that severe COVID-19 in part should be considered an endothelial disease (Libby and Lüscher, 2020).

The literature is however conflicting as to whether endothelial cells (ECs) are directly infected by SARS-CoV-2 or not. Endothelial ACE2 expression has been reported based on immunodetection (Hamming et al., 2004; Lovren et al., 2008; Sluimer et al., 2008) or single-cell RNA sequencing (scRNA-seq) studies (Muus et al., 2021), and the presence of SARS-CoV-2 virus particles in ECs in COVID-19 patients has been proposed (Ackermann et al., 2020; Varga et al., 2020), although the data interpretation has also been questioned (Goldsmith et al., 2020). In contrast to these observations, we failed to both detect endothelial *ACE2* expression in human transcriptomic data and infect human ECs *in vitro* (McCracken et al., 2021).

If COVID-19-associated endotheliopathy is not caused by direct infection of ECs by SARS-CoV-2, EC damage may result from their vicinity to other infected cell types, including pulmonary epithelial cells or non-endothelial vascular or perivascular cells (Nicosia et al., 2021). An indirect effect is indeed consistent with induction of endotheliopathy-like responses by plasma from critically ill COVID-19 patients (Queisser et al., 2021; Rauch et al., 2020). Similarly, in studies with epithelial cells and ECs cultured on a chip, only epithelial cells were infected, but with subsequent damage evident also in the ECs (Deinhardt-Emmer et al., 2021). Among potential non-epithelial targets of SARS-CoV-2 infection, pericytes, which are microvascular mural cells in direct contact with the endothelium (Lendahl et al., 2019), have been suggested to express ACE2 (Chen et al., 2020; He et al., 2016, 2018; McCracken et al., 2021; Nicin et al., 2020; Vanlandewijck et al., 2018).

Progress in understanding the cause of the COVID-19 vascular problems is aided by research in experimental animal models, including humanized ACE2 transgenic mice, as standard laboratory mice cannot be infected by current pandemic strains of SARS-CoV-2 (Jiang et al., 2020; McCray et al., 2007; Sun et al., 2020). Through these models it may be possible to address how endotheliopathy occurs, but a prerequisite for such studies is to first establish the specific expression patterns of ACE2 and other proteins that facilitate cellular entry of SARS-CoV-2 in the mouse. In this report, we address the organotypicity of ACE2 expression and demonstrate that pericytes in several organs express ACE2, while ECs do not. These data are relevant for disease modeling of COVID-19 in the mouse and to shed light on possible primary entry points for SARS-CoV-2 in the vasculature.

## Results

### Single-cell RNA sequencing shows that *Ace2* is expressed in brain pericytes but not in endothelial cells

We first assessed the expression and distribution of *Ace2* mRNA in the adult mouse brain. Our previous reports (He et al., 2018; Vanlandewijck et al., 2018) showed that *Ace2* is strongly enriched in brain pericytes and venous vascular smooth muscle cells (VSMCs), qualifying it among the top 15 specific markers for these cells (Figures S1A and S1B and http://betsholtzlab.org/VascularSingleCells/database.html). At lower levels, *Ace2* was also found in arterial/arteriolar VSMC cell clusters (Figure S1C), but not in *Cnn1*-positive arterial VSMC (Figures S1C and S1D). In addition, we noticed *Ace2* expression in a subset of cells annotated as brain fibroblasts (Figure S1C), but their specific expression of numerous pericyte markers, including *Kcnj8*, suggests that these fibroblasts may be related to, or contaminated by, pericytes (Figure S1C).

**Figure 1:**
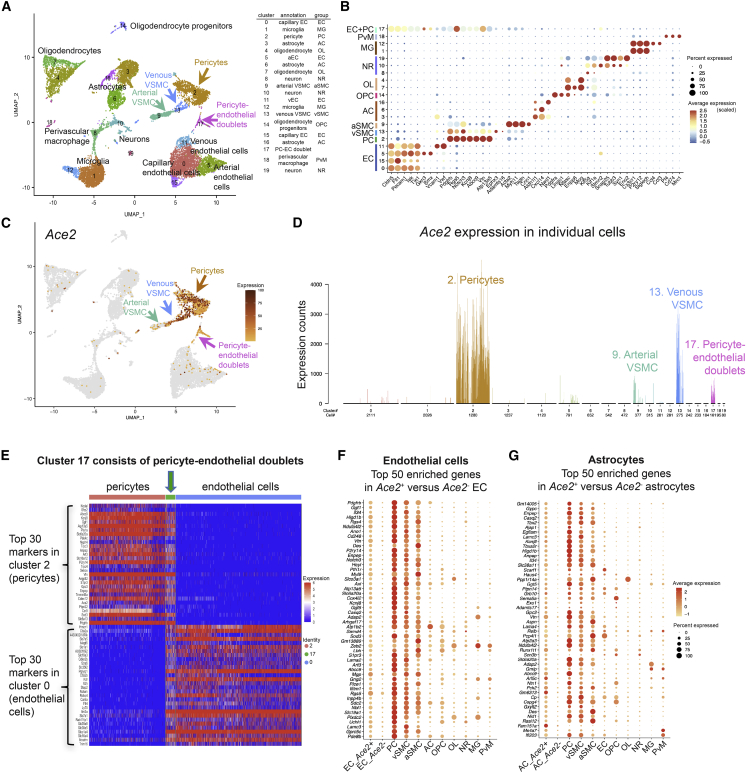
*Ace2* expression in adult mouse brain (A) UMAP display of integrated mouse brain scRNA-seq data. Coloring is based on cluster assignment, and cellular annotations are based on canonical marker expression available at http://betsholtzlab.org/Publications/BrainIntegration/search.html. (B) Dot plot showing the expression of marker genes for each cluster. (C) The same UMAP as in (A) with *Ace2* expression overlay (dark color represents higher expression, and gray color represents *Ace2*-negative cells). (D) Bar plot of the normalized expression levels of *Ace2* in each cluster. Cell type annotations for each cluster are indicated. Individual bars represent single cells and are colored according to cluster assignment together with cell number contributions below the x axis. Arrows of different colors in (A) and (C) indicate the *Ace2*-positive clusters in the UMAP corresponding to the bar plot displays. (E) Expression of endothelial and pericyte enriched transcripts (each top 30 genes). Note the expression of both gene-sets in cluster 17; pericyte-endothelial doublets (green arrow). (F and G) Dot plots showing the expression of the 50 most enriched genes in *Ace2*-positive versus *Ace2*-negative ECs (F) or astrocytes (G). Note the enrichment of differentially expressed genes in mural cell clusters (PC, vSMC, aSMC).

Analysis of an integrated mouse brain scRNA-seq dataset, combining two published (He et al., 2018; Schaum et al., 2018; Vanlandewijck et al., 2018) and one unpublished brain vasculature-focused scRNA-seq datasets (Figures 1A and 1B; see experimental procedures for details on acquisition of the datasets; available for gene-by-gene search at http://betsholtzlab.org/Publications/BrainIntegration/search.html), confirmed that *Ace2* mRNA localized primarily to pericytes and venous VSMCs (Figures 1C and 1D). In addition, we found *Ace2* transcripts in a cluster annotated as pericyte-endothelial cell doublets (Figures 1C and 1D), based on the proportional expression of both endothelial and pericyte transcripts (Figure 1E). Exploring the unbiased assembly of mouse brain single-cell transcriptomes from Zeisel et al. (2018) (http://mousebrain.org/genesearch.html) confirmed *Ace2* expression in four cell clusters representing pericytes and pericyte-endothelial doublets, but importantly it failed to identify *Ace2* transcripts at significant levels in any of ≈250 other brain cell types (Figure S1F and S1G).

Brain endothelial single-cell transcriptomes were largely devoid of *Ace2* mRNA (Figures 1C, D, and S1B). The 1.4% (48 of 3,416) of the ECs displaying *Ace2* mRNA sequence reads (as compared to ≈57% of the pericytes; Figure S1H), however, contained many canonical brain pericyte markers, including *Pdgfrb* (platelet-derived growth factor receptor beta), *Cd248* (endosialin), *Vtn* (vitronectin), *Des* (desmin), *Notch3*, and *Kcnj8* (Figure 1F). The expression of the top 50 *Ace2*-correlated genes in the ECs was generally high in mural cells (pericytes and VSMCs) but low in other cell types, including astrocytes, oligodendrocytes, microglia, and neurons (Figures 1F and S1I).

A similar picture emerged for the rare *Ace2*-positive astrocytes. By assigning the top 50 differentially expressed genes correlating with the presence of *Ace2* in the 13 *Ace2*-positive versus the 2,060 *Ace2*-negative astrocytes (Figure S1H), we again noticed the presence of several pericyte markers, including *Kcnj8, Abcc9, Higd1b, Anpep* (CD13*), Vtn*, and *Cspg4* (NG2), and that the majority of the 50 genes were expressed at corresponding levels in mural cells (Figures 1G and S1J). We conclude from these analyses that pericytes and venous VSMCs are the predominant ACE2-expressing cells in the brain, and the rare occurrence of *Ace2* transcripts in brain cells annotated as ECs or astrocytes is contributed through contamination by mural cell fragments.

### Immunofluorescence analysis reveals ACE2 expression in CNS microvascular mural cells, but not in ECs or arterial VSMCs

**Figure 2:**
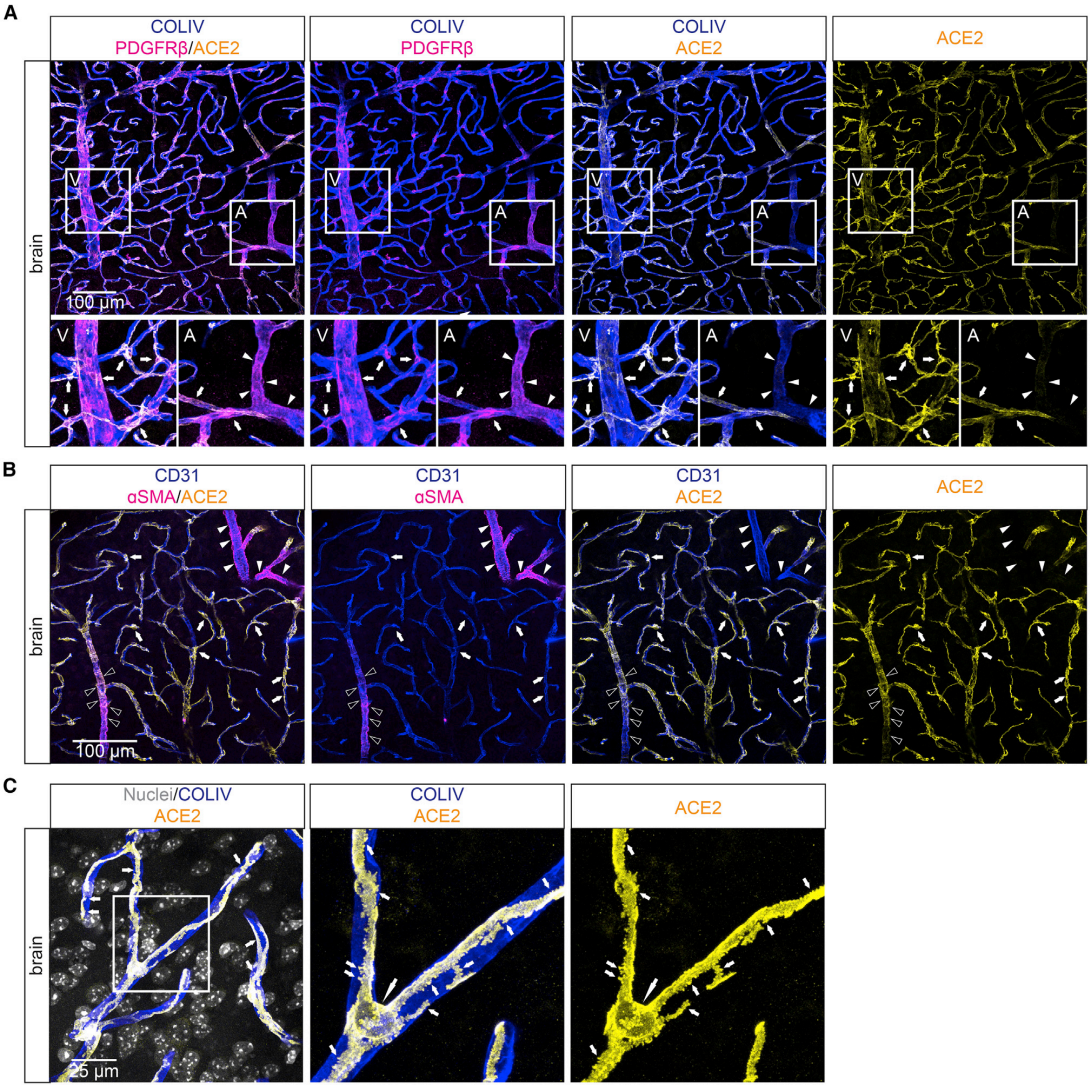
ACE2 IF in adult mouse brain (A–C) IF detection of ACE2 in adult mouse brain in combination with indicated markers. CD31 and collagen type IV (COLIV) are used as markers for the endothelium and basement membrane, respectively. PDGFRβ marks mural cells, and αSMA marks VSMCs. (A) A venule (*V*) and arteriole (*A*) are shown magnified. Arrows indicate pericytes; arrowheads indicate arterioles. Note the similar expression of ACE2 and PDGFRβ in pericytes and venous/venular VSMCs. (B) The absence of ACE2 from large arterioles (arrowheads) and weak presence at terminal arterioles (open arrowheads). (C) High magnification image showing a pericyte with soma and secondary processes. Note that ACE2 staining follows the outline of the pericyte including its secondary processes. The large arrow point at the pericyte cell soma and the small arrows at its primary processes (including peg-sockets). Nuclei are visualized by DAPI or Hoechst 33342. Scale bars are indicated in the figure.

Strong ACE2 immunofluorescence (IF) staining was observed in mural cells associated with capillaries, venules, and veins (Figures 2A, S2A, and S2B), and weakly ACE2-positive VSMCs were present at terminal arterioles in the adult mouse brain cortex (Figures 2A and S2B). In contrast, the alpha-smooth muscle actin (αSMA)-positive mural cells located around arteries and larger arterioles were ACE2-negative (Figure 2B). ACE2 IF fully decorated the pericyte outline (Figure 2C) (Ornelas et al., 2021).

Spinal cord ACE2 was detected in cells with the typical morphology and marker expression of pericytes (Figure S2B). Weaker ACE2 expression was noted also in αSMA-positive VSMCs at terminal arterioles, whereas CNN1-positive VSMCs of larger arteries were ACE2 IF-negative (Figures S2C and S2D). Retinal pericytes were strongly ACE2-positive (Figure S2E) and αSMA-positive VSMCs in small diameter retinal arterioles were weakly ACE2-positive, whereas larger diameter arterioles/arteries were ACE2-negative (Figure S2E). Choriocapillaris, the network of fenestrated capillaries located immediately behind the retinal pigment epithelium, harbored strongly ACE2-positive pericytes (Figure S2E), whereas αSMA-positive vessels feeding this capillary plexus were ACE2-negative (Figure S2E). ACE2-positive pericytes were further found in the ciliary body (Figure S2E). Pericytes located in the extra-ocular skeletal muscle, hence residing outside of the CNS, were ACE2-negative (Figure S2F), while the surface epithelium of the conjunctiva and cornea was ACE2-positive (Figure S2E), confirming recent observations by others (Muus et al., 2021). Together, we observe ACE2 expression in CNS mural cells, while ECs were consistently negative for ACE2 IF at all locations analyzed within the CNS.

### *Ace2* is specifically expressed in pericytes of the heart

**Figure 3:**
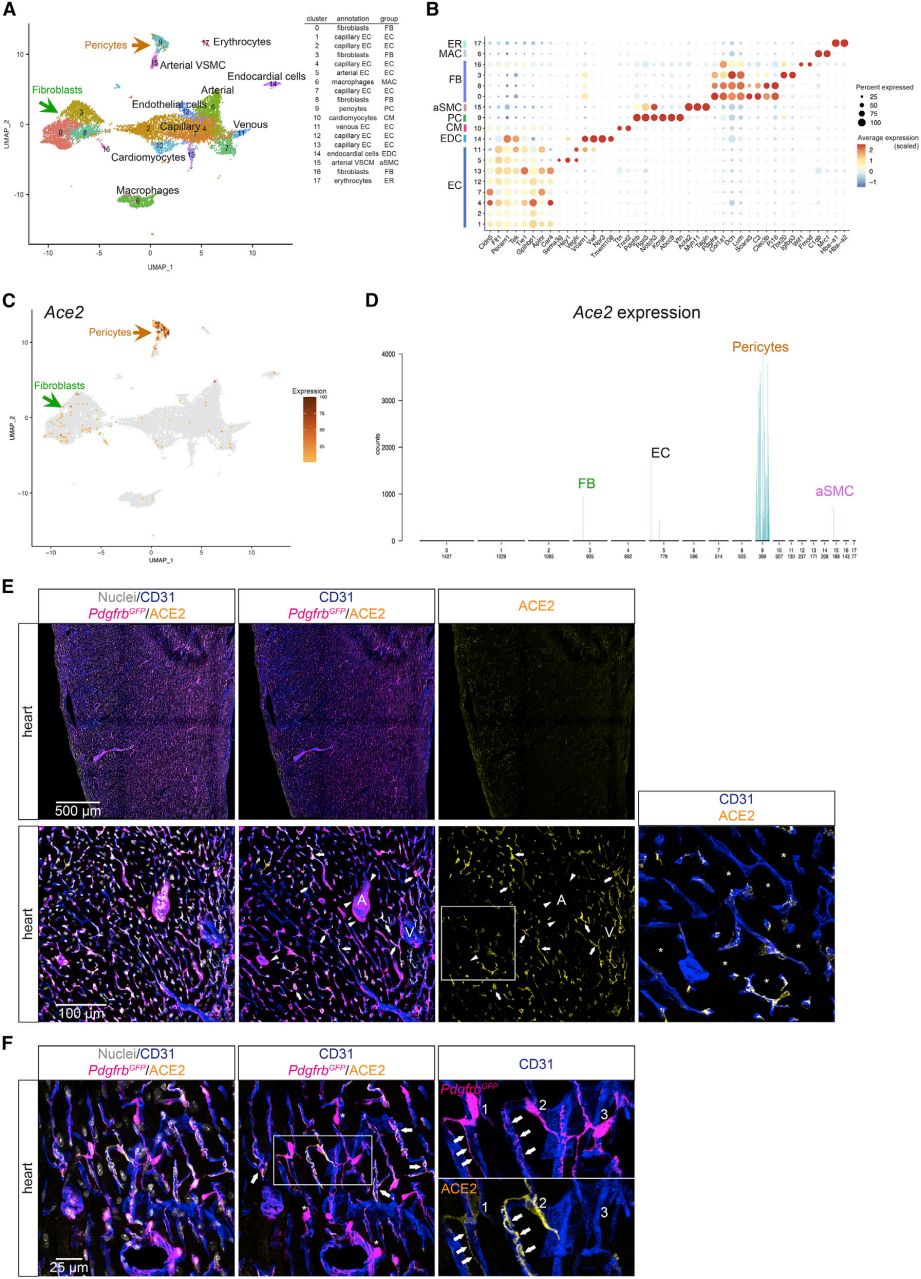
ACE2 expression in the adult mouse heart (A) UMAP display of integrated mouse heart scRNA-seq data. Coloring is based on cluster assignment and cellular annotations are based on canonical marker expression available at http://betsholtzlab.org/Publications/HeartIntegration/search.html. (B) Dot plot showing the expression of marker genes for each cluster. (C) The same UMAP as in (A) with *Ace2* expression overlay (dark color represents higher expression, and gray color represents *Ace2*-negative cells). (D) Bar plot of the normalized expression levels of *Ace2* in each cluster. Cell type annotations for each cluster are indicated. Individual bars represent single cells and are colored according to the cluster assignment together with cell number distribution below the x axis. Arrows of different colors in (A) and (C) indicate the *Ace2*-positive clusters in the UMAP corresponding to the bar plot displays. (E and F) IF detection of ACE2 in adult mouse heart in combination with the indicated markers (*Pdgfrb*^*GFP*^ for pericytes and CD31 as a marker for the endothelium). (E) Overview image (upper panel) and detailed image including an artery (*A*) and vein (*V*) (lower panel). Note the overlapping expression of ACE2 and *Pdgfrb*^*GFP*^ in a proportion of pericytes and that arterial and venous VSMCs are ACE2-negative. In the high-magnification inset, ACE2-negative cardiomyocytes are shown (asterisks) along with ACE2-positive pericytes. (F) High magnification image that shows an example of three neighboring pericytes with different levels of ACE2 expression. Note also the different subcellular distribution of cell membrane-associated ACE2 and cytoplasmic GFP expressed from the *Pdgfrb* promoter (*Pdgfrb*^*GFP*^). Nuclei are visualized by DAPI or Hoechst 33342. Scale bars are indicated in the figure.

It was recently suggested that *ACE2* expression occurs across multiple human cardiac cell types, including cardiomyocytes, ECs, pericytes, fibroblasts, and macrophages (Chen et al., 2020; Muus et al., 2021; Nicin et al., 2020). In an adult mouse heart scRNA-seq data enriched for stromal cells (Muhl et al., 2020), we found prominent expression of *Ace2* in pericytes, but no expression in fibroblasts nor in VSMCs (Figure S3A). The integrated data from the three unpublished and one published (Schaum et al., 2018) datasets (http://betsholtzlab.org/Publications/HeartIntegration/search.html) provided comprehensive coverage of the principal cell types in the mouse heart, including cardiomyocytes, different types of ECs (blood vascular, endocardial, lymphatic), pericytes, VSMCs, and subtypes of fibroblasts and macrophages (Figures 3A and 3B). Of these, only the pericyte cluster showed distinct *Ace2* expression (Figures 3C and 3D). Rare ECs displayed low RNA-seq counts for *Ace2,* but *Ace2*-positive cells showed an enrichment of pericyte transcripts (Figures S3B and S3C). Importantly, Ace2 expression was neither observed in adult cardiomyocytes (Figures 3D, S3B, and S3D) nor in cardiomyocytes from embryonic day (E)9.5, E13.5, E15.5 or newborn (data not shown). Together, this suggests that the low levels of *Ace2* sequences found in rare cardiac ECs were contributed by contaminating pericyte-derived cellular material, similar to our observations in the brain.

Cardiac ACE2 protein IF signal was detected only in cells expressing *Pdgfrb*^*GFP*^ (as a marker for mural cells; Vanlandewijck et al., 2018) and with a location and morphology typical for pericytes: a round cell body and long processes adhering to the ECs (Figures 3E and 3F). In contrast to the CNS, where small arteriolar and venous mural cells were also positive, heart *Ace2* mRNA and protein was found only in capillary pericytes and not in other mural cells (Figures 3C–3E and S3D). Furthermore, cardiac pericyte ACE2 expression was heterogeneous, varying from strongly positive to negative (Figure 3F). Our analysis establishes a subset of the pericytes as the major site of *Ace2* expression in the adult heart, and ACE2 expression was consistently undetectable in mouse cardiac ECs as well as in cardiomyocytes and other cardiac cell types.

### In the lung, airway epithelial cells form the major *Ace2* expression site

**Figure 4:**
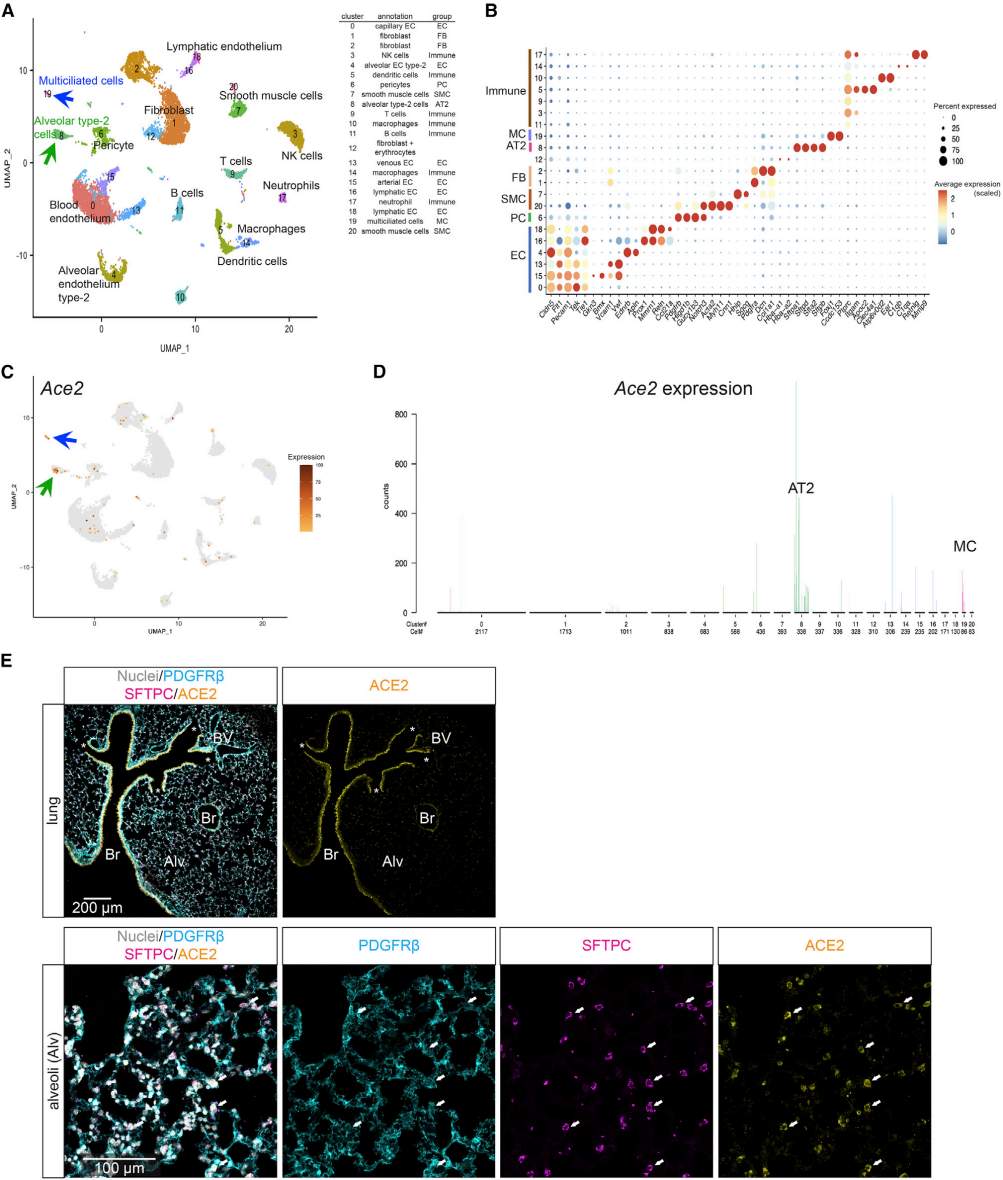
ACE2 expression in adult mouse lung (A) UMAP visualization of the integrated scRNA-seq data from mouse lung. Annotations were based on the expression of canonical markers for each indicated cell type available at http://betsholtzlab.org/Publications/LungIntegration/search.html. (B) Dot plot showing the expression of marker genes for each cluster. (C) The same UMAP as in (A) with *Ace2* expression overlay (dark color represents higher expression, and gray color represents *Ace2*-negative cells). For (A) and (C), green arrows point at the AT-II cell cluster; blue arrows point at the multiciliated cell cluster. (D) Bar plot of the normalized expression levels of *Ace2* in each cluster. Cell type annotations for each cluster are indicated. Individual bars represent single cells and are colored according to the cluster assignment together with cell number distribution below the x axis. (E) IF staining for indicated proteins in adult mouse lung. A prominent ACE2 signal is observed in bronchial epithelium. Asterisks mark end of terminal bronchioles. The lower panels show the same microscopic field of the alveolar region with different labels visualized. Arrows provide landmarks and point at SFTPC-positive AT-II cells. Note the overlap between ACE2 and SFTPC in the alveolar region and lack of ACE2 staining of pericytes (labeled by PDGFRβ). No ACE2 IF signal was observed in ECs. Nuclei are visualized by DAPI or Hoechst 33342. Br: bronchi, BV: blood vessel. Alv: alveolar region Scale bars are indicated in the figure.

Lung epithelial cells are known primary targets for SARS-CoV-2 infection, but it has recently been proposed that lung ECs may also become infected (Ackermann et al., 2020; Teuwen et al., 2020). Our previously published scRNA-seq dataset of pulmonary vascular cells (Vanlandewijck et al., 2018) did not reveal *Ace2* expression in lung vascular cells but showed a distinct *Ace2* signal in epithelial cells co-expressing markers of multiciliation or surfactant secretion (Figure S4A (http://betsholtzlab.org/VascularSingleCells/database.html), pointing to ciliated bronchial epithelial cells and alveolar type II (AT-II) cells as sites of *Ace2* expression. To more precisely define the *Ace2*-expressing cell type, we integrated data from three published (He et al., 2018; Schaum et al., 2018; Vanlandewijck et al., 2018) and two unpublished adult mouse lung scRNA-seq datasets (Figures 4A and 4B; available for gene-by-gene browsing at http://betsholtzlab.org/Publications/LungIntegration/search.html). The integrated data showed significant *Ace2* expression only in AT-II (surfactant protein C [*Sftpc*]-positive) and *Foxj1*-positive multiciliated airway epithelial cells (Figures 4B–4D and S4A). No robust *Ace2* expression was observed in any of the subtypes of vascular ECs (*Pecam1*-positive), mural cells (*Notch3*-positive), fibroblasts (*Pdgfra*-positive, *Notch3*-negative), alveolar macrophages, or other hematopoietic cells (*Ptprc*-positive) (Figure 4D).

A strong ACE2 IF signal was observed in the bronchial epithelium throughout the bronchial tree. In the alveolar region distal to the terminal bronchioles, we found ACE2 IF signal in SFTPC-positive AT-II cells (Figure 4E). We failed to detect ACE2 by IF in any endothelial populations in the lung, including alveolar capillaries and large vessels (Figures 4E and S4B–S4D). CD68-positive alveolar macrophages were also ACE2-negative (Figure S4C). Lung pericytes in the alveolar region were ACE2-negative, while we observed occasional ACE2-positive pericytes in capillaries surrounding larger bronchi (Figure S4D). Finally, pericytes in the lung showed very low levels of *Ace2* mRNA expression, in comparison with pericytes from brain and heart (Figure S4E).

To gain insights into the expression of other proteins of relevance for SARS-CoV-2 infection, we assessed the distribution of mRNAs for the S protein priming proteases *Tmprss2*, *Ctsl*, and *Ctsb* (Figure S4F). We observed *Tmprss2* co-expression with *Ace2* in AT-II cells and bronchial epithelial cells, whereas *Ctsl* and *Ctsb* were more broadly expressed (Figure S4F). In contrast, *Tpmrss2* was not expressed in brain and heart pericytes, which instead exhibited co-expression of *Ace2* with *Ctsb* and *Ctsl* (Figure S4F). Neuropilin-1 (*Nrp1*), a proposed host factor facilitating SARS-CoV-2 infection (Cantuti-Castelvetri et al., 2020; Daly et al., 2020), was again broadly expressed (Figure S4F). In summary, only *Tmprss2* showed restricted expression.

### Different developmental onsets of ACE2 expression in mouse brain, heart, and lung

**Figure 5:**
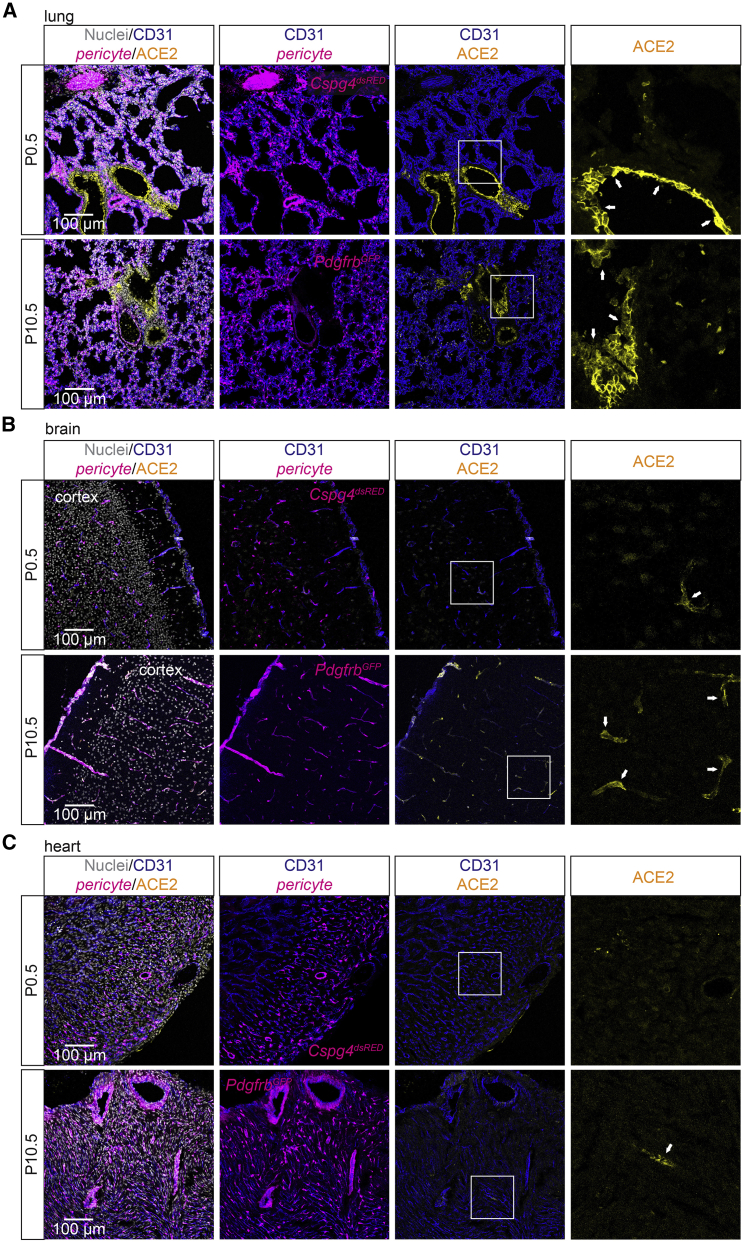
Developmental profile of ACE2 expression in brain, heart, and lung (A) IF staining showing ACE2 expression in the lung at P0.5 and P10.5 in bronchial epithelial cells (arrows). (B) IF staining showing limited ACE2 expression at P0.5 and apparent ACE2 expression at P10.5 in pericytes of the CNS (arrows). (C) IF staining showing no expression of ACE2 at P0.5 and limited ACE2 expression at P10.5 in pericytes of the heart (arrows). Boxed areas are shown magnified in the right panel. Pericytes are indicated by either *Pdgfrb*^*GFP*^ or *Cspg4*^*dsR*^^*ED*^ reporter. No staining was observed in cardiomyocytes. Nuclei are visualized by DAPI or Hoechst 33342. Scale bars are indicated in the figure.

To establish when ACE2 expression was first observed in brain, heart, and lung, we analyzed ACE2 protein distribution in these three organs at postnatal day (P)0.5, P10.5, and in adult mice. At P0.5, we observed extensive ACE2 expression in the lung bronchial cells, occasional ACE2-expressing brain pericytes, but no ACE2-positive heart pericytes or cardiomyocytes (Figures 5A–5C). AT-II cells showed occasional expression of ACE2 already at P0.5 (Figure S5A). At P10.5, ACE2 IF was observed in CNS pericytes as well as in the bronchial epithelial cells, but only occasionally in heart pericytes (Figures 5A–5C). Together, these data indicate differential onsets of ACE2 expression in brain, heart, and lung.

### ACE2 distribution in other organs

**Figure 6:**
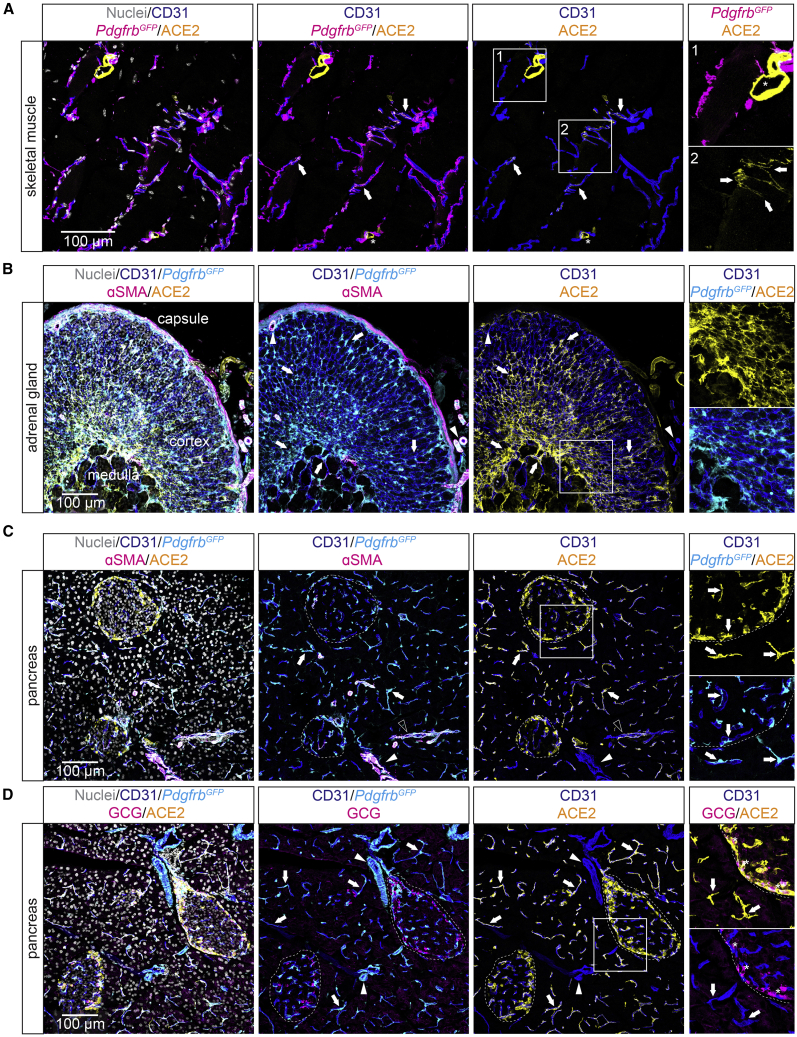
ACE2 expression in pancreas, adrenal gland, and skeletal muscle (A) IF staining for indicated proteins and *Pdgfrb*^*GFP*^ reporter showing limited expression of ACE2 in pericytes of the skeletal muscle. Pericytes positive for ACE2 are indicated by arrows. The asterisk in box 1 indicates an ACE2-positive nerve. (B) IF staining for indicated proteins and *Pdgfrb*^*GFP*^ reporter showing strong expression of ACE2 in cells of the medulla and cortex of the adrenal gland. Arrows highlight ACE2-positive cells (pericytes and other perivascular cells). Arrowheads indicate ACE2-negative arterial VSMCs. (C and D) IF staining for indicated proteins showing ACE2-positive pericytes of the pancreas. Pericytes of the endocrine and exocrine pancreas that are positive for ACE2 are indicated by arrows. VSMCs of terminal arterioles are positive for ACE2 as indicated by open arrowhead (C), and ACE2-negative arteriolar/arterial VSMCs are highlighted by closed arrowhead. Islets of Langerhans (endocrine pancreas) are indicated by dashed circles. (D) ACE2-positive α-cells of the pancreas islets (marked by staining for glucagon, GCG) are indicated by asterisks (right panel). Boxed areas are shown magnified in the right panel. Nuclei are visualized by DAPI or Hoechst 33342. Scale bars are indicated in the figure.

We next analyzed ACE2 distribution by IF in several other organs to obtain further insights into pericyte organotypicity. In skeletal muscle, occasional pericytes were ACE2-positive (Figure 6A), however at lower frequency compared to the heart or brain. In the adrenal gland of *Pdgfrb*^*GFP*^ mice (to visualize mural cells), robust ACE2 IF was observed in pericytes and perivascular cells, likely fibroblasts, located in both the cortex and the medulla (Figure 6B). In pancreas, there was ubiquitous expression of ACE2 in pericytes around the exocrine ducts and in endocrine islets. Moreover, ACE2 expression was found in glucagon-producing pancreatic α-cells (Figures 6C and 6D), the latter in keeping with previous reports (Coate et al., 2020; Fignani et al., 2020).

**Figure 7:**
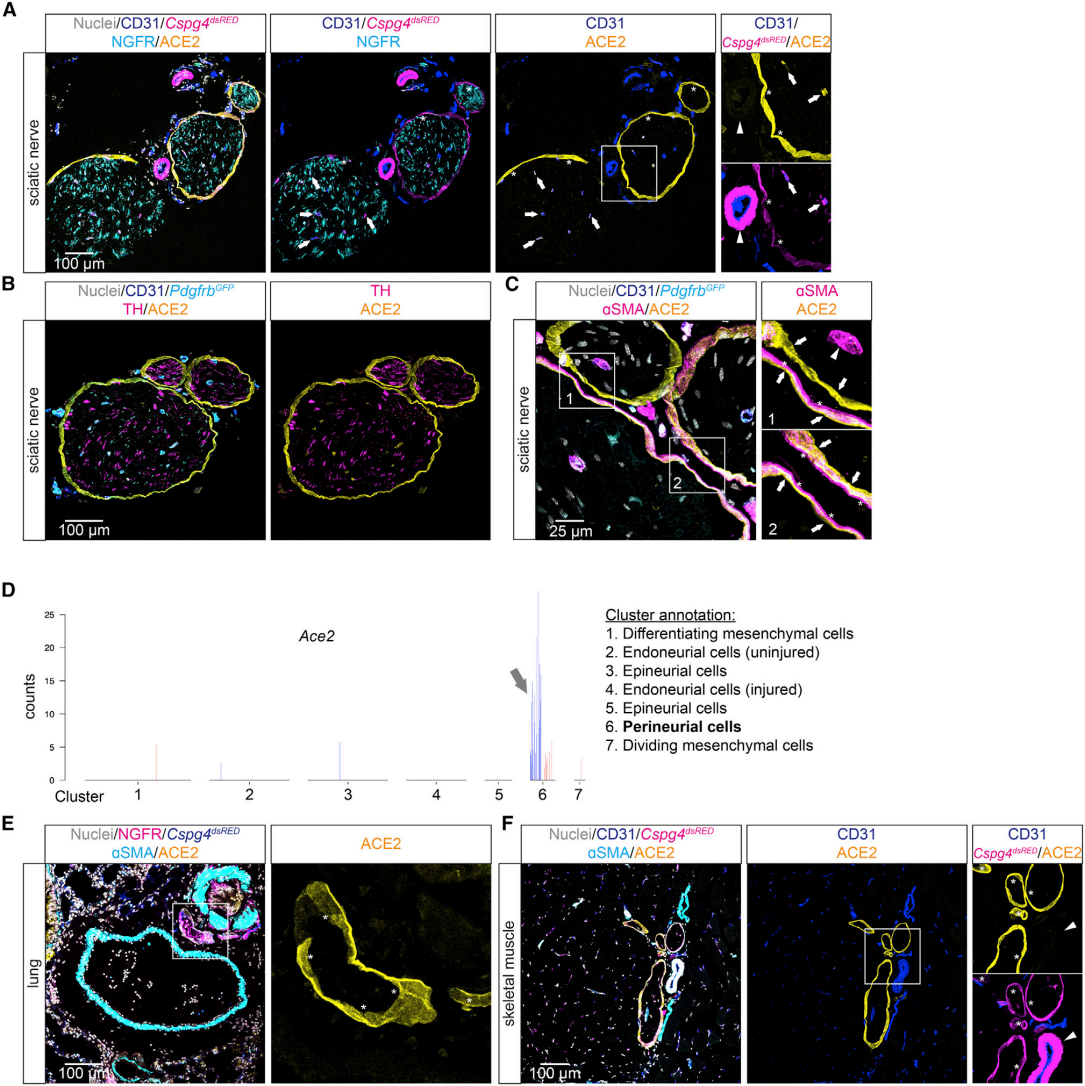
ACE2 expression in perineurial fibroblasts (A–C) IF staining for indicated proteins in mouse sciatic nerve. (A) ACE2-positive pericytes of sciatic nerve at endoneurial capillaries are indicated by arrows, ACE2-positive perineurial fibroblasts are indicated by asterisks. Arteriolar/arterial VSMCs negative for ACE2 are indicated by arrowhead. (B) Overview image showing the identity of sciatic nerve by co-staining with tyrosine hydroxylase (TH). (C) High-magnification image showing the ACE2-positive perineurial fibroblast layer (arrows) in closeness, proximity to the αSMA-positive cell layer (asterisks). Arrowhead indicates ACE2-negative arteriolar/arterial VSMCs in box1. (D) Transcriptional data from Carr et al. (2019) showing *Ace2* expression in perineurial cells (fibroblasts), visualized as bar plot. The blue and red bars indicate cells from uninjured control samples and injured samples, respectively (see original publication for cluster annotation). (E and F) IF staining for indicated proteins in adult lung (E) and skeletal muscle (F) showing ACE2-positive perineurial fibroblast (asterisks) at peripheral nerves indicated by NGFR (nerve growth factor receptor) (E) or *Cspg4*^*dsR*^^*ED*^ (F) staining, the latter to visualize pericytes. Arrowhead in (F) indicates ACE2-negative arteriolar/arterial VSMCs. Boxed areas are shown magnified. Nuclei are visualized by DAPI or Hoechst 33342. Scale bars are indicated in the figure.

In the sciatic nerve, pericytes in the endoneurium were ACE2-positive, and in addition, we observed strongly ACE2 IF-positive cells surrounding nerve fascicles (Figures 7A–7C); these cells had the expected location of perineurial fibroblasts (Bunge et al., 1989). Analysis of scRNA-seq datasets from peripheral nerves (Carr et al., 2019; Gerber et al., 2021) confirmed *Ace2* expression in adult perineurial fibroblasts (Figure 7D). ACE2-positive perineurial fibroblasts were also observed in the lung and skeletal muscle (Figures 7E and 7F). In none of these organs did we observe ACE2 expression in ECs. Finally, we assessed ACE2 distribution by IF in the GI tract (Figures S6A–S6F). ACE2 expression was observed in pericytes in tongue muscle, stomach mucosa, and colon muscularis, while we did not find any ACE2 expression in pericytes from esophagus, duodenum, or ileum. ACE2 expression was observed in epithelial cells in tongue, esophagus, duodenum, and ileum (Figures S6A–S6F). At the mRNA level, intestinal enterocytes showed high levels of *Ace2* expression (Figure S7).

## Discussion

A bone of contention in recent discussions is whether COVID-19-associated endothelial injury is caused by direct virus infection of ECs or is a secondary consequence of infection of neighboring cells. A number of studies favor ACE2 expression by ECs (Ackermann et al., 2020; Lovren et al., 2008; Muus et al., 2021; Varga et al., 2020), whereas other studies advocate pericytes as a major site of ACE2-expression (Chen et al., 2020; He et al., 2016, 2018; Nicin et al., 2020; Vanlandewijck et al., 2018). In line with the latter notion, a recent study (McCracken et al., 2021) reported that the low-level *ACE2* mRNA expression observed in human ECs (Muus et al., 2021) is likely caused by pericyte contamination. Furthermore, infection of pericytes may underlie the neuropathology observed in COVID-19 patients (Bocci et al., 2021).

The data presented here show that microvascular mural cells are the predominant site of ACE2 expression in mouse brain and heart vasculature. ACE2 mRNA and protein expression was confined to pericytes, venous VSMCs, and *Cnn1*-negative arteriolar VSMCs in the CNS and to pericytes in the heart. ACE2-expressing pericytes were also observed in tongue, stomach, and colon. However, in none of these organs were ECs found to express ACE2. ACE2 is however not a universal pericyte protein. In the heart about half of the pericytes were ACE2-positive, and in the lung, there were a few ACE2-positive pericytes in capillaries surrounding large airways, while lung alveolar capillary pericytes were ACE2-negative. Similarly, ACE2-negative pericytes were observed in the duodenum and ilium.

The observation that pericytes constitute the principal vascular, albeit organotypically, ACE2-expressing cells in both mice and humans (Bocci et al., 2021; McCracken et al., 2021) indicates an evolutionary conservation of ACE2 expression in mural cells. ACE2 expression in pericytes may also have bearings on why conditions such as diabetes and obesity are risk factors for COVID-19 (Apicella et al., 2020). Common to these conditions is a dysfunctional leaky vasculature (Lindenmeyer et al., 2007; Singh et al., 2008). Pericytes are normally located behind the endothelial lining, protected from direct contact with the blood stream, and it thus appears unlikely that, in a healthy sealed vasculature, SARS-CoV-2 virus circulating in the blood stream would be able to be transported through the endothelial lining via *trans*- or paracellular routes to reach the pericytes (Claesson-Welsh, 2015). However, in a leaky vasculature, the SARS-CoV-2 virus may get access to and infect pericytes, possibly rendering them dysfunctional, leading to activation of pro-inflammatory and pro-thrombotic responses in neighboring ECs. Indeed, in a recent analysis of the endothelial reaction to pericyte loss, upregulation of several pro-inflammatory mediators, including angiopoietin-2 and VWF, was found (Andaloussi Mäe et al., 2020).

In addition to pericytes, we identify several other cell types expressing ACE2. In the lung, the major sites of ACE2 expression were instead the AT-II cells and *Foxj1*-positive multiciliated bronchial epithelial cells, in keeping with a previous report (Ziegler et al., 2020). Perineurial fibroblasts were also ACE2-positive, which is interesting in the light of the neurological manifestations of COVID-19, as the perineurial fibroblasts form a metabolically active permeability barrier and provide structural integrity to the nerve fascicle. In the GI tract, epithelial cells in the tongue, esophagus, duodenum, and ilium were ACE2-positive, which may be of interest in relation to the gastrointestinal manifestations of COVID-19 (Guo et al., 2021) and to SARS-CoV-2 mRNA levels in wastewater (Bertels et al., 2022).

The data presented here are of relevance for COVID-19 research in mice. As standard laboratory mice cannot be infected by SARS-CoV-2, humanized ACE2 transgenic mice and mouse-adapted SARS-CoV-2 virus have been developed to circumvent the tropism problem. While some humanized mouse models only express hACE2 in the airways, they may not recapitulate the full spectrum of pathology, while mouse models where the *hACE2* gene has been inserted into the mouse *Ace2* locus (Sun et al., 2020) may provide a better opportunity to assess infectability of pericytes and possible vascular consequences, as *hACE2* should be expressed like mouse *Ace2*. It will be interesting to explore in such models whether pericytes can be infected and whether infection would be facilitated by a breakdown of vascular integrity. The apparent lack of ACE2 expression in mouse cardiomyocytes, which contrasts with ACE2 being expressed in human cardiomyocytes and in cardiomyocytes derived from human pluripotent cells (Chen et al., 2020; Muus et al., 2021; Nicin et al., 2020; for review see Yiangou et al., 2021), underscores that there may be differences between human and mice regarding ACE2 expression, a notion that should be kept in mind when interpreting COVID-19 mouse experiments and translating data to the human situation.

Organoids are increasingly used in COVID-19 research (Geurts et al., 2021), and in a recent study, SARS-CoV-2 was shown to infect and multiply in human microvascular organoids composed of both endothelial and mural cells (Monteil et al., 2020), but the exact cellular tropism of the virus in this model remains to be established. Support for a pericyte-like cell as a nodal point of infection comes from analysis of neural organoids, where cortical organoids without pericyte-like cells were less efficiently infected (Wang et al., 2021). In conclusion, our data on ACE2 expression constitute a platform for further research in mouse and organoid models to better understand the vascular pathologies associated with COVID-19.

## Experimental procedures

### Single-cell RNA-seq data analysis

#### Mouse brain single-cell data integration

Mouse brain datasets were integrated from two internal brain single-cell projects and one published (the Tabula Muris brain resource) (Schaum et al., 2018). The internal datasets included one unpublished and one previously published brain vasculature dataset (GSE98816, GSE99058) (He et al., 2018). The cells were from 10- to 19-week-old C57Bl6 mice. The single-cell RNA-seq was conducted using the SMART-Seq2 protocol (Picelli et al., 2014) and the 10X Genomics protocol. Data processing and clustering were performed using the Seurat package (v. 3.1.1). Cells containing fewer than 200 expressed genes were filtered out. For the SMART-Seq2 data, cells that generated fewer than 50,000 reads were filtered out; for the droplet platform, cells containing fewer than 1,000 UMIs were filtered out. Furthermore, genes that were expressed by fewer than three cells in a dataset were removed. After removing low-quality cells from the dataset, the data were normalized using the LogNormalize function in the Seurat package, by which feature counts for each cell are divided by the total counts for that cell and multiplied by a scale factor (1 million) and then logarithmically transformed. For integration of different datasets, the integration workflow “Reciprocal PCA” in the Seurat package was implemented, which integrated overall datasets using the mutual nearest neighbor cell pairs that shared a common set of molecular features in their PCA spaces. We obtained a total of 12,940 cells and 17,779 genes in the integrated brain dataset.

#### Mouse heart single-cell data integration

scRNA-seq data were obtained from internal mouse heart single-cell projects (GSE149301) and the published Tabula Muris heart dataset (Schaum et al., 2018), collectively including diverse cell types in the heart. All samples were obtained from 6- to 20-week-old C57Bl6 mice. Data integration and clustering analysis were performed with the same methods as for the mouse brain data described above. After integration, we obtained a total of 18,378 genes and 10,101 cells for downstream analysis. The function “FindClusters” in the Seurat package was used to identify the clusters with a resolution parameter of 0.5.

#### Mouse lung single-cell data integration

The mouse lung datasets were obtained from internal lung single-cell projects (GSE99235) and the published Tabula Muris lung resource (Schaum et al., 2018). All samples were from 10- to 19-week-old C57Bl6 mice. Data integration and clustering analysis for the lung were performed with the same methods as for the mouse brain data described above. We obtained a total of 20,114 genes and 11,085 cells in the integrated lung dataset.

#### Identification of pericyte contamination of other cell types

To identify pericyte contamination in other cell types, including ECs, fibroblast-like cells, and cardiomyocytes, we examined the expression of pericyte-specific markers, including *Kcnj8*, *Pdgfrb*, and *Abcc9*. Their expression profiles in *Ace2*-positive and *Ace2*-negative cells were compared in a random selection of equal numbers of cells, and the heatmap results were visualized with pheatmap package (version 1.0.12) in R software. Also, *Ace2*-positive and *Ace2*-negative cells within the individual cell types were compared, and the top 50 enriched genes in the *Ace2*-positive cells were visualized using the DotPlot function in Seurat (version 3.1.1).

### Mice

The following mouse strains were used: *Cspg4*-DsRed (The Jackson Laboratory), Tg(Cspg4-DsRed.T1)1Akik/J, *Pdgfrb*-GFP (Gensat.org, Tg(Pdgfrb-eGFP) JN169Gsat/Mmucd) (He et al., 2016), *Cldn5*-GFP (Tg(Cldn5-GFP)Cbet/U), *Acta2*^*GFP*^ (The Jackson Laboratory, Tg(*Acta2*-GFP)1Pfk), *Prox1-GFP* (Tg(Prox1-EGFP)KY221Gsat/Mmcd). All mice were backcrossed on a C57BL6/J genetic background. Mice from P0 to 6 months of age and of both sexes were used for experiments. Animal protocols were approved by either the Uppsala Ethical Committee on Animal Research (permit numbers C224/12, C115/15, C111515/16) or by the Stockholm/Linköping Ethical Committee on Animal Research (permit ID 729). All animal experiments were carried out in accordance with their guidelines.

### Immunofluorescence staining

Mice under full anesthesia were euthanized by either transcardial perfusion with Hanks balanced salt solution (HBSS, cat. #14025092, GIBCO) followed by 4% buffered formaldehyde (cat. #02178, Histolab) or cervical dislocation. Fixation, sectioning, and antibody incubations were performed according to standard procedures, and details are described in the supplemental information.

## Author contributions

C.B. conceived the COVID-19-pericyte hypothesis and developed it together with T.A., X-R.P., and U.L. L.H. performed the bioinformatics analysis. L.H. and Y.S. designed the scRNA-seq meta-analysis pipeline and used it together with R.P. L.M. and M.A.M. conducted the ACE2 immunofluorescence analysis. L.M., J.L., G.G., L.Z., Y.X., S.L., G.M., S.S., A.O., M.R., A.A., J.B., M.V., K.B., E.H., K.A.H., Y.H., P.E., and T.M. provided unpublished scRNA-seq data. C.B. assembled the data. C.B. and U.L. wrote the manuscript with significant input from L.M., L.H., and M.A.M. All authors reviewed and edited the text.

## Conflicts of interest

C.B. holds a research grant from AstraZeneca BioPharmaceuticals R&D. X-R.P is an employee of AstraZeneca BioPharmaceuticals R&D. U.L. is a member of the Editorial Board for Stem Cell Research and holds a research grant from Merck KGaA but no personal remuneration. The other authors declare no competing interests.

## Data Availability

The single-cell transcriptomic data included in the paper are freely available as searchable databases at http://betsholtzlab.org/VascularSingleCells/database.html, http://betsholtzlab.org/Publications/BrainIntegration/search.html, http://betsholtzlab.org/Publications/HeartIntegration/search.html, and http://betsholtzlab.org/Publications/LungIntegration/search.html. The single-cell RNA sequencing raw data for the study can be accessed from NCBI’s Gene Expression Omnibus database through the accession numbers GSE128509, GSE155387, GSE197360, GSE197529, GSE198592.

## Supplemental information

Document S1. Supplemental experimental procedures, Figures S1–S7, and Table S1

Document S2. Article plus supplemental information

## Footnotes

Supplemental information can be found online at https://doi.org/10.1016/j.stemcr.2022.03.016.
