# Supplementary material for: The SARS-CoV-2 receptor ACE2 is expressed in mouse pericytes but not endothelial cells: Implications for COVID-19 vascular research
Source: Stem Cell Reports. 2022 Apr 21;17(5):1089–104. doi: 10.1016/j.stemcr.2022.03.016 (PMC9022216; doi:10.1016/j.stemcr.2022.03.016)
Supplement: Document S1. Supplemental experimental procedures, Figures S1–S7, and Table S1 [file mmc1.pdf]

Stem Cell Reports, Volume *17*

Supplemental Information

The SARS-CoV-2 receptor ACE2 is expressed in mouse pericytes but not endothelial cells: Implications for COVID-19 vascular research

Lars Muhl, Liqun He, Ying Sun, Maarja Andaloussi Mäe, Riikka Pietilä, Jianping Liu, Guillem Genové, Lei Zhang, Yuan Xie, Stefanos Leptidis, Giuseppe Mocci, Simon Stritt, Ahmed Osman, Andrey Anisimov, Karthik Amudhala Hemanthakumar, Markus Räsänen, Emil M. Hansson, Johan Björkegren, Michael Vanlandewijck, Klas Blomgren, Taija Mäkinen, Xiao-Rong Peng, Yizhou Hu, Patrik Ernfors, Thomas D. Arnold, Kari Alitalo, Urban Lendahl, and Christer Betsholtz

Figure S1

A

Top 20 enriched genes in group (G)1 vs. G2
G1 = PC, vSMC
G2 = aaSMC, aSMC, MG, FB1, FB2, OL, EC1, EC2, EC3, vEC, capEC, aEC, AC

#	Symbol	G1_avg.	G2_avg.	Fold(G1/G2)
1	Sebox	57.02	0.36	157.05
2	Nts	39.39	0.39	100
3	Nodal	104.94	1.19	88.47
4	Iqsec3	18	0.24	76.22
5	Atp13a5	4185.95	102.73	40.75
6	5033404E19Rik	81.32	2.02	40.32
7	Art3	811.79	22.17	36.62
8	Nxph4	34.04	1.22	27.93
9	Vtn	15514.65	559.46	27.73
10	Sfrp2	49.54	1.85	26.73
11	Arl5c	14.27	0.58	24.73
12	Slc6a20a	790.97	32.4	24.41
13	Tmem26	10.08	0.42	24
14	Plxdc1	338.27	14.86	22.77
15	Ace2	403.42	18.01	22.4
16	Abcc9	741.44	33.8	21.93
17	Pla1a	305.17	14.18	21.53
18	P2ry14	1222.11	57.62	21.21
19	Kcnj8	1105.9	52.58	21.03
20	Uchl1	146.45	7.17	20.42

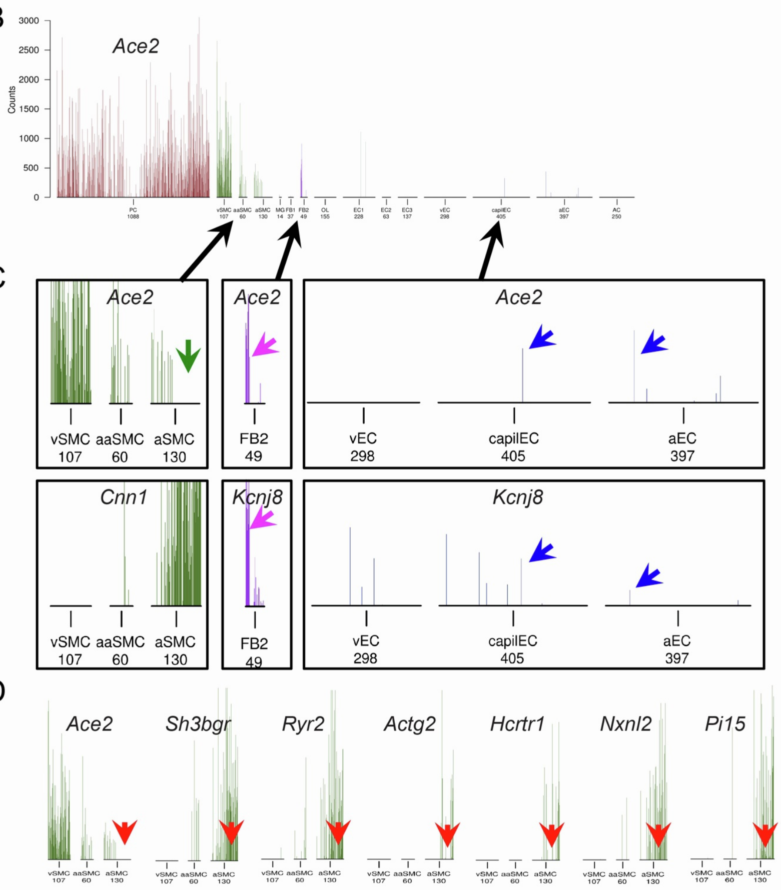

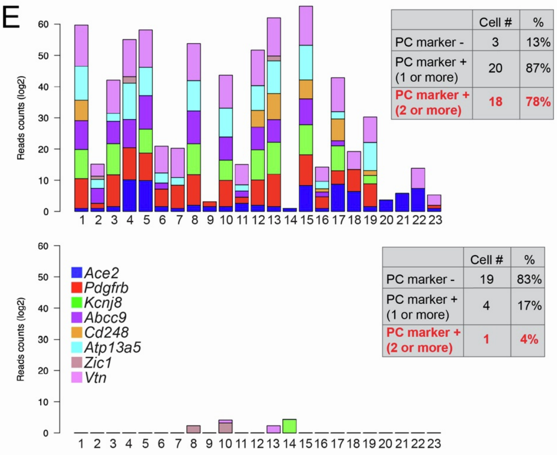

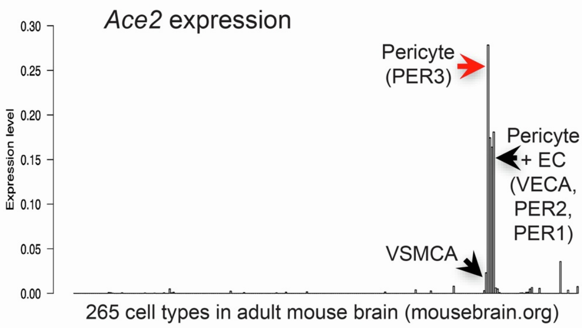

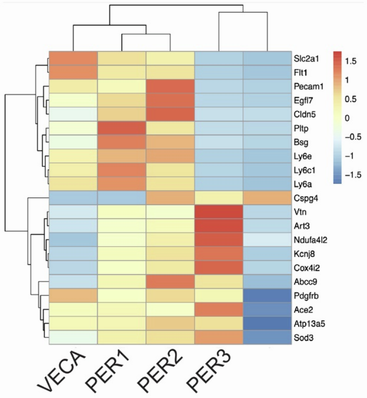

H

	Total	*Ace2+*	*Ace2-*	***Ace2+%***
EC	3416	48	3368	**1,41%**
PC	1280	729	551	**56,95%**
vSMC	275	133	142	**48,36%**
aSMC	377	60	317	**15,92%**
AC	2073	13	2060	**0,63%**
OPC	242	0	242	**0,00%**
OL	1662	15	1647	**0,90%**
NR	867	8	859	**0,92%**
MG	2307	5	2302	**0,22%**
PvM	95	0	95	**0,00%**

I

Heat map overview of the top 50 genes enriched in *Ace2*+ versus *Ace2*- endothelial cells

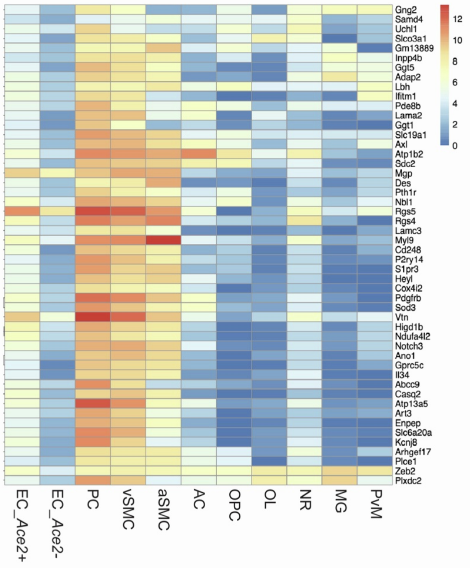

Pearson correlation to the *Ace2*+ EC

	correlation
EC_*Ace2+*	1,000
EC_*Ace2-*	0,861
vSMC	0,601
PC	0,541
aSMC	0,445
NR	0,347
AC	0,228
PvM	0,179
MG	0,052
OL	-0,046
OPC	-0,191

J

Heat map overview of the top 50 genes enriched in *Ace2*+ versus *Ace2*- astrocytes

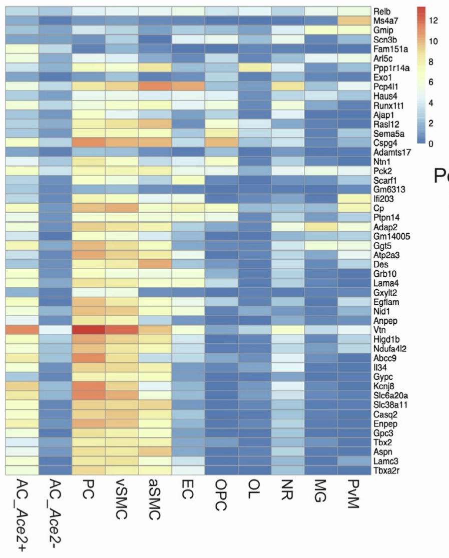

Pearson correlation to the *Ace2*+ AC

	correlation
AC_*Ace2+*	1,000
PC	0,725
vSMC	0,708
AC_*Ace2-*	0,480
aSMC	0,461
NR	0,393
EC	0,255
MG	0,099
OL	-0,015
PvM	-0,079
OPC	-0,113

Figure S1. *Ace2* mRNA in brain cells other than mural cells is due to pericyte contamination. Related to Figure 1.

(A) Top enriched transcripts (red arrow indicates *Ace2* at 15th place) in pericytes and venous VSMCs as compared to other vascular and perivascular cell types deduced from the brain vascular scRNA-seq database. (B) Bar plot excerpt from http://betsholtzlab.org/VascularSingleCells/database.html showing the expression of *Ace2* in different vascular and perivascular cell types. (C) Magnified view of indicated part of A comparing the expression of *Ace2*, *Cnn1* and *Kncj8*. Abbreviations: PC, Pericytes; SMC, Smooth muscle cells; MG, Microglia; FB, Vascular fibroblast-like cells; OL, Oligodendrocytes; EC, Endothelial cells; AC, Astrocytes; v, venous; capil, capillary; a, arterial; aa, arteriolar. (D) Excerpts from http://betsholtzlab.org/VascularSingleCells/database.html showing bar plots across VSMC subtypes of arterial VSMC-specific genes that anti-correlate with *Ace2*. Red arrows point at the *Ace2*-negative part of the cluster. Cell type abbreviations: SMC, Smooth muscle cells; v, venous; a, arterial; aa, arteriolar. (E) Expression of selected known brain pericyte specific markers in 23 *Ace2*-positive ECs (top) and 23 randomly selected *Ace2*-negative ECs (bottom). Colors indicate pericyte marker as shown, and the frequency of their expression is provided in the tables. (F) *Ace2* expression in mouse brain single-cell transcriptomes from (Zeisel et al., 2018) (http://mousebrain.org/genesearch.html). Abbreviations PER - pericyte; EC - endothelial cells; VECA - vascular endothelial cells, arterial; VSMCA - vascular smooth muscle cells, arterial. (G) Heat map display of the expression of *Ace2*-positive, top 10 pericyte and top 10 endothelial markers, showing that PER1-2, VECA are pericyte contaminated. (H) Statistics of the number of cells, *Ace2*-positive cell percentage in each brain cell type. (I-J) Heat map overview of the top 50 genes enriched in *Ace2*-positive versus *Ace2*-negative ECs (I) or astrocytes (J), respectively. The *pearson* correlation was calculated based on the 50 genes.

Figure S2

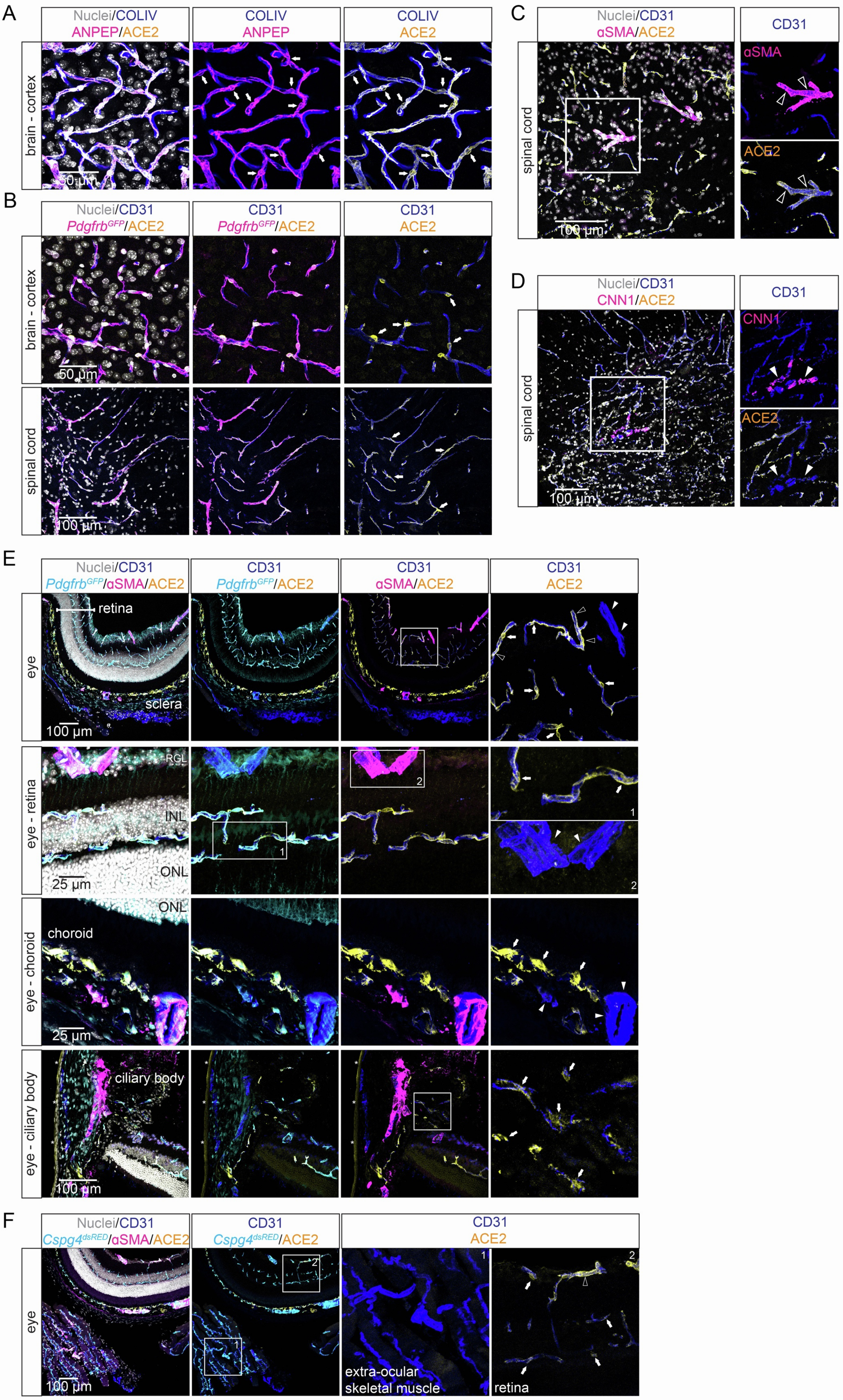
A
Nuclei/COLIV
ANPEP/ACE2
COLIV
ANPEP
COLIV
ACE2
brain - cortex
50 µm
B
Nuclei/CD31
Pdgfrb^GFP/ACE2
CD31
Pdgfrb^GFP/ACE2
CD31
ACE2
brain - cortex
50 µm
spinal cord
100 µm
C
Nuclei/CD31
αSMA/ACE2
CD31
αSMA
ACE2
spinal cord
100 µm
D
Nuclei/CD31
CNN1/ACE2
CD31
CNN1
ACE2
spinal cord
100 µm
E
Nuclei/CD31
Pdgfrb^GFP/αSMA/ACE2
CD31
Pdgfrb^GFP/ACE2
CD31
αSMA/ACE2
CD31
ACE2
eye
retina
sclera
100 µm
eye - retina
RGL
INL
ONL
25 µm
eye - choroid
ONL
choroid
25 µm
eye - ciliary body
ciliary body
100 µm
F
Nuclei/CD31
Cspg4^dsRED/αSMA/ACE2
CD31
Cspg4^dsRED/ACE2
CD31
ACE2
eye
100 µm
extra-ocular
skeletal muscle
retina

Figure S2. ACE2 protein expression in the adult mouse brain cortex, spinal cord and eye. Related to Figure 2.

(A-D) Confocal microscopy images of sections from brain cortex (A, B upper panel) and spinal cord (B lower panel, C-D) IF stained using antibodies and the *Pdgfrb*^{GFP} marker as indicated (ANPEP, aka CD13 and *Pdgfrb*^{GFP} for pericytes, αSMA and CNN1 for VSMCs). Note the strong ACE2 staining of capillary pericytes and the co-expression with ANPEP (A) and *Pdgfrb*^{GFP} (B), indicated by arrows. Mural cells in terminal arterioles are weakly ACE2-positive (C, open arrowheads), whereas mural cells of larger arterioles are ACE2-negative (arrowheads in D). (E-F) Confocal microscopy images from the eye, IF stained using antibodies and *Pdgfrb*^{GFP} or *Cspg4*^{dsRED} markers as indicated (*Pdgfrb*^{GFP} and *Cspg4*^{dsRED} for pericytes and αSMA for VSMCs). Boxed areas are shown magnified in the right panel. Note the strong expression of ACE2 in pericytes of the retina (E, upper two panels) as well as of the choriocapillaris and of the ciliary body (E, lower two panels). Similar as to the brain and spinal cord, terminal arterioles are weakly ACE2-positive (open arrowheads), while large arterioles are negative for ACE2 expression (arrowheads). Asterisks in lower panel indicate ACE2-positive cells of the surface epithelium of the conjunctiva and cornea. (F) Overview image including extra-ocular skeletal muscle. Note the absence on ACE2 expression in pericytes of the extra-ocular muscle (boxed area 1), in contrast to retinal pericytes (arrows in boxed area 2). Nuclei are visualized by DAPI or Hoechst 33342. Scale bars as indicated in the figure.

Figure S3

A

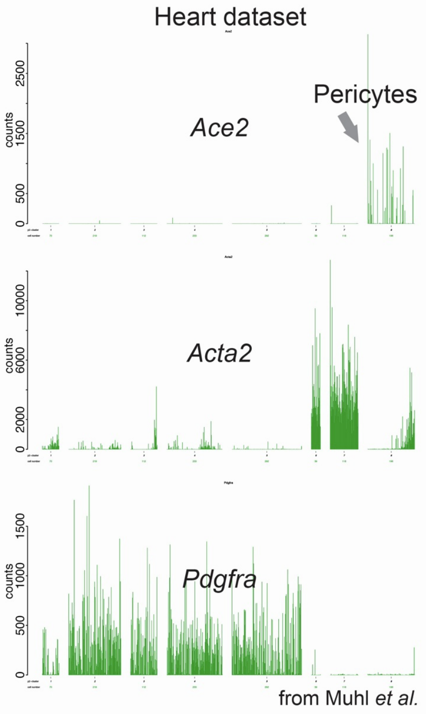

B

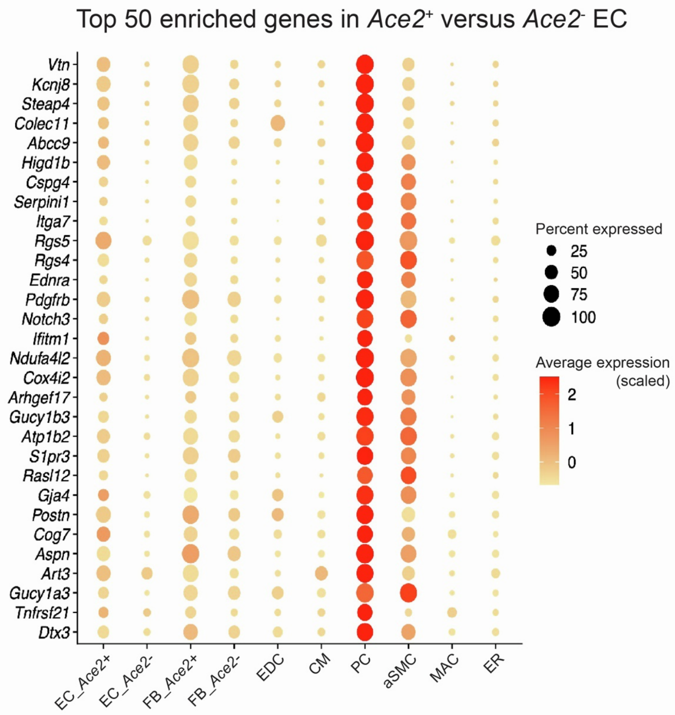

C

	Total	*Ace2+*	*Ace2-*	***Ace2+%***
EC	5017	18	4999	**0,36%**
FB	2978	63	2915	**2,12%**
EDC	208	1	207	**0,48%**
CM	307	3	304	**0,98%**
PC	369	137	232	**37,13%**
aSMC	188	6	182	**3,19%**
MAC	596	2	594	**0,34%**
ER	77	1	76	**1,30%**

D

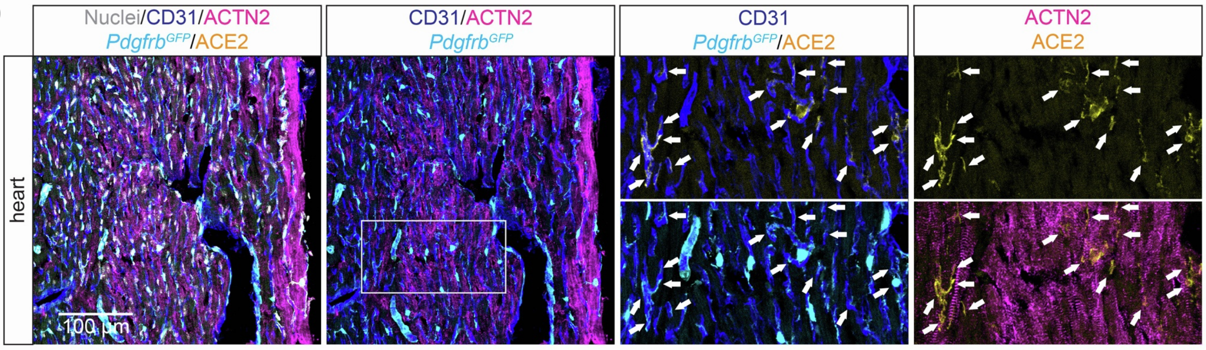

Figure S3. *Ace2* expression in mouse heart ECs is due to pericyte contamination. Related to Figure 3.

(A) *Ace2* expression in adult mouse heart scRNA-seq data enriched for stromal cells (Muhl et al., 2020). Bar plots show the expression in individual cells for *Ace2, the* VSMC marker *Acta2*, and the fibroblast maker *Pdgfra*. (B) Dot plot displaying the expression pattern of the top-50 transcripts enriched *Ace2*-positive versus *Ace2*-negative heart ECs. (C) Statistics of the number of cells, *Ace2*-positive cell percentage in each heart cell type. Abbreviations: EC, endothelial cells; FB, fibroblast; EDC, endocardial cells; CM, cardiomyocytes; PC, pericytes; aSMC, arterial smooth muscle cells; MAC, macrophages; ER, erythrocytes. (D) IF staining for ACE2 in adult mouse heart in combination with the indicated markers (*Pdgfrb*^{GFP} for pericytes, CD31 for the endothelium and sarcomeric alpha actinin (ACTN2) to mark cardiomyocytes). The arrows indicate ACE2-positive cells that are all in close contact with the vasculature and exhibit a positive *Pdgfrb*^{GFP} signal, indicating them as pericytes. In contrast, ACTN2-positive cardiomyocytes appear ACE2 negative. Nuclei are visualized by Hoechst 33342. The boxed area is shown magnified in the right panels. Scale bar is indicated in the figure.

Figure S4

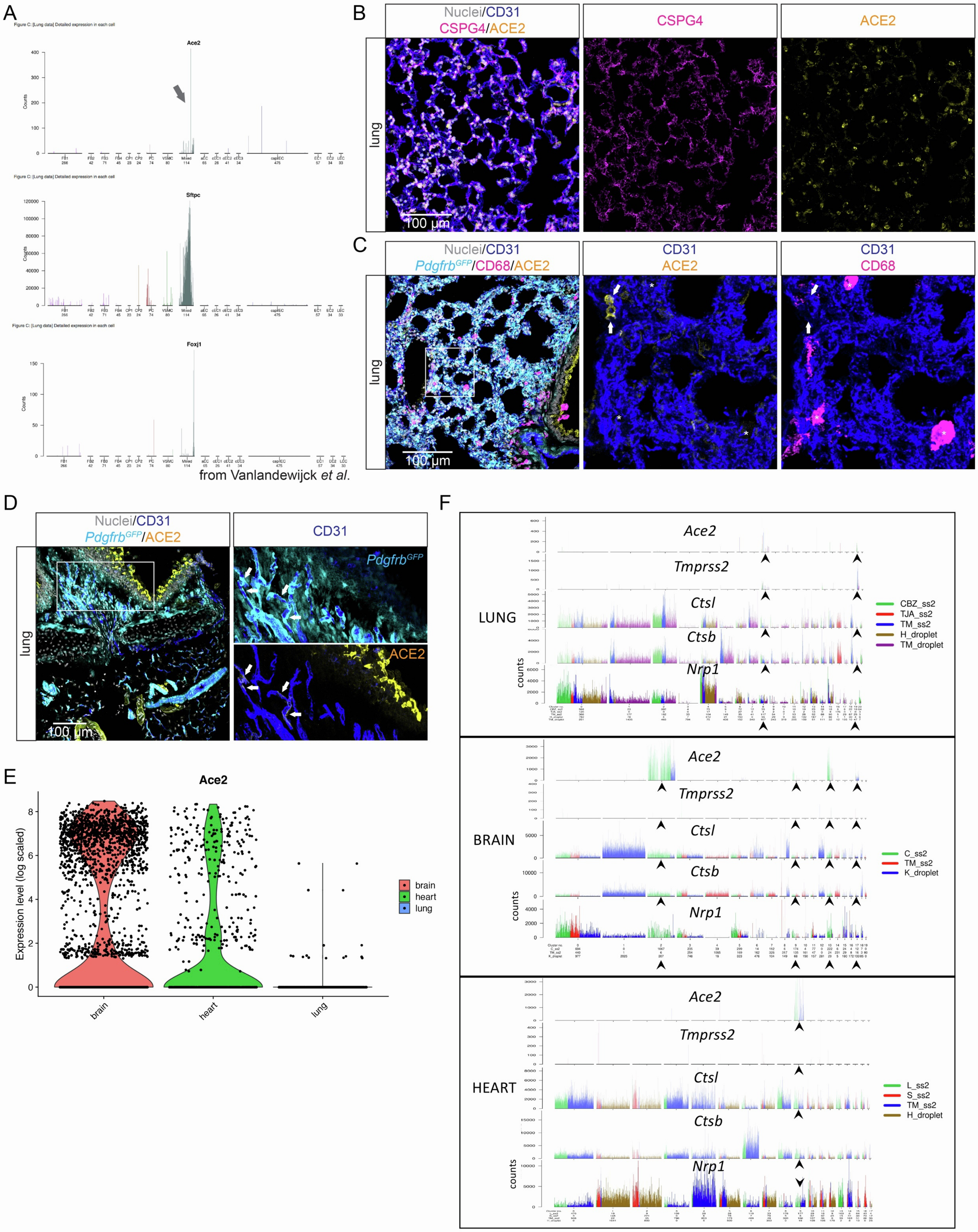
A
Figure C: [Lung data] Detailed expression in each cell
Ace2
Counts
Sftpc
Foxj1
FB1 FB2 FB3 FB4 CP1 CP2 PC VSMC Mixed aEC cEC1 cEC2 cEC3 capilEC EC1 EC2 LEC
from Vanlandewijck et al.
B
Nuclei/CD31
CSPG4/ACE2
CSPG4
ACE2
lung
100 µm
C
Nuclei/CD31
PdgfrbGFP/CD68/ACE2
CD31
ACE2
CD31
CD68
lung
100 µm
D
Nuclei/CD31
PdgfrbGFP/ACE2
CD31
PdgfrbGFP
ACE2
lung
100 µm
E
Ace2
Expression level (log scaled)
brain
heart
lung
F
LUNG
Ace2
Tmprss2
Ctsl
Ctsb
Nrp1
counts
CBZ_ss2
TJA_ss2
TM_ss2
H_droplet
TM_droplet
BRAIN
C_ss2
TM_ss2
K_droplet
HEART
L_ss2
S_ss2
TM_ss2
H_droplet

Figure S4. ACE2 protein expression in the adult mouse lung.

Related to Figure 4.

(A) *Ace2* expression in adult mouse lung scRNA-seq data enriched for ECs and pericytes (Vanlandewijck et al., 2018). Bar plots show the expression in individual cells for *Ace2*, the AT-II cell marker *Sftpc*, and the multiciliated cell marker *Foxj1*. (B-D) Confocal microscopy images of sections from adult mouse lung stained with the indicated antibodies (ACE2, CD31 for endothelium, CSPG4 (aka NG2) for mural cells, CD68 for macrophages) and the transgenic reporter *Pdgfrb*^{GFP} for mural cells. ACE2 IF signal is only detected in bronchial epithelium (strong) and AT-II cells (weak). Note the absence of ACE2 IF signal in ECs, in mural cells in the alveolar region and in CD68-positive macrophages (asterisk in C). (D) IF staining showing ACE2-positive pericytes (arrows) only at the capillary bed of large, primary bronchi. Nuclei are visualized by DAPI or Hoechst 33342. Scale bars as indicated in the figure. (E) Violin plot of the *Ace2* mRNA expression levels in pericytes from brain, heart and lung, as indicated. The Y-axis shows the log scaled normalized expression counts. The individual dots represent single cells, and the violin shape shows the distribution density. (F) Expression of *Ace2* and SARS-CoV-2 spike protein processing proteases – *Tmprss2*, *Ctsl* or *Ctsb* – as well as *Nrp1* across meta-analysis scRNAseq datasets from the lung (upper), brain (middle) or heart (lower) presented as bar plots. Each bar represents a single cell and is colored according to the indicated data source (see Supplementary Information - Methods). Cluster annotations have been described in previous figures. Arrows indicate *Ace2*-positive cell clusters: in the lung AT-II cells (left) and multiciliated cells (right), in the brain from left to right, pericytes, arteriolar VSMCs, venous VSMCs and pericyte-contaminated ECs, in the heart, pericytes.

Figure S5

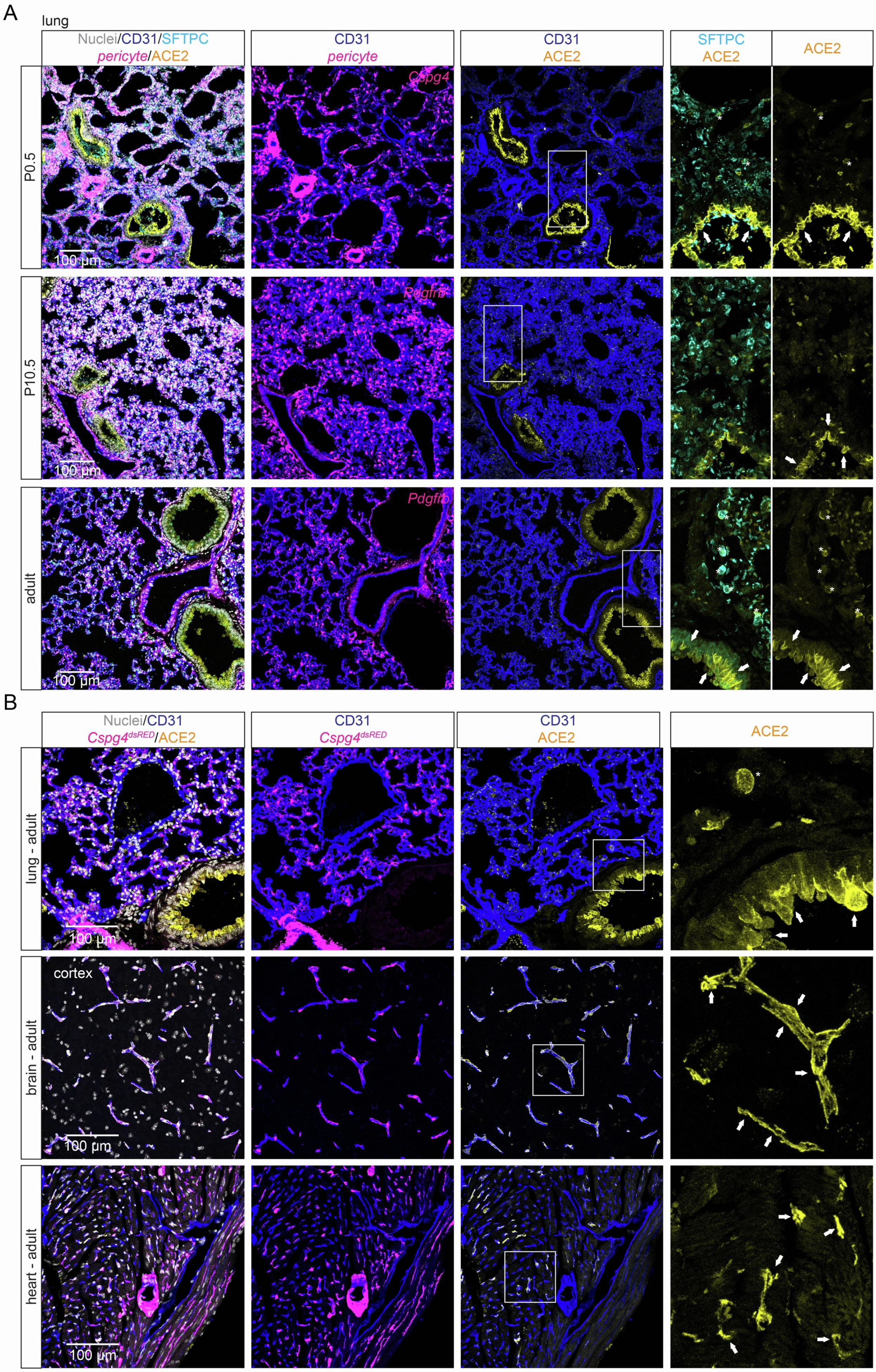

A
lung
Nuclei/CD31/SFTPC
pericyte/ACE2
CD31
pericyte
CD31
ACE2
SFTPC
ACE2
ACE2
P0.5
Cspg4
100 µm
P10.5
Pdgfrb
100 µm
adult
Pdgfrb
100 µm
B
Nuclei/CD31
Cspg4dsRED/ACE2
CD31
Cspg4dsRED
CD31
ACE2
ACE2
lung - adult
100 µm
brain - adult
cortex
100 µm
heart - adult
100 µm

Figure S5. ACE2 expression in the developing and adult lung, brain and heart. Related to Figure 5.

(A) IF staining for indicated proteins in mouse lung from P0.5, P10.5 and adult time-points. Note the sporadic expression of ACE2 in AT-II cells already visible at P0.5. Arrows indicate ACE2-positive bronchial epithelial cells, asterisks indicate ACE2-positice AT-II cells (also SFTPC-positive). Boxed areas are shown magnified in the right panel. (B) IF staining for the indicated proteins in the adult mouse lung, brain and heart. Note the strong expression of ACE2 in the bronchial epithelial cells (arrows, upper panel) and AT-II cells (asterisk, upper panel), or pericytes of the brain (middle panel) and heart (lower panel). Note the lack of ACE2 expression in cardiomyocytes in the heart. Nuclei are visualized by Hoechst 33342. Scale bars as indicated in the figure.

Figure S6

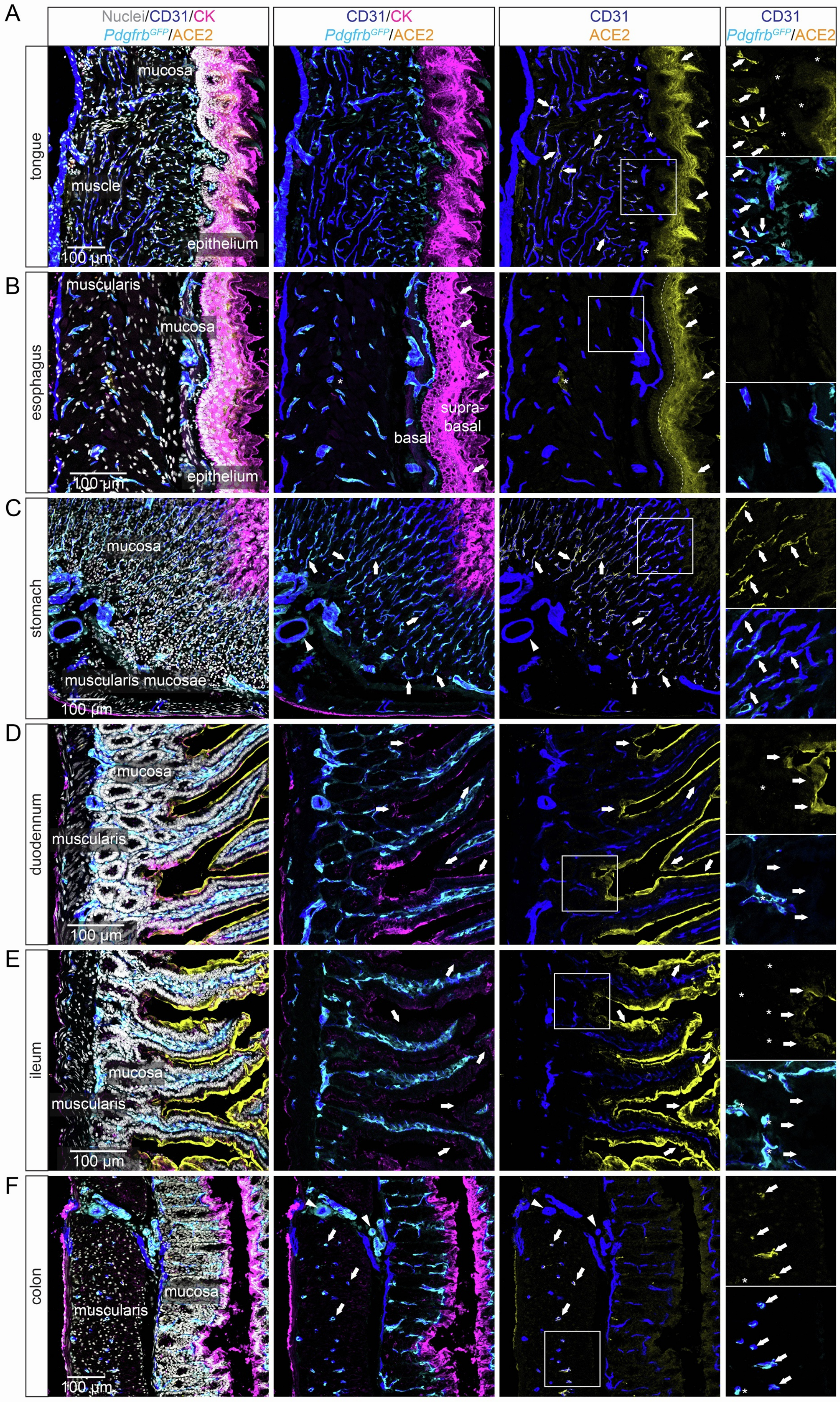

A
B
C
D
E
F
Nuclei/CD31/CK
PdgfrbGFP/ACE2
CD31/CK
PdgfrbGFP/ACE2
CD31
ACE2
CD31
PdgfrbGFP/ACE2
tongue
mucosa
muscle
epithelium
100 µm
esophagus
muscularis
mucosa
epithelium
basal
supra-
basal
100 µm
stomach
mucosa
muscularis mucosae
100 µm
duodenum
mucosa
muscularis
100 µm
ileum
mucosa
muscularis
100 µm
colon
mucosa
muscularis
100 µm

Figure S6. ACE2 protein expression in the adult gastrointestinal tract.

Related to Figure 7.

IF detection of ACE2 in adult mouse gastrointestinal (GI) tract in combination with the indicated markers (*Pdgfrb*^{GFP} for pericytes, CD31 for endothelial cells and cytokeratin (CK) is used for epithelial structures). (A) IF staining of adult mouse tongue, showing strong ACE2 expression, highlighted by arrows, in the surface epithelium as well as pericytes of the tongue muscle. Asterisks indicate ACE2-negative pericytes of the mucosal vasculature. (B) IF staining of adult mouse esophagus, showing strong ACE2 expression in the surface epithelium, highlighted by the arrows. The basal cell layer exhibits a weaker ACE2 signal, compared to the suprabasal cell layer. The border between basal and suprabasal cell layers is indicated by the dashed line. The asterisk indicates an ACE2-positive nerve. (C) IF staining of adult mouse stomach, showing ACE2 expression in pericytes of the mucosal vasculature, highlighted by arrows. (D) IF staining of adult mouse duodenum. The arrows highlight the ACE2-positive surface epithelium of the villi and crypts. The asterisk indicates ACE2 negative pericytes of the mucosal vasculature. (E) IF staining of adult mouse ileum. The arrows highlight the ACE2-positive surface epithelium of the villi. In the magnified box, arrows indicate the, compared to the duodenum, low signal of ACE2 at the crypt of the ileum and asterisks indicate ACE2-negative pericytes of the mucosa vasculature. (F) IF staining of adult mouse colon. The arrows indicate ACE2-positive pericytes of the muscularis vasculature. Not all pericytes of the muscularis vascular system are positive and an ACE2-negative pericyte is marked by the asterisk. Arrowheads highlight ACE2-negative arterial VSMC. Boxed areas are shown magnified in the right panel. Nuclei are visualized by Hoechst 33342. Scale bars are indicated in the figure.

Figure S7

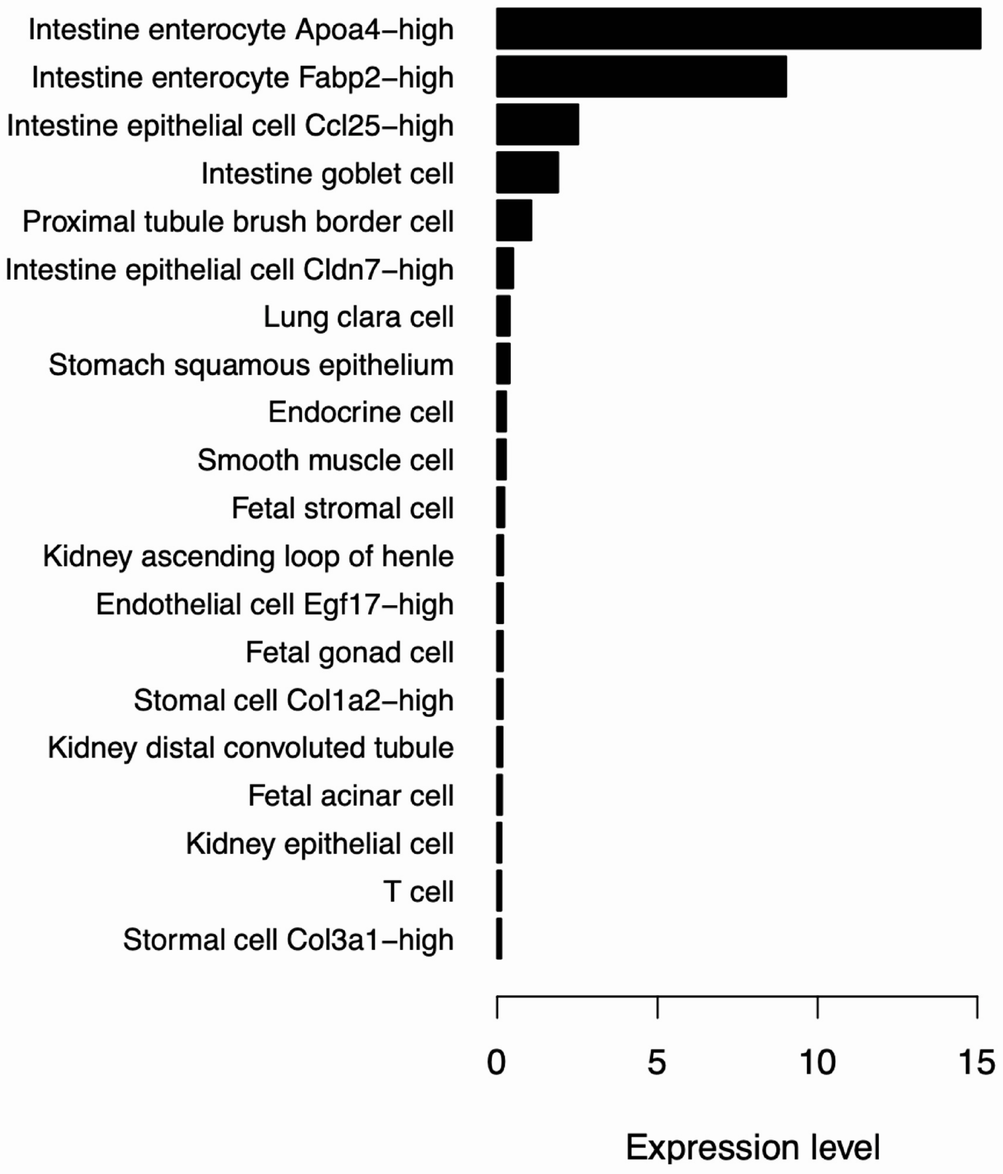

Figure S7. *Ace2* expression in the adult mouse gastrointestinal tract. Related to Figure 7.

Bar plot of the *Ace2* mRNA expression levels in main mouse cell types (data obtained from the Mouse Cell Atlas (http://bis.zju.edu.cn/MCA/index.html). The X-axis shows the average normalized expression level.

Table S1. List of used antibodies.

Primary antibody	Dilutions	Supplier	Catalog number
PECAM1, CD31	1:200	R & D Systems	AF3628
PECAM1, CD31	1:100	BD Pharmingen	553370
PECAM1, CD31	1:50	Abcam	ab28364
ANPEP	1:100	Bio-Rad	MCA2183EL
COLIV	1:100	Bio-Rad	2150-1470
ACTA2 (aSMA)-Alexa Fluor 647	1:200	Santa Cruz Biotechnology, Inc.	sc-32251
ACTA2 (aSMA)-FITC	1:200	Sigma	F3777
ACTA2 (aSMA)-Cy3	1:500	Sigma	C6198
PDGFRb	1:100	eBiosciences	553847
ACE2	1:100	R & D Systems	AF3437
SFTPC	1:100	Abcam	ab40879
CSPG4 (NG2)	1:200	Millipore	AB5320
CD68	1:200	BioLegend	137001
GLUCAGON (GCG)	1:200	Millipore	AB932
NGFR	1:200	Abcam	ab52987
THYROSIN HYDROXYLASE (TH)	1:200	Pel Freez Biologicals	P40101-0
SARCOMERIC ALPHA-ACTININ (ACTN2)	1:300	Abcam	ab137346
CYTOKERATIN (CK)	1:200	DAKO	Z0622

Supplemental Experimental Procedures:

Fixation, sectioning and antibody incubations:

Vibratome-sections: Brains were removed and post-fixed in 4% buffered formaldehyde for 4h at 4°C. Sagittal and coronal vibratome sections (50-75 µm) were incubated in blocking/permeabilization solution (1% bovine serum albumin, 2.5% donkey serum, 0.5% Triton X-100 in PBS) overnight at 4°C, followed by incubation in primary antibody solution for two nights at 4°C, and subsequently in secondary antibody (Jackson ImmunoResearch and Invitrogen) solution, overnight at 4°C. A list of the used primary antibodies is presented in Table S1. Sections were mounted in ProLong Gold Antifade mounting medium (cat. #P36930, Life Technologies). Micrographs were acquired with a Leica TCS SP8 confocal microscope (Leica Microsystems). All confocal images are represented as maximum intensity projections and were adjusted for brightness and contrast using Fiji v1.52p and Adobe Photoshop CC 2019.

Cryo-sections: Tissues were harvested from euthanized mice without perfusion and fixed by immersion in 4% formaldehyde for 4-12h at 4°C, followed by immersion in 20% sucrose/PBS solution for at least 24h at 4°C. Thereafter, tissues were embedded for cryo-sectioning and sectioned on a CryoStat NX70 (ThermoFisher Scientific) to 14 or 30 µm thick sections collected on SuperFrost Plus glass slides (Metzler Gläser) and stored at -80°C until usage. Sections were allowed to thaw at RT and thereafter blocked for > 60 min at RT with blocking-buffer (serum-free protein blocking solution, DAKO), supplemented with 0.2% Triton X-100 (Sigma Aldrich), followed by sequential incubation with primary antibodies (overnight at 4°C) (Table S1) and corresponding fluorescently conjugated secondary antibodies (1h at RT) together with 10 µg/ml *Hoechst 33342* (trihydrochloride, trihydrate, ThermoFisher Scientific). Sections were mounted with ProLong Gold Antifade mounting medium, and micrographs acquired and graphically handled as described above.
