# Supplementary material for: The SARS-CoV-2 receptor ACE2 is expressed in mouse pericytes but not endothelial cells: Implications for COVID-19 vascular research
Source: Stem Cell Reports. 2022 Apr 21;17(5):1089–104. doi: 10.1016/j.stemcr.2022.03.016 (PMC9022216; doi:10.1016/j.stemcr.2022.03.016)
Supplement: Document S2. Article plus supplemental information [file mmc2.pdf]

Stem Cell Reports
Article
OPEN ACCESS

The SARS-CoV-2 receptor ACE2 is expressed in mouse pericytes but not endothelial cells: Implications for COVID-19 vascular research

Lars Muhl,[1,13] Liqun He,[2,3,13] Ying Sun,[2,13] Maarja Andaloussi Mäe,[2] Riikka Pietilä,[2] Jianping Liu,[1] Guillem Genové,[1] Lei Zhang,[4] Yuan Xie,[4] Stefanos Leptidis,[1] Giuseppe Mocci,[1] Simon Stritt,[2] Ahmed Osman,[5] Andrey Anisimov,[6] Karthik Amudhala Hemanthakumar,[6] Markus Räsänen,[6] Emil M. Hansson,[1] Johan Björkegren,[1,7] Michael Vanlandewijck,[1,2] Klas Blomgren,[5,8] Taija Mäkinen,[2] Xiao-Rong Peng,[9] Yizhou Hu,[10] Patrik Ernfors,[10] Thomas D. Arnold,[11] Kari Alitalo,[6] Urban Lendahl,[12,*] and Christer Betsholtz[1,2,*]

[1]Department of Medicine, Huddinge, Karolinska Institutet, Solna, Sweden
[2]Department of Immunology, Genetics, and Pathology, Rudbeck Laboratory, Uppsala University, Uppsala, Sweden
[3]Department of Neurosurgery, Tianjin Medical University General Hospital, Tianjin Neurological Institute, Key Laboratory of Post-Neuro-injury Neuro-Repair and Regeneration in Central Nervous System, Ministry of Education and Tianjin City, Tianjin 300052, China
[4]Key Laboratory of Ministry of Education for Medicinal Plant Resource and Natural Pharmaceutical Chemistry, National Engineering Laboratory for Resource Developing of Endangered Chinese Crude Drugs in Northwest of China, College of Life Sciences, Shaanxi Normal University, Xi'an China
[5]Department of Women's and Children's Health, Karolinska Institutet, Solna, Sweden
[6]Wihuri Research Institute and Translational Cancer Medicine Program, Biomedicum Helsinki, University of Helsinki, Helsinki, Finland
[7]Institute of Genomics and Multiscale Biology, Department of Genetics and Genomic Sciences, Icahn School of Medicine at Mount Sinai, New York, NY 10029, USA
[8]Department of Pediatric Oncology, Karolinska University Hospital, Stockholm Sweden
[9]Cardiovascular, Renal and Metabolism, AstraZeneca BioPharmaceutical R&D, Gothenburg, Sweden
[10]Department of Medical Biochemistry and Biophysics, Karolinska Institutet, Solna, Sweden
[11]Department of Pediatrics, University of California San Francisco, San Francisco, CA 94143, USA
[12]Department of Cell and Molecular Biology, Karolinska Institutet, Solna, Sweden
[13]These authors contributed equally
*Correspondence: urban.lendahl@ki.se (U.L.), christer.betsholtz@igp.uu.se or christer.betsholtz@ki.se (C.B.)
https://doi.org/10.1016/j.stemcr.2022.03.016

SUMMARY

Humanized mouse models and mouse-adapted SARS-CoV-2 virus are increasingly used to study COVID-19 pathogenesis, so it is important to learn where the SARS-CoV-2 receptor ACE2 is expressed. Here we mapped ACE2 expression during mouse postnatal development and in adulthood. Pericytes in the CNS, heart, and pancreas express ACE2 strongly, as do perineurial and adrenal fibroblasts, whereas endothelial cells do not at any location analyzed. In a number of other organs, pericytes do not express ACE2, including in the lung where ACE2 instead is expressed in bronchial epithelium and alveolar type II cells. The onset of ACE2 expression is organ specific: in bronchial epithelium already at birth, in brain pericytes before, and in heart pericytes after postnatal day 10.5. Establishing the vascular localization of ACE2 expression is central to correctly interpret data from modeling COVID-19 in the mouse and may shed light on the cause of vascular COVID-19 complications.

INTRODUCTION

COVID-19 is caused by the severe acute respiratory syndrome coronavirus 2 (SARS-CoV-2). SARS-CoV-2 infects cells through binding of its spike (S) protein to cell surface receptors (angiotensin converting enzyme 2 [ACE2]), followed by S protein cleavage (priming) by the transmembrane serine protease 2 (TMPRSS2) or cathepsins L or B (CTSL and CTSB), fusion of viral and cellular membranes, and viral RNA entry into the cytoplasm (Hoffmann et al., 2020). The initial range of COVID-19 symptoms is explained by expression of ACE2 and TMPRSS2 or CTSL/B in several types of epithelial cells in the nasal cavities, lung, gastrointestinal tract, and eye (Muus et al., 2021; Sungnak et al., 2020; Ziegler et al., 2020). Later in the disease course, some COVID-19 patients however develop additional symptoms, including systemic inflammation, venous and arterial thrombosis with pulmonary embolism, myocardial infarction and stroke, acute kidney injury, and neurological manifestations (for review see Burkert et al., 2021).

The pathophysiological basis of the vascular problems in COVID-19 remains poorly understood, but endothelial injury appears to play an important role. Thrombosis as well as elevated circulating levels of coagulation-promoting factors such as von Willebrand factor (VWF) and pro-inflammatory cyto/chemokines including angiopoietin-2 (Ackermann et al., 2020; Dupont et al., 2021; McGonagle et al., 2021) suggest that severe COVID-19 in part should be considered an endothelial disease (Libby and Lüscher, 2020).

The literature is however conflicting as to whether endothelial cells (ECs) are directly infected by SARS-CoV-2 or not. Endothelial ACE2 expression has been reported based on immunodetection (Hamming et al., 2004; Lovren et al., 2008; Sluimer et al., 2008) or single-cell RNA sequencing (scRNA-seq) studies (Muus et al., 2021), and the presence of SARS-CoV-2 virus particles in ECs in

This is an open access article under the CC BY license (http://creativecommons.org/licenses/by/4.0/).

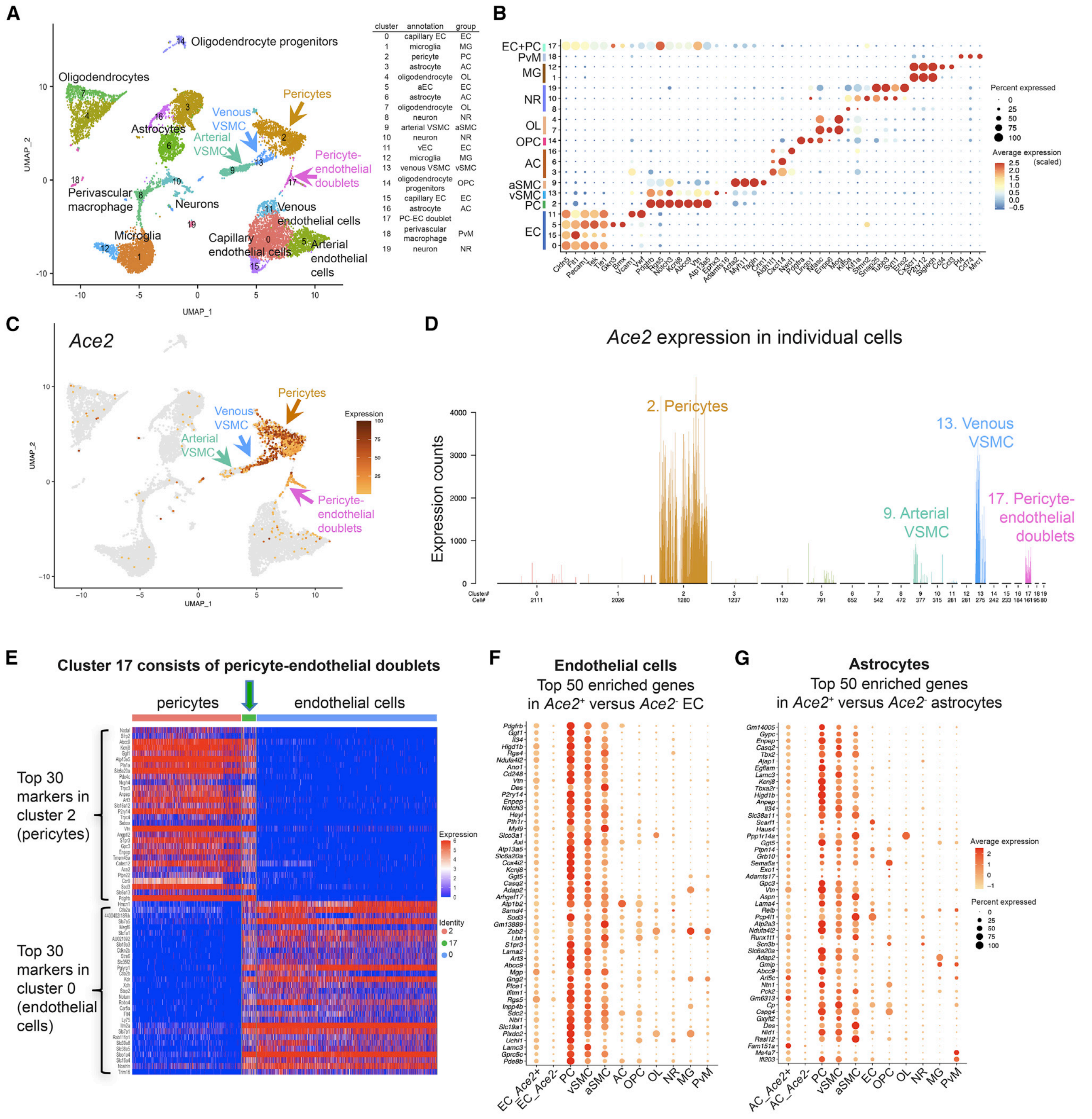

Figure 1. *Ace2* expression in adult mouse brain

(A) UMAP display of integrated mouse brain scRNA-seq data. Coloring is based on cluster assignment, and cellular annotations are based on canonical marker expression available at http://betsholtzlab.org/Publications/BrainIntegration/search.html.

(B) Dot plot showing the expression of marker genes for each cluster.

(C) The same UMAP as in (A) with *Ace2* expression overlay (dark color represents higher expression, and gray color represents *Ace2*-negative cells).

(D) Bar plot of the normalized expression levels of *Ace2* in each cluster. Cell type annotations for each cluster are indicated. Individual bars represent single cells and are colored according to cluster assignment together with cell number contributions below the x axis. Arrows of different colors in (A) and (C) indicate the *Ace2*-positive clusters in the UMAP corresponding to the bar plot displays.

(legend continued on next page)

COVID-19 patients has been proposed (Ackermann et al., 2020; Varga et al., 2020), although the data interpretation has also been questioned (Goldsmith et al., 2020). In contrast to these observations, we failed to both detect endothelial *ACE2* expression in human transcriptomic data and infect human ECs *in vitro* (McCracken et al., 2021).

If COVID-19-associated endotheliopathy is not caused by direct infection of ECs by SARS-CoV-2, EC damage may result from their vicinity to other infected cell types, including pulmonary epithelial cells or non-endothelial vascular or perivascular cells (Nicosia et al., 2021). An indirect effect is indeed consistent with induction of endotheliopathy-like responses by plasma from critically ill COVID-19 patients (Queisser et al., 2021; Rauch et al., 2020). Similarly, in studies with epithelial cells and ECs cultured on a chip, only epithelial cells were infected, but with subsequent damage evident also in the ECs (Deinhardt-Emmer et al., 2021). Among potential non-epithelial targets of SARS-CoV-2 infection, pericytes, which are microvascular mural cells in direct contact with the endothelium (Lendahl et al., 2019), have been suggested to express ACE2 (Chen et al., 2020; He et al., 2016, 2018; McCracken et al., 2021; Nicin et al., 2020; Vanlandewijck et al., 2018).

Progress in understanding the cause of the COVID-19 vascular problems is aided by research in experimental animal models, including humanized ACE2 transgenic mice, as standard laboratory mice cannot be infected by current pandemic strains of SARS-CoV-2 (Jiang et al., 2020; McCray et al., 2007; Sun et al., 2020). Through these models it may be possible to address how endotheliopathy occurs, but a prerequisite for such studies is to first establish the specific expression patterns of ACE2 and other proteins that facilitate cellular entry of SARS-CoV-2 in the mouse. In this report, we address the organotypicity of ACE2 expression and demonstrate that pericytes in several organs express ACE2, while ECs do not. These data are relevant for disease modeling of COVID-19 in the mouse and to shed light on possible primary entry points for SARS-CoV-2 in the vasculature.

RESULTS

Single-cell RNA sequencing shows that *Ace2* is expressed in brain pericytes but not in endothelial cells

We first assessed the expression and distribution of *Ace2* mRNA in the adult mouse brain. Our previous reports (He et al., 2018; Vanlandewijck et al., 2018) showed that *Ace2* is strongly enriched in brain pericytes and venous vascular smooth muscle cells (VSMCs), qualifying it among the top 15 specific markers for these cells (Figures S1A and S1B and http://betsholtzlab.org/VascularSingleCells/database.html). At lower levels, *Ace2* was also found in arterial/arteriolar VSMC cell clusters (Figure S1C), but not in *Cnn1*-positive arterial VSMC (Figures S1C and S1D). In addition, we noticed *Ace2* expression in a subset of cells annotated as brain fibroblasts (Figure S1C), but their specific expression of numerous pericyte markers, including *Kcnj8*, suggests that these fibroblasts may be related to, or contaminated by, pericytes (Figure S1C).

Analysis of an integrated mouse brain scRNA-seq dataset, combining two published (He et al., 2018; Schaum et al., 2018; Vanlandewijck et al., 2018) and one unpublished brain vasculature-focused scRNA-seq datasets (Figures 1A and 1B; see experimental procedures for details on acquisition of the datasets; available for gene-by-gene search at http://betsholtzlab.org/Publications/BrainIntegration/search.html), confirmed that *Ace2* mRNA localized primarily to pericytes and venous VSMCs (Figures 1C and 1D). In addition, we found *Ace2* transcripts in a cluster annotated as pericyte-endothelial cell doublets (Figures 1C and 1D), based on the proportional expression of both endothelial and pericyte transcripts (Figure 1E). Exploring the unbiased assembly of mouse brain single-cell transcriptomes from Zeisel et al. (2018) (http://mousebrain.org/genesearch.html) confirmed *Ace2* expression in four cell clusters representing pericytes and pericyte-endothelial doublets, but importantly it failed to identify *Ace2* transcripts at significant levels in any of ≈250 other brain cell types (Figure S1F and S1G).

Brain endothelial single-cell transcriptomes were largely devoid of *Ace2* mRNA (Figures 1C, D, and S1B). The 1.4% (48 of 3,416) of the ECs displaying *Ace2* mRNA sequence reads (as compared to ≈57% of the pericytes; Figure S1H), however, contained many canonical brain pericyte markers, including *Pdgfrb* (platelet-derived growth factor receptor beta), *Cd248* (endosialin), *Vtn* (vitronectin), *Des* (desmin), *Notch3*, and *Kcnj8* (Figure 1F). The expression of the top 50 *Ace2*-correlated genes in the ECs was generally high in mural cells (pericytes and VSMCs) but low in other cell types, including astrocytes, oligodendrocytes, microglia, and neurons (Figures 1F and S1I).

A similar picture emerged for the rare *Ace2*-positive astrocytes. By assigning the top 50 differentially expressed genes correlating with the presence of *Ace2* in the 13

(E) Expression of endothelial and pericyte enriched transcripts (each top 30 genes). Note the expression of both gene-sets in cluster 17; pericyte-endothelial doublets (green arrow).
(F and G) Dot plots showing the expression of the 50 most enriched genes in *Ace2*-positive versus *Ace2*-negative ECs (F) or astrocytes (G). Note the enrichment of differentially expressed genes in mural cell clusters (PC, vSMC, aSMC).

Ace2-positive versus the 2,060 *Ace2*-negative astrocytes (Figure S1H), we again noticed the presence of several pericyte markers, including *Kcnj8, Abcc9, Higd1b, Anpep* (CD13), *Vtn*, and *Cspg4* (NG2), and that the majority of the 50 genes were expressed at corresponding levels in mural cells (Figures 1G and S1J). We conclude from these analyses that pericytes and venous VSMCs are the predominant ACE2-expressing cells in the brain, and the rare occurrence of *Ace2* transcripts in brain cells annotated as ECs or astrocytes is contributed through contamination by mural cell fragments.

Immunofluorescence analysis reveals ACE2 expression in CNS microvascular mural cells, but not in ECs or arterial VSMCs

Strong ACE2 immunofluorescence (IF) staining was observed in mural cells associated with capillaries, venules, and veins (Figures 2A, S2A, and S2B), and weakly ACE2-positive VSMCs were present at terminal arterioles in the adult mouse brain cortex (Figures 2A and S2B). In contrast, the alpha-smooth muscle actin (αSMA)-positive mural cells located around arteries and larger arterioles were ACE2-negative (Figure 2B). ACE2 IF fully decorated the pericyte outline (Figure 2C) (Ornelas et al., 2021).

Spinal cord ACE2 was detected in cells with the typical morphology and marker expression of pericytes (Figure S2B). Weaker ACE2 expression was noted also in αSMA-positive VSMCs at terminal arterioles, whereas CNN1-positive VSMCs of larger arteries were ACE2 IF-negative (Figures S2C and S2D). Retinal pericytes were strongly ACE2-positive (Figure S2E) and αSMA-positive VSMCs in small diameter retinal arterioles were weakly ACE2-positive, whereas larger diameter arterioles/arteries were ACE2-negative (Figure S2E). Choriocapillaris, the network of fenestrated capillaries located immediately behind the retinal pigment epithelium, harbored strongly ACE2-positive pericytes (Figure S2E), whereas αSMA-positive vessels feeding this capillary plexus were ACE2-negative (Figure S2E). ACE2-positive pericytes were further found in the ciliary body (Figure S2E). Pericytes located in the extra-ocular skeletal muscle, hence residing outside of the CNS, were ACE2-negative (Figure S2F), while the surface epithelium of the conjunctiva and cornea was ACE2-positive (Figure S2E), confirming recent observations by others (Muus et al., 2021). Together, we observe ACE2 expression in CNS mural cells, while ECs were consistently negative for ACE2 IF at all locations analyzed within the CNS.

Ace2 is specifically expressed in pericytes of the heart

It was recently suggested that *ACE2* expression occurs across multiple human cardiac cell types, including cardiomyocytes, ECs, pericytes, fibroblasts, and macrophages (Chen et al., 2020; Muus et al., 2021; Nicin et al., 2020). In an adult mouse heart scRNA-seq data enriched for stromal cells (Muhl et al., 2020), we found prominent expression of *Ace2* in pericytes, but no expression in fibroblasts nor in VSMCs (Figure S3A). The integrated data from the three unpublished and one published (Schaum et al., 2018) datasets (http://betsholtzlab.org/Publications/HeartIntegration/search.html) provided comprehensive coverage of the principal cell types in the mouse heart, including cardiomyocytes, different types of ECs (blood vascular, endocardial, lymphatic), pericytes, VSMCs, and subtypes of fibroblasts and macrophages (Figures 3A and 3B). Of these, only the pericyte cluster showed distinct *Ace2* expression (Figures 3C and 3D). Rare ECs displayed low RNA-seq counts for *Ace2,* but *Ace2*-positive cells showed an enrichment of pericyte transcripts (Figures S3B and S3C). Importantly, Ace2 expression was neither observed in adult cardiomyocytes (Figures 3D, S3B, and S3D) nor in cardiomyocytes from embryonic day (E)9.5, E13.5, E15.5 or newborn (data not shown). Together, this suggests that the low levels of *Ace2* sequences found in rare cardiac ECs were contributed by contaminating pericyte-derived cellular material, similar to our observations in the brain.

Cardiac ACE2 protein IF signal was detected only in cells expressing *Pdgfrb*^{GFP} (as a marker for mural cells; Vanlandewijck et al., 2018) and with a location and morphology typical for pericytes: a round cell body and long processes adhering to the ECs (Figures 3E and 3F). In contrast to the CNS, where small arteriolar and venous mural cells were also positive, heart *Ace2* mRNA and protein was found only in capillary pericytes and not in other mural cells (Figures 3C–3E and S3D). Furthermore, cardiac pericyte ACE2 expression was heterogeneous, varying from strongly positive to negative (Figure 3F). Our analysis establishes a subset of the pericytes as the major site of *Ace2* expression in the adult heart, and ACE2 expression was consistently undetectable in mouse cardiac ECs as well as in cardiomyocytes and other cardiac cell types.

In the lung, airway epithelial cells form the major *Ace2* expression site

Lung epithelial cells are known primary targets for SARS-CoV-2 infection, but it has recently been proposed that lung ECs may also become infected (Ackermann et al., 2020; Teuwen et al., 2020). Our previously published scRNA-seq dataset of pulmonary vascular cells (Vanlandewijck et al., 2018) did not reveal *Ace2* expression in lung vascular cells but showed a distinct *Ace2* signal in epithelial cells co-expressing markers of multiciliation or surfactant secretion (Figure S4A (http://betsholtzlab.org/VascularSingleCells/database.html), pointing to ciliated bronchial epithelial cells and alveolar type II (AT-II) cells as sites of *Ace2* expression. To more precisely define the

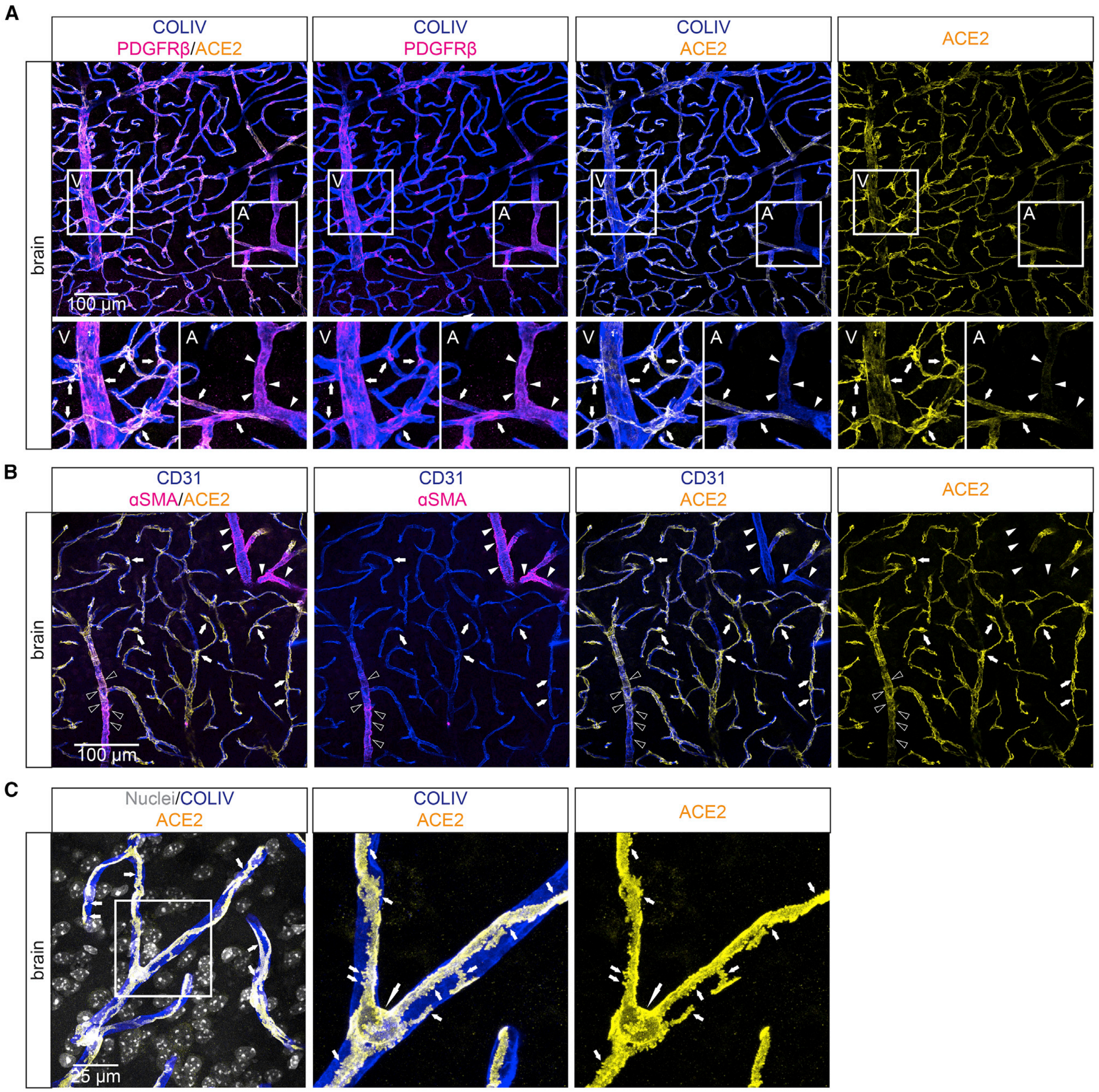

Figure 2. ACE2 IF in adult mouse brain

(A–C) IF detection of ACE2 in adult mouse brain in combination with indicated markers. CD31 and collagen type IV (COLIV) are used as markers for the endothelium and basement membrane, respectively. PDGFRβ marks mural cells, and αSMA marks VSMCs. (A) A venule (*V*) and arteriole (*A*) are shown magnified. Arrows indicate pericytes; arrowheads indicate arterioles. Note the similar expression of ACE2 and PDGFRβ in pericytes and venous/venular VSMCs. (B) The absence of ACE2 from large arterioles (arrowheads) and weak presence at terminal arterioles (open arrowheads). (C) High magnification image showing a pericyte with soma and secondary processes. Note that ACE2 staining follows the outline of the pericyte including its secondary processes. The large arrow point at the pericyte cell soma and the small arrows at its primary processes (including peg-sockets). Nuclei are visualized by DAPI or Hoechst 33342. Scale bars are indicated in the figure.

Ace2-expressing cell type, we integrated data from three published (He et al., 2018; Schaum et al., 2018; Vanlandewijck et al., 2018) and two unpublished adult mouse lung scRNA-seq datasets (Figures 4A and 4B; available for gene-by-gene browsing at http://betsholtzlab.org/Publications/LungIntegration/search.html). The integrated data showed

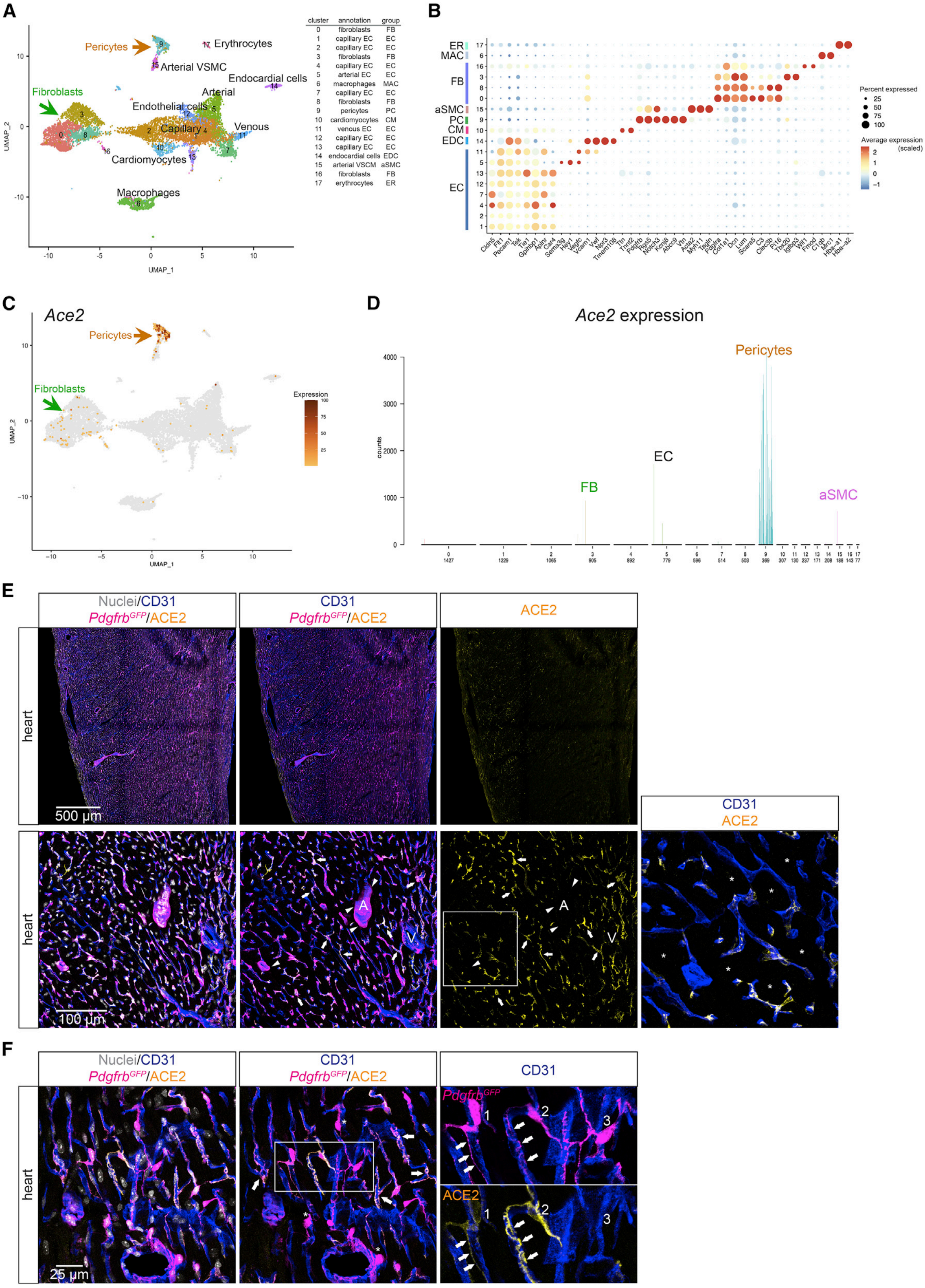

(legend on next page)

significant *Ace2* expression only in AT-II (surfactant protein C [*Sftpc*]-positive) and *Foxj1*-positive multiciliated airway epithelial cells (Figures 4B–4D and S4A). No robust *Ace2* expression was observed in any of the subtypes of vascular ECs (*Pecam1*-positive), mural cells (*Notch3*-positive), fibroblasts (*Pdgfra*-positive, *Notch3*-negative), alveolar macrophages, or other hematopoietic cells (*Ptprc*-positive) (Figure 4D).

A strong ACE2 IF signal was observed in the bronchial epithelium throughout the bronchial tree. In the alveolar region distal to the terminal bronchioles, we found ACE2 IF signal in SFTPC-positive AT-II cells (Figure 4E). We failed to detect ACE2 by IF in any endothelial populations in the lung, including alveolar capillaries and large vessels (Figures 4E and S4B–S4D). CD68-positive alveolar macrophages were also ACE2-negative (Figure S4C). Lung pericytes in the alveolar region were ACE2-negative, while we observed occasional ACE2-positive pericytes in capillaries surrounding larger bronchi (Figure S4D). Finally, pericytes in the lung showed very low levels of *Ace2* mRNA expression, in comparison with pericytes from brain and heart (Figure S4E).

To gain insights into the expression of other proteins of relevance for SARS-CoV-2 infection, we assessed the distribution of mRNAs for the S protein priming proteases *Tmprss2*, *Ctsl*, and *Ctsb* (Figure S4F). We observed *Tmprss2* co-expression with *Ace2* in AT-II cells and bronchial epithelial cells, whereas *Ctsl* and *Ctsb* were more broadly expressed (Figure S4F). In contrast, *Tpmrss2* was not expressed in brain and heart pericytes, which instead exhibited co-expression of *Ace2* with *Ctsb* and *Ctsl* (Figure S4F). Neuropilin-1 (*Nrp1*), a proposed host factor facilitating SARS-CoV-2 infection (Cantuti-Castelvetri et al., 2020; Daly et al., 2020), was again broadly expressed (Figure S4F). In summary, only *Tmprss2* showed restricted expression.

Different developmental onsets of ACE2 expression in mouse brain, heart, and lung

To establish when ACE2 expression was first observed in brain, heart, and lung, we analyzed ACE2 protein distribution in these three organs at postnatal day (P)0.5, P10.5, and in adult mice. At P0.5, we observed extensive ACE2 expression in the lung bronchial cells, occasional ACE2-expressing brain pericytes, but no ACE2-positive heart pericytes or cardiomyocytes (Figures 5A–5C). AT-II cells showed occasional expression of ACE2 already at P0.5 (Figure S5A). At P10.5, ACE2 IF was observed in CNS pericytes as well as in the bronchial epithelial cells, but only occasionally in heart pericytes (Figures 5A–5C). Together, these data indicate differential onsets of ACE2 expression in brain, heart, and lung.

ACE2 distribution in other organs

We next analyzed ACE2 distribution by IF in several other organs to obtain further insights into pericyte organotypicity. In skeletal muscle, occasional pericytes were ACE2-positive (Figure 6A), however at lower frequency compared to the heart or brain. In the adrenal gland of *Pdgfrb*^{GFP} mice (to visualize mural cells), robust ACE2 IF was observed in pericytes and perivascular cells, likely fibroblasts, located in both the cortex and the medulla (Figure 6B). In pancreas, there was ubiquitous expression of ACE2 in pericytes around the exocrine ducts and in endocrine islets. Moreover, ACE2 expression was found in glucagon-producing pancreatic α-cells (Figures 6C and 6D), the latter in keeping with previous reports (Coate et al., 2020; Fignani et al., 2020).

In the sciatic nerve, pericytes in the endoneurium were ACE2-positive, and in addition, we observed strongly ACE2 IF-positive cells surrounding nerve fascicles (Figures 7A–7C); these cells had the expected location of perineurial fibroblasts (Bunge et al., 1989). Analysis of scRNA-seq datasets from peripheral nerves (Carr et al.,

Figure 3. ACE2 expression in the adult mouse heart

(A) UMAP display of integrated mouse heart scRNA-seq data. Coloring is based on cluster assignment and cellular annotations are based on canonical marker expression available at http://betsholtzlab.org/Publications/HeartIntegration/search.html.

(B) Dot plot showing the expression of marker genes for each cluster.

(C) The same UMAP as in (A) with *Ace2* expression overlay (dark color represents higher expression, and gray color represents *Ace2*-negative cells).

(D) Bar plot of the normalized expression levels of *Ace2* in each cluster. Cell type annotations for each cluster are indicated. Individual bars represent single cells and are colored according to the cluster assignment together with cell number distribution below the x axis. Arrows of different colors in (A) and (C) indicate the *Ace2*-positive clusters in the UMAP corresponding to the bar plot displays.

(E and F) IF detection of ACE2 in adult mouse heart in combination with the indicated markers (*Pdgfrb*^{GFP} for pericytes and CD31 as a marker for the endothelium). (E) Overview image (upper panel) and detailed image including an artery (*A*) and vein (*V*) (lower panel). Note the overlapping expression of ACE2 and *Pdgfrb*^{GFP} in a proportion of pericytes and that arterial and venous VSMCs are ACE2-negative. In the high-magnification inset, ACE2-negative cardiomyocytes are shown (asterisks) along with ACE2-positive pericytes. (F) High magnification image that shows an example of three neighboring pericytes with different levels of ACE2 expression. Note also the different subcellular distribution of cell membrane-associated ACE2 and cytoplasmic GFP expressed from the *Pdgfrb* promoter (*Pdgfrb*^{GFP}).

Nuclei are visualized by DAPI or Hoechst 33342. Scale bars are indicated in the figure.

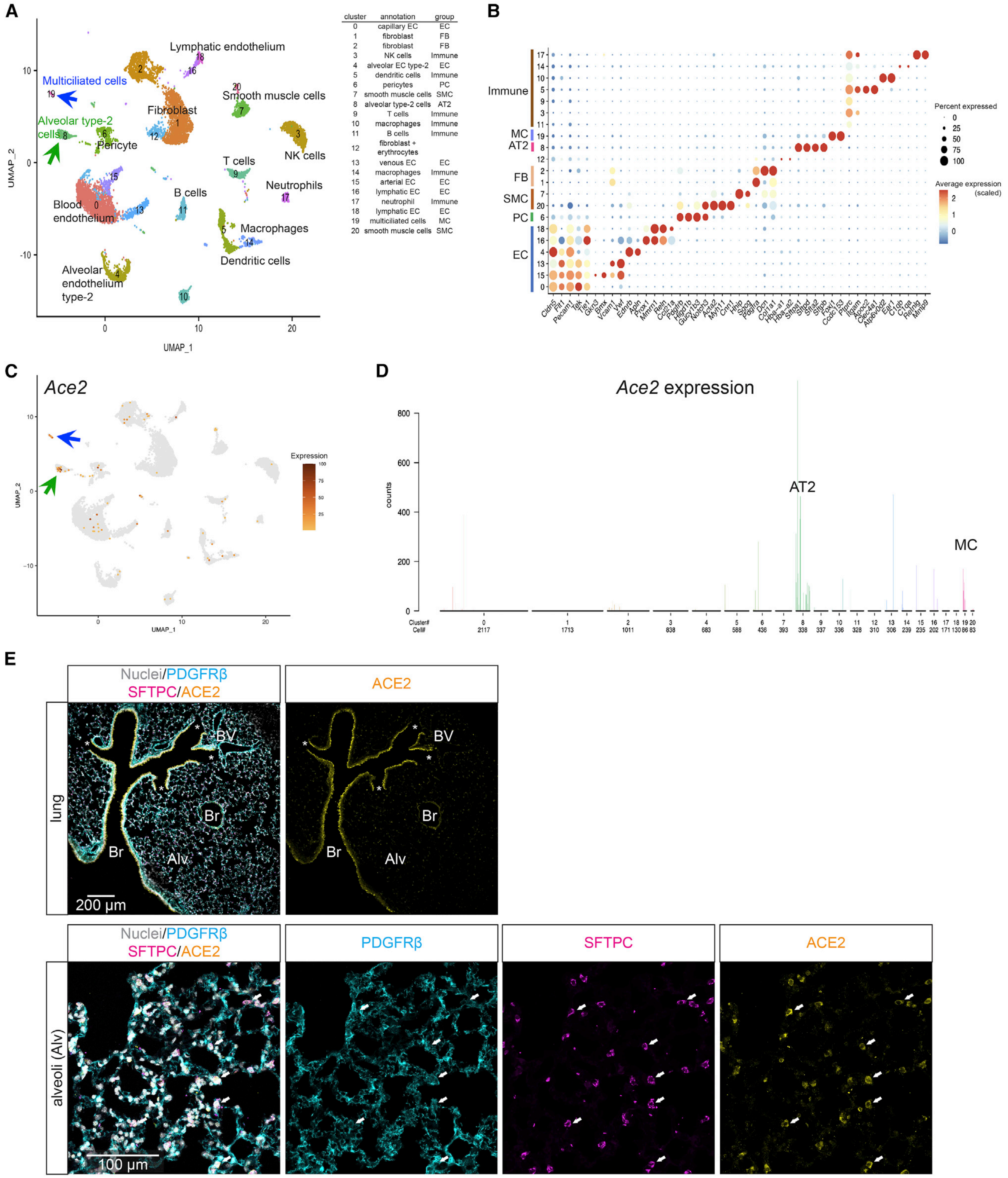

Figure 4. ACE2 expression in adult mouse lung

(A) UMAP visualization of the integrated scRNA-seq data from mouse lung. Annotations were based on the expression of canonical markers for each indicated cell type available at http://betsholtzlab.org/Publications/LungIntegration/search.html.

(B) Dot plot showing the expression of marker genes for each cluster.

(legend continued on next page)

2019; Gerber et al., 2021) confirmed *Ace2* expression in adult perineurial fibroblasts (Figure 7D). ACE2-positive perineurial fibroblasts were also observed in the lung and skeletal muscle (Figures 7E and 7F). In none of these organs did we observe ACE2 expression in ECs. Finally, we assessed ACE2 distribution by IF in the GI tract (Figures S6A–S6F). ACE2 expression was observed in pericytes in tongue muscle, stomach mucosa, and colon muscularis, while we did not find any ACE2 expression in pericytes from esophagus, duodenum, or ileum. ACE2 expression was observed in epithelial cells in tongue, esophagus, duodenum, and ileum (Figures S6A–S6F). At the mRNA level, intestinal enterocytes showed high levels of *Ace2* expression (Figure S7).

DISCUSSION

A bone of contention in recent discussions is whether COVID-19-associated endothelial injury is caused by direct virus infection of ECs or is a secondary consequence of infection of neighboring cells. A number of studies favor ACE2 expression by ECs (Ackermann et al., 2020; Lovren et al., 2008; Muus et al., 2021; Varga et al., 2020), whereas other studies advocate pericytes as a major site of ACE2-expression (Chen et al., 2020; He et al., 2016, 2018; Nicin et al., 2020; Vanlandewijck et al., 2018). In line with the latter notion, a recent study (McCracken et al., 2021) reported that the low-level *ACE2* mRNA expression observed in human ECs (Muus et al., 2021) is likely caused by pericyte contamination. Furthermore, infection of pericytes may underlie the neuropathology observed in COVID-19 patients (Bocci et al., 2021).

The data presented here show that microvascular mural cells are the predominant site of ACE2 expression in mouse brain and heart vasculature. ACE2 mRNA and protein expression was confined to pericytes, venous VSMCs, and *Cnn1*-negative arteriolar VSMCs in the CNS and to pericytes in the heart. ACE2-expressing pericytes were also observed in tongue, stomach, and colon. However, in none of these organs were ECs found to express ACE2. ACE2 is however not a universal pericyte protein. In the heart about half of the pericytes were ACE2-positive, and in the lung, there were a few ACE2-positive pericytes in capillaries surrounding large airways, while lung alveolar capillary pericytes were ACE2-negative. Similarly, ACE2-negative pericytes were observed in the duodenum and ilium.

The observation that pericytes constitute the principal vascular, albeit organotypically, ACE2-expressing cells in both mice and humans (Bocci et al., 2021; McCracken et al., 2021) indicates an evolutionary conservation of ACE2 expression in mural cells. ACE2 expression in pericytes may also have bearings on why conditions such as diabetes and obesity are risk factors for COVID-19 (Apicella et al., 2020). Common to these conditions is a dysfunctional leaky vasculature (Lindenmeyer et al., 2007; Singh et al., 2008). Pericytes are normally located behind the endothelial lining, protected from direct contact with the blood stream, and it thus appears unlikely that, in a healthy sealed vasculature, SARS-CoV-2 virus circulating in the blood stream would be able to be transported through the endothelial lining via *trans*- or paracellular routes to reach the pericytes (Claesson-Welsh, 2015). However, in a leaky vasculature, the SARS-CoV-2 virus may get access to and infect pericytes, possibly rendering them dysfunctional, leading to activation of pro-inflammatory and pro-thrombotic responses in neighboring ECs. Indeed, in a recent analysis of the endothelial reaction to pericyte loss, upregulation of several pro-inflammatory mediators, including angiopoietin-2 and VWF, was found (Andaloussi Mäe et al., 2020).

In addition to pericytes, we identify several other cell types expressing ACE2. In the lung, the major sites of ACE2 expression were instead the AT-II cells and *Foxj1*-positive multiciliated bronchial epithelial cells, in keeping with a previous report (Ziegler et al., 2020). Perineurial fibroblasts were also ACE2-positive, which is interesting in the light of the neurological manifestations of COVID-19, as the perineurial fibroblasts form a metabolically active permeability barrier and provide structural integrity to the nerve fascicle. In the GI tract, epithelial cells in the tongue, esophagus, duodenum, and ilium were ACE2-positive, which may be of interest in relation to the gastrointestinal manifestations of COVID-19 (Guo et al., 2021) and to

(C) The same UMAP as in (A) with *Ace2* expression overlay (dark color represents higher expression, and gray color represents *Ace2*-negative cells). For (A) and (C), green arrows point at the AT-II cell cluster; blue arrows point at the multiciliated cell cluster.
(D) Bar plot of the normalized expression levels of *Ace2* in each cluster. Cell type annotations for each cluster are indicated. Individual bars represent single cells and are colored according to the cluster assignment together with cell number distribution below the x axis.
(E) IF staining for indicated proteins in adult mouse lung. A prominent ACE2 signal is observed in bronchial epithelium. Asterisks mark end of terminal bronchioles. The lower panels show the same microscopic field of the alveolar region with different labels visualized. Arrows provide landmarks and point at SFTPC-positive AT-II cells. Note the overlap between ACE2 and SFTPC in the alveolar region and lack of ACE2 staining of pericytes (labeled by PDGFRβ). No ACE2 IF signal was observed in ECs. Nuclei are visualized by DAPI or Hoechst 33342. Br: bronchi, BV: blood vessel. Alv: alveolar region Scale bars are indicated in the figure.

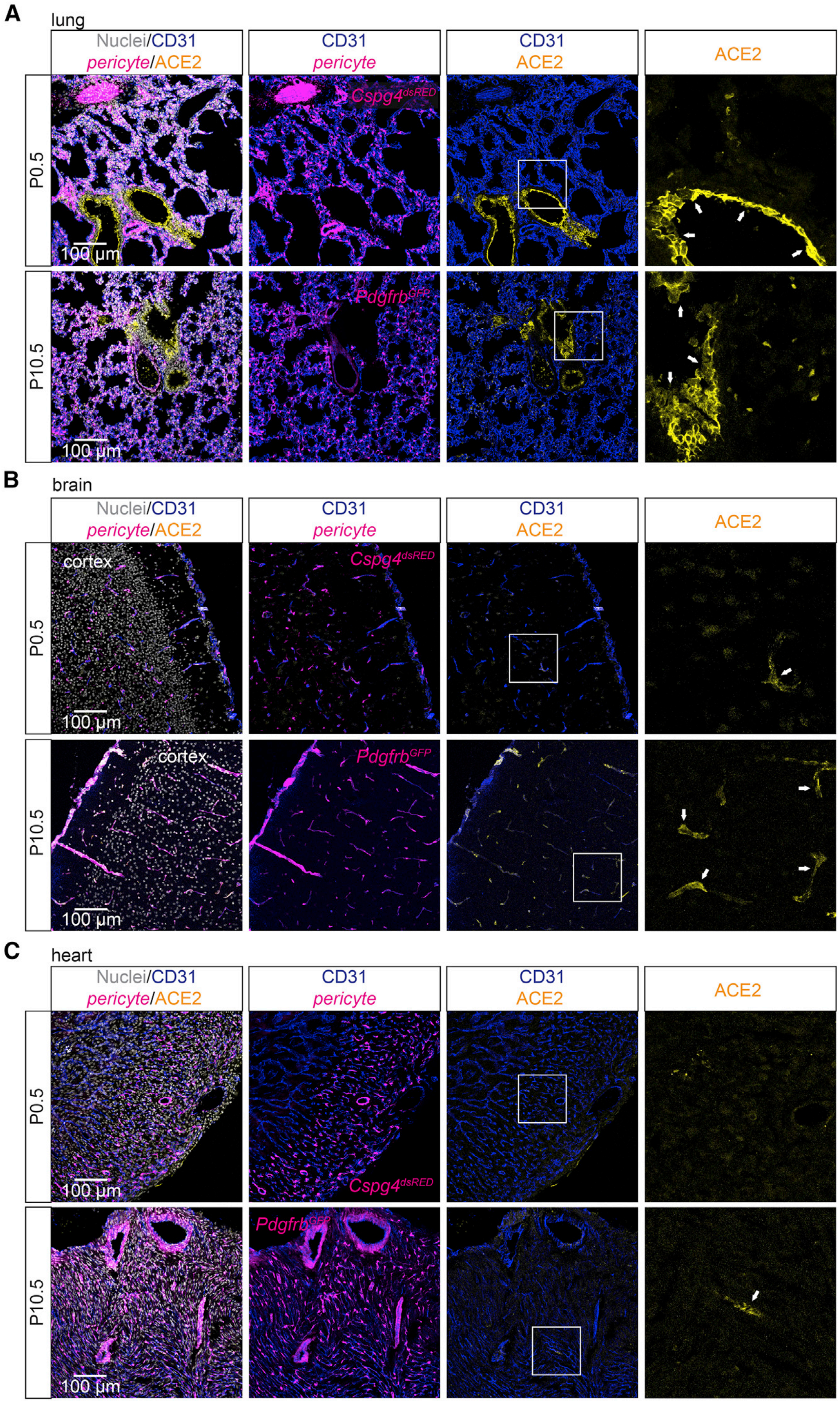

Figure 5. Developmental profile of ACE2 expression in brain, heart, and lung

(A) IF staining showing ACE2 expression in the lung at P0.5 and P10.5 in bronchial epithelial cells (arrows).

(B) IF staining showing limited ACE2 expression at P0.5 and apparent ACE2 expression at P10.5 in pericytes of the CNS (arrows).

(C) IF staining showing no expression of ACE2 at P0.5 and limited ACE2 expression at P10.5 in pericytes of the heart (arrows). Boxed areas are shown magnified in the right panel. Pericytes are indicated by either *Pdgfrb*^{GFP} or *Cspg4*^{dsRED} reporter. No staining was observed in cardiomyocytes.

Nuclei are visualized by DAPI or Hoechst 33342. Scale bars are indicated in the figure.

SARS-CoV-2 mRNA levels in wastewater (Bertels et al., 2022).

The data presented here are of relevance for COVID-19 research in mice. As standard laboratory mice cannot be infected by SARS-CoV-2, humanized ACE2 transgenic mice and mouse-adapted SARS-CoV-2 virus have been developed to circumvent the tropism problem. While some humanized mouse models only express hACE2 in the airways, they may not recapitulate the full spectrum of pathology, while mouse models where the *hACE2* gene

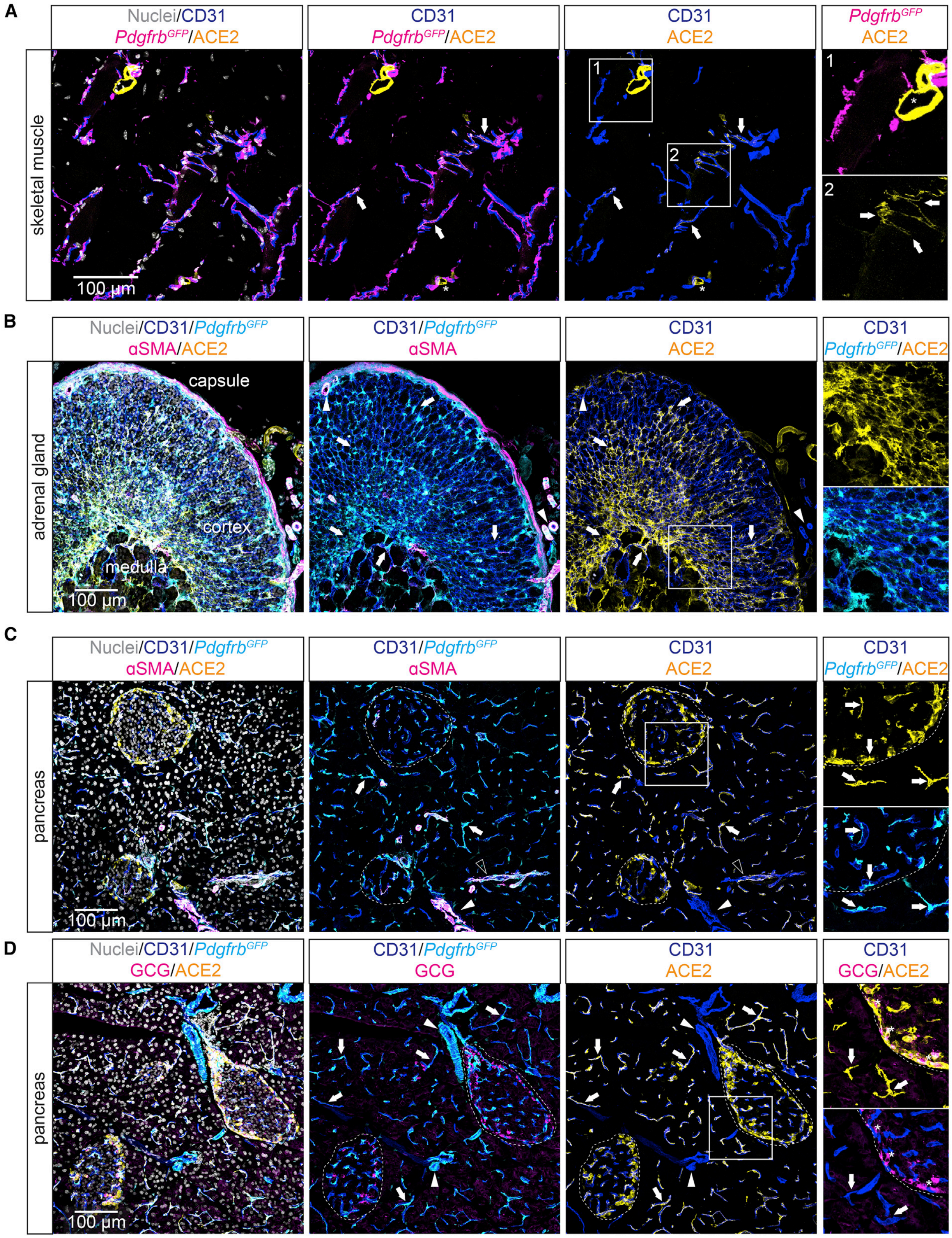

(legend on next page)

has been inserted into the mouse *Ace2* locus (Sun et al., 2020) may provide a better opportunity to assess infectability of pericytes and possible vascular consequences, as *hACE2* should be expressed like mouse *Ace2*. It will be interesting to explore in such models whether pericytes can be infected and whether infection would be facilitated by a breakdown of vascular integrity. The apparent lack of ACE2 expression in mouse cardiomyocytes, which contrasts with ACE2 being expressed in human cardiomyocytes and in cardiomyocytes derived from human pluripotent cells (Chen et al., 2020; Muus et al., 2021; Nicin et al., 2020; for review see Yiangou et al., 2021), underscores that there may be differences between human and mice regarding ACE2 expression, a notion that should be kept in mind when interpreting COVID-19 mouse experiments and translating data to the human situation.

Organoids are increasingly used in COVID-19 research (Geurts et al., 2021), and in a recent study, SARS-CoV-2 was shown to infect and multiply in human microvascular organoids composed of both endothelial and mural cells (Monteil et al., 2020), but the exact cellular tropism of the virus in this model remains to be established. Support for a pericyte-like cell as a nodal point of infection comes from analysis of neural organoids, where cortical organoids without pericyte-like cells were less efficiently infected (Wang et al., 2021). In conclusion, our data on ACE2 expression constitute a platform for further research in mouse and organoid models to better understand the vascular pathologies associated with COVID-19.

EXPERIMENTAL PROCEDURES

Single-cell RNA-seq data analysis

Mouse brain single-cell data integration

Mouse brain datasets were integrated from two internal brain single-cell projects and one published (the Tabula Muris brain resource) (Schaum et al., 2018). The internal datasets included one unpublished and one previously published brain vasculature dataset (GSE98816, GSE99058) (He et al., 2018). The cells were from 10- to 19-week-old C57Bl6 mice. The single-cell RNA-seq was conducted using the SMART-Seq2 protocol (Picelli et al., 2014) and the 10X Genomics protocol. Data processing and clustering were performed using the Seurat package (v. 3.1.1). Cells containing fewer than 200 expressed genes were filtered out. For the SMART-Seq2 data, cells that generated fewer than 50,000 reads were filtered out; for the droplet platform, cells containing fewer than 1,000 UMIs were filtered out. Furthermore, genes that were expressed by fewer than three cells in a dataset were removed. After removing low-quality cells from the dataset, the data were normalized using the LogNormalize function in the Seurat package, by which feature counts for each cell are divided by the total counts for that cell and multiplied by a scale factor (1 million) and then logarithmically transformed. For integration of different datasets, the integration workflow "Reciprocal PCA" in the Seurat package was implemented, which integrated overall datasets using the mutual nearest neighbor cell pairs that shared a common set of molecular features in their PCA spaces. We obtained a total of 12,940 cells and 17,779 genes in the integrated brain dataset.

Mouse heart single-cell data integration

scRNA-seq data were obtained from internal mouse heart single-cell projects (GSE149301) and the published Tabula Muris heart dataset (Schaum et al., 2018), collectively including diverse cell types in the heart. All samples were obtained from 6- to 20-week-old C57Bl6 mice. Data integration and clustering analysis were performed with the same methods as for the mouse brain data described above. After integration, we obtained a total of 18,378 genes and 10,101 cells for downstream analysis. The function "FindClusters" in the Seurat package was used to identify the clusters with a resolution parameter of 0.5.

Mouse lung single-cell data integration

The mouse lung datasets were obtained from internal lung single-cell projects (GSE99235) and the published Tabula Muris lung resource (Schaum et al., 2018). All samples were from 10- to 19-week-old C57Bl6 mice. Data integration and clustering analysis for the lung were performed with the same methods as for the mouse brain data described above. We obtained a total of 20,114 genes and 11,085 cells in the integrated lung dataset.

Identification of pericyte contamination of other cell types

To identify pericyte contamination in other cell types, including ECs, fibroblast-like cells, and cardiomyocytes, we examined the expression of pericyte-specific markers, including *Kcnj8*, *Pdgfrb*, and *Abcc9*. Their expression profiles in *Ace2*-positive and *Ace2*-

Figure 6. ACE2 expression in pancreas, adrenal gland, and skeletal muscle

(A) IF staining for indicated proteins and *Pdgfrb*^{GFP} reporter showing limited expression of ACE2 in pericytes of the skeletal muscle. Pericytes positive for ACE2 are indicated by arrows. The asterisk in box 1 indicates an ACE2-positive nerve.

(B) IF staining for indicated proteins and *Pdgfrb*^{GFP} reporter showing strong expression of ACE2 in cells of the medulla and cortex of the adrenal gland. Arrows highlight ACE2-positive cells (pericytes and other perivascular cells). Arrowheads indicate ACE2-negative arterial VSMCs.

(C and D) IF staining for indicated proteins showing ACE2-positive pericytes of the pancreas. Pericytes of the endocrine and exocrine pancreas that are positive for ACE2 are indicated by arrows. VSMCs of terminal arterioles are positive for ACE2 as indicated by open arrowhead (C), and ACE2-negative arteriolar/arterial VSMCs are highlighted by closed arrowhead. Islets of Langerhans (endocrine pancreas) are indicated by dashed circles. (D) ACE2-positive α-cells of the pancreas islets (marked by staining for glucagon, GCG) are indicated by asterisks (right panel). Boxed areas are shown magnified in the right panel. Nuclei are visualized by DAPI or Hoechst 33342. Scale bars are indicated in the figure.

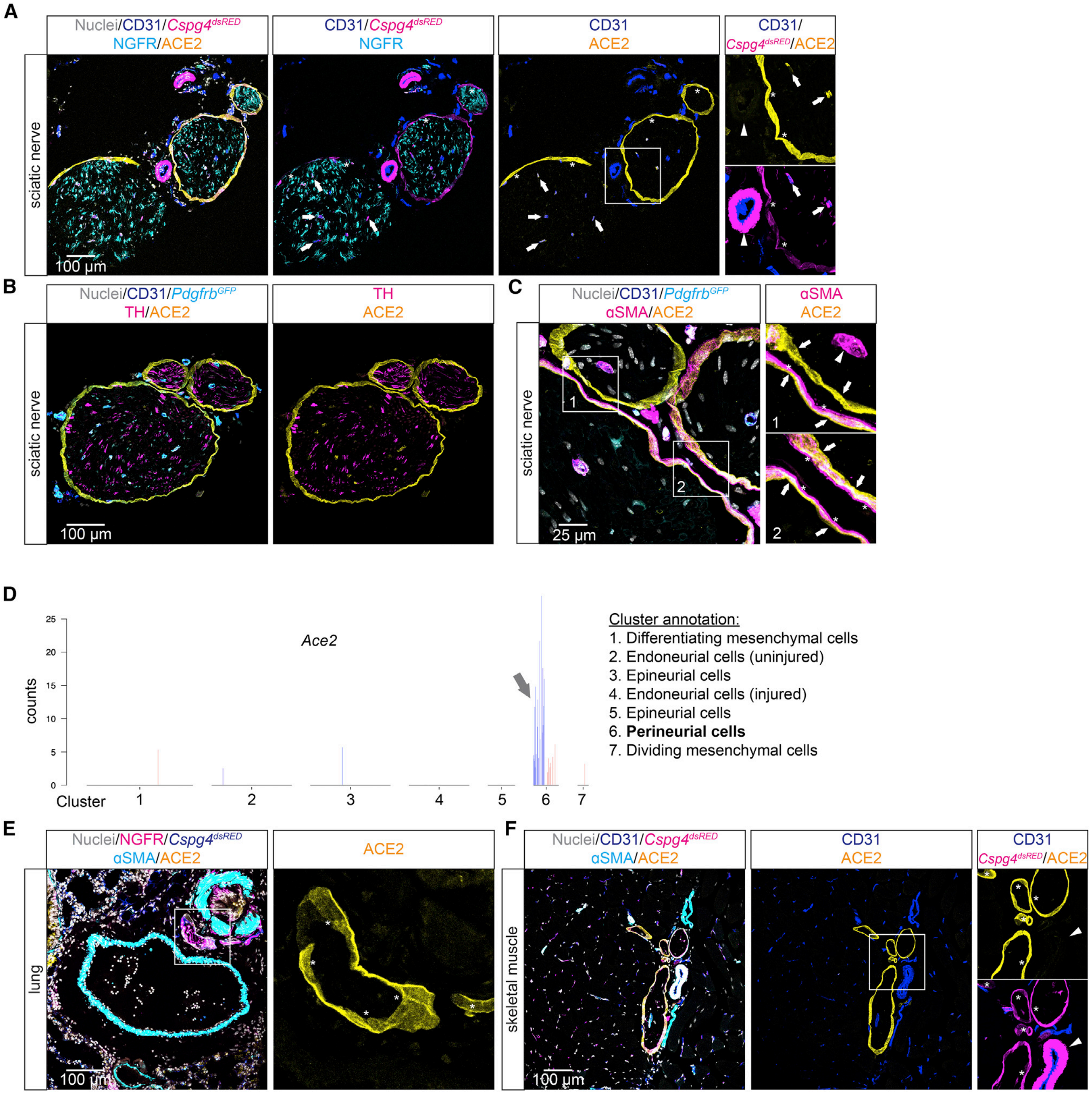

Figure 7. ACE2 expression in perineurial fibroblasts

(A–C) IF staining for indicated proteins in mouse sciatic nerve. (A) ACE2-positive pericytes of sciatic nerve at endoneurial capillaries are indicated by arrows, ACE2-positive perineurial fibroblasts are indicated by asterisks. Arteriolar/arterial VSMCs negative for ACE2 are indicated by arrowhead. (B) Overview image showing the identity of sciatic nerve by co-staining with tyrosine hydroxylase (TH). (C) High-magnification image showing the ACE2-positive perineurial fibroblast layer (arrows) in closeness, proximity to the αSMA-positive cell layer (asterisks). Arrowhead indicates ACE2-negative arteriolar/arterial VSMCs in box1.

(D) Transcriptional data from Carr et al. (2019) showing *Ace2* expression in perineurial cells (fibroblasts), visualized as bar plot. The blue and red bars indicate cells from uninjured control samples and injured samples, respectively (see original publication for cluster annotation).

(E and F) IF staining for indicated proteins in adult lung (E) and skeletal muscle (F) showing ACE2-positive perineurial fibroblast (asterisks) at peripheral nerves indicated by NGFR (nerve growth factor receptor) (E) or *Cspg4*^{dsRED} (F) staining, the latter to visualize pericytes. Arrowhead in (F) indicates ACE2-negative arteriolar/arterial VSMCs. Boxed areas are shown magnified. Nuclei are visualized by DAPI or Hoechst 33342. Scale bars are indicated in the figure.

negative cells were compared in a random selection of equal numbers of cells, and the heatmap results were visualized with pheatmap package (version 1.0.12) in R software. Also, *Ace2*-positive and *Ace2*-negative cells within the individual cell types were compared, and the top 50 enriched genes in the *Ace2*-positive cells were visualized using the DotPlot function in Seurat (version 3.1.1).

Mice

The following mouse strains were used: *Cspg4*-DsRed (The Jackson Laboratory), Tg(Cspg4-DsRed.T1)1Akik/J, *Pdgfrb*-GFP (Gensat.org, Tg(Pdgfrb-eGFP) JN169Gsat/Mmucd) (He et al., 2016), *Cldn5*-GFP (Tg(Cldn5-GFP)Cbet/U), *Acta2*^{GFP} (The Jackson Laboratory, Tg(*Acta2*-GFP)1Pfk), *Prox1-GFP* (Tg(Prox1-EGFP) KY221Gsat/Mmcd). All mice were backcrossed on a C57BL6/J genetic background. Mice from P0 to 6 months of age and of both sexes were used for experiments. Animal protocols were approved by either the Uppsala Ethical Committee on Animal Research (permit numbers C224/12, C115/15, C111515/16) or by the Stockholm/Linköping Ethical Committee on Animal Research (permit ID 729). All animal experiments were carried out in accordance with their guidelines.

Immunofluorescence staining

Mice under full anesthesia were euthanized by either transcardial perfusion with Hanks balanced salt solution (HBSS, cat. #14025092, GIBCO) followed by 4% buffered formaldehyde (cat. #02178, Histolab) or cervical dislocation. Fixation, sectioning, and antibody incubations were performed according to standard procedures, and details are described in the supplemental information.

Data and code availability

The single-cell transcriptomic data included in the paper are freely available as searchable databases at http://betsholtzlab.org/VascularSingleCells/database.html, http://betsholtzlab.org/Publications/BrainIntegration/search.html, http://betsholtzlab.org/Publications/HeartIntegration/search.html, and http://betsholtzlab.org/Publications/LungIntegration/search.html. The single-cell RNA sequencing raw data for the study can be accessed from NCBI's Gene Expression Omnibus database through the accession numbers GSE128509, GSE155387, GSE197360, GSE197529, GSE198592.

SUPPLEMENTAL INFORMATION

Supplemental information can be found online at https://doi.org/10.1016/j.stemcr.2022.03.016.

AUTHOR CONTRIBUTIONS

C.B. conceived the COVID-19-pericyte hypothesis and developed it together with T.A., X-R.P., and U.L. L.H. performed the bioinformatics analysis. L.H. and Y.S. designed the scRNA-seq meta-analysis pipeline and used it together with R.P. L.M. and M.A.M. conducted the ACE2 immunofluorescence analysis. L.M., J.L., G.G., L.Z., Y.X., S.L., G.M., S.S., A.O., M.R., A.A., J.B., M.V., K.B., E.H., K.A.H., Y.H., P.E., and T.M. provided unpublished scRNA-seq data. C.B. assembled the data. C.B. and U.L. wrote the manuscript with significant input from L.M., L.H., and M.A.M. All authors reviewed and edited the text.

CONFLICTS OF INTEREST

C.B. holds a research grant from AstraZeneca BioPharmaceuticals R&D. X-R.P is an employee of AstraZeneca BioPharmaceuticals R&D. U.L. is a member of the Editorial Board for Stem Cell Research and holds a research grant from Merck KGaA but no personal remuneration. The other authors declare no competing interests.

ACKNOWLEDGMENTS

We thank Cecilia Olsson, Pia Peterson, Sinem Karaman, Jana Chmielniakova, Helene Leksell, Sonja Gustafsson, Byambajav Buyandelger, Husain Talukdar, Elisabeth Raschperger, and the single cell core at Campus Flemingsberg (SICOF) for technical help. The computations were performed on resources provided by the Swedish National Infrastructure for Computing though Uppsala Multidisciplinary Center for Advanced Computational Science (UPPMAX) under the project SNIC 2021/22-813 and 2021/23-595. This study was supported by grants from the Swedish Research Council (C.B.: 2015-00550, U.L.: 2019-00285, T.M.: 2020-0269), the Swedish Cancer Society (C.B.:150735, T.M.: 19 0220 Pj and 19 0219 Us), the Knut and Alice Wallenberg Foundation (C.B. and T.M.: 2015.0030 and 2020.0057, T.M.: 2018.0218), the Swedish Brain Foundation (C.B. and U.L.: ALZ2019-0130 and ALZ2022-0005, U.L.: F02020-0246), the Erling-Persson Family Foundation (C.B., U.L.), the European Union, the Leducq Foundation (14CVD02), the Louis Jeantet Medical Prize, The Anders Jahre Medical Prize, ERC advanced grant, Innovative Medicines Initiative (IM2PACT-807015) (C.B.). C.B. and E.H. were supported by grants from AstraZeneca through the ICMC. L.M. was supported by the Magn. Bergvalls Foundation (2020-03735 and 2021-04275). S.S. was supported by a postdoctoral fellowship from the Deutsche Forschungsgemeinschaft (STR 1538/1-1) and a non-stipendiary long-term fellowship from the European Molecular Biology Organization (ALTF 86-2017).

Received: September 13, 2021
Revised: March 25, 2022
Accepted: March 28, 2022
Published: April 21, 2022

REFERENCES

Ackermann, M., Verleden, S.E., Kuehnel, M., Haverich, A., Welte, T., Laenger, F., Vanstapel, A., Werlein, C., Stark, H., Tzankov, A., et al. (2020). Pulmonary vascular endothelialitis, thrombosis, and angiogenesis in covid-19. N. Engl. J. Med. *383*, 120–128.

Andaloussi Mäe, M., He, L., Nordling, S., Vazquez-Liebanas, E., Nahar, K., Jung, B., Li, X., Tan, B.C., Foo, J.C., Cazenave Gassiot, A., et al. (2020). Single-cell analysis of blood-brain barrier response to pericyte loss. Circ. Res. *128*, e46–e62.

Apicella, M., Campopiano, M.C., Mantuano, M., Mazoni, L., Coppelli, A., and del Prato, S. (2020). COVID-19 in people with diabetes: understanding the reasons for worse outcomes. Lancet Diabetes Endocrinol. *8*, 782–792.

Bertels, X., Demeyer, P., van den Bogaert, S., Boogaerts, T., van Nuijs, A.L.N., Delputte, P., and Lahousse, L. (2022). Factors influencing SARS-CoV-2 RNA concentrations in wastewater up to the sampling stage: a systematic review. Sci. Total Environ. *820*, 153290.

Bocci, M., Oudenaarden, C., Sàenz-sardà, X., Simrén, J., Edén, A., Sjölund, J., Möller, C., Gisslén, M., Zetterberg, H., Englund, E., et al. (2021). Infection of brain pericytes underlying neuropathology of covid-19 patients. Int. J. Mol. Sci. *22*, 1–12.

Bunge, M.B., Wood, P.M., Tynan, L.B., Bates, M.L., and Sanes, J.R. (1989). Perineurium originates from fibroblasts: demonstration in vitro with a retroviral marker. Science *243*, 229–231.

Burkert, F.R., Lanser, L., Bellmann-Weiler, R., and Weiss, G. (2021). Coronavirus disease 2019: clinics, treatment, and prevention. Front. Microbiol. *12*, 761887.

Cantuti-Castelvetri, L., Ojha, R., Pedro, L.D., Djannatian, M., Franz, J., Kuivanen, S., van der Meer, F., Kallio, K., Kaya, T., Anastasina, M., et al. (2020). Neuropilin-1 facilitates SARS-CoV-2 cell entry and infectivity. Science *370*, 856–860.

Carr, M.J., Toma, J.S., Johnston, A.P.W., Steadman, P.E., Yuzwa, S.A., Mahmud, N., Frankland, P.W., Kaplan, D.R., and Miller, F.D. (2019). Mesenchymal precursor cells in adult nerves contribute to mammalian tissue repair and regeneration. Cell Stem Cell *24*, 240–256.e9.

Chen, L., Li, X., Chen, M., Feng, Y., and Xiong, C. (2020). The ACE2 expression in human heart indicates new potential mechanism of heart injury among patients infected with SARS-CoV-2. Cardiovasc. Res. *116*, 1097–1100.

Claesson-Welsh, L. (2015). Vascular permeability—the essentials. Upsala J. Med. Sci. *120*, 135–143.

Coate, K.C., Cha, J., Shrestha, S., Wang, W., Gonçalves, L.M., Almaça, J., Kapp, M.E., Fasolino, M., Morgan, A., Dai, C., et al. (2020). SARS-CoV-2 cell entry factors ACE2 and TMPRSS2 are expressed in the microvasculature and ducts of human pancreas but are not enriched in β cells. Cell Metab. *32*, 1028–1040.e4.

Daly, J.L., Simonetti, B., Klein, K., Chen, K.E., Williamson, M.K., Antón-Plágaro, C., Shoemark, D.K., Simón-Gracia, L., Bauer, M., Hollandi, R., et al. (2020). Neuropilin-1 is a host factor for SARS-CoV-2 infection. Science *370*, 861–865.

Deinhardt-Emmer, S., Böttcher, S., Häring, C., Giebeler, L., Henke, A., Zell, R., Jungwirth, J., Jordan, P.M., Werz, O., Hornung, F., et al. (2021). SARS-CoV-2 causes severe epithelial inflammation and barrier dysfunction. J. Virol. *95*, e00110–e00121.

Dupont, A., Rauch, A., Staessens, S., Moussa, M., Rosa, M., Corseaux, D., Jeanpierre, E., Goutay, J., Caplan, M., Varlet, P., et al. (2021). Vascular endothelial damage in the pathogenesis of organ injury in severe COVID-19. Arterioscler. Thromb. Vasc. Biol. *41*, 1760–1773.

Fignani, D., Licata, G., Brusco, N., Nigi, L., Grieco, G.E., Marselli, L., Overbergh, L., Gysemans, C., Colli, M.L., Marchetti, P., et al. (2020). SARS-CoV-2 receptor angiotensin I-converting enzyme type 2 (ACE2) is expressed in human pancreatic β-cells and in the human pancreas microvasculature. Front. Endocrinol. *11*, 1–19.

Gerber, D., Pereira, J.A., Gerber, J., Tan, G., Dimitrieva, S., Yángüez, E., and Suter, U. (2021). Transcriptional profiling of mouse peripheral nerves to the single-cell level to build a sciatic nerve atlas (Snat). Elife *10*, e58591.

Geurts, M.H., van der Vaart, J., Beumer, J., and Clevers, H. (2021). The organoid platform: promises and challenges as tools in the fight against COVID-19. Stem Cell Rep. *16*, 412–418.

Goldsmith, C.S., Miller, S.E., Martines, R.B., Bullock, H.A., and Zaki, S.R. (2020). Electron microscopy of SARS-CoV-2: a challenging task. Lancet *395*, e99.

Guo, M., Tao, W., Flavell, R.A., and Zhu, S. (2021). Potential intestinal infection and faecal–oral transmission of SARS-CoV-2. Nat. Rev. Gastroenterol. Hepatol. *18*, 269–283.

Hamming, I., Timens, W., Bulthuis, M., Lely, A., Navis, G., and van Goor, H. (2004). Tissue distribution of ACE2 protein, the functional receptor for SARS coronavirus. A first step in understanding SARS pathogenesis. J. Pathol. *203*, 631–637.

He, L., Vanlandewijck, M., Raschperger, E., Andaloussi Maë, M., Jung, B., Lebouvier, T., Ando, K., Hofmann, J., Keller, A., and Betsholtz, C. (2016). Analysis of the brain mural cell transcriptome. Sci. Rep. *6*, 1–13.

He, L., Vanlandewijck, M., Mäe, M.A., Andrae, J., Ando, K., del Gaudio, F., Nahar, K., Lebouvier, T., Lavina, B., Gouveia, L., et al. (2018). Data descriptor: single-cell RNA sequencing of mouse brain and lung vascular and vessel-associated cell types. Sci. Data *5*, 1180160.

Hoffmann, M., Kleine-Weber, H., Schroeder, S., Krüger, N., Herrler, T., Erichsen, S., Schiergens, T.S., Herrler, G., Wu, N.H., Nitsche, A., et al. (2020). SARS-CoV-2 cell entry depends on ACE2 and TMPRSS2 and is blocked by a clinically proven protease inhibitor. Cell *181*, 271–280.e8.

Jiang, R.D., Liu, M.Q., Chen, Y., Shan, C., Zhou, Y.W., Shen, X.R., Li, Q., Zhang, L., Zhu, Y., Si, H.R., et al. (2020). Pathogenesis of SARS-CoV-2 in transgenic mice expressing human angiotensin-converting enzyme 2. Cell *182*, 50–58.e8.

Lendahl, U., Nilsson, P., and Betsholtz, C. (2019). Emerging links between cerebrovascular and neurodegenerative diseases-a special role for pericytes. EMBO Rep. *20*, e48070.

Libby, P., and Lüscher, T. (2020). COVID-19 is, in the end, an endothelial disease. Eur. Heart J. *41*, 3038–3044.

Lindenmeyer, M.T., Kretzler, M., Boucherot, A., Berra, S., Yasuda, Y., Henger, A., Eichinger, F., Gaiser, S., Schmid, H., Rastaldi, M.P., et al. (2007). Interstitial vascular rarefaction and reduced VEGF-A expression in human diabetic nephropathy. J. Am. Soc. Nephrol. *18*, 1765–1776.

Lovren, F., Pan, Y., Quan, A., Teoh, H., Wang, G., Shukla, P.C., Levitt, K.S., Oudit, G.Y., Al-Omran, M., Stewart, D.J., et al. (2008). Angiotensin converting enzyme-2 confers endothelial protection and attenuates atherosclerosis. Am. J. Physiol. Heart Circ. Physiol. *295*, 1377–1384.

McCracken, I.R., Saginc, G., He, L., Huseynov, A., Daniels, A., Fletcher, S., Peghaire, C., Kalna, V., Andaloussi-Mäe, M., Muhl, L., et al. (2021). Lack of evidence of ACE2 expression and replicative infection by SARSCoV-2 in human endothelial cells. Circulation *143*, 865–868.

McCray, P.B., Pewe, L., Wohlford-Lenane, C., Hickey, M., Manzel, L., Shi, L., Netland, J., Jia, H.P., Halabi, C., Sigmund, C.D., et al. (2007). Lethal infection of K18-hACE2 mice infected with severe acute respiratory syndrome coronavirus. J. Virol. *81*, 813–821.

McGonagle, D., Bridgewood, C., Ramanan, A.v., Meaney, J.F.M., and Watad, A. (2021). COVID-19 vasculitis and novel vasculitis mimics. Lancet Rheumatol. *3*, e224–e233.

Monteil, V., Kwon, H., Prado, P., Hagelkrüys, A., Wimmer, R.A., Stahl, M., Leopoldi, A., Garreta, E., Hurtado del Pozo, C., Prosper, F., et al. (2020). Inhibition of SARS-CoV-2 infections in engineered human tissues using clinical-grade soluble human ACE2. Cell *181*, 905–913.e7.

Muhl, L., Genové, G., Leptidis, S., Liu, J., He, L., Mocci, G., Sun, Y., Gustafsson, S., Buyandelger, B., Chivukula, I.v., et al. (2020). Single-cell analysis uncovers fibroblast heterogeneity and criteria for fibroblast and mural cell identification and discrimination. Nat. Commun. *11*, 3953.

Muus, C., Luecken, M.D., Eraslan, G., Sikkema, L., Waghray, A., Heimberg, G., Kobayashi, Y., Vaishnav, E.D., Subramanian, A., Smillie, C., et al. (2021). Single-cell meta-analysis of SARS-CoV-2 entry genes across tissues and demographics. Nat. Med. *27*, 546–559.

Nicin, L., Abplanalp, W.T., Mellentin, H., Kattih, B., Tombor, L., John, D., Schmitto, J.D., Heineke, J., Emrich, F., Arsalan, M., et al. (2020). Cell type-specific expression of the putative SARS-CoV-2 receptor ACE2 in human hearts. Eur. Heart J. *41*, 1804–1806.

Nicosia, R.F., Ligresti, G., Caporarello, N., Akilesh, S., and Ribatti, D. (2021). COVID-19 vasculopathy: mounting evidence for an indirect mechanism of endothelial injury. Am. J. Pathol. *191*, 1374–1384.

Ornelas, S., Berthiaume, A.A., Bonney, S.K., Coelho-Santos, V., Underly, R.G., Kremer, A., Guérin, C.J., Lippens, S., and Shih, A.Y. (2021). Three-dimensional ultrastructure of the brain pericyte-endothelial interface. J. Cereb. Blood Flow Metab. *41*, 2185–2200.

Picelli, S., Faridani, O.R., Björklund, A.K., Winberg, G., Sagasser, S., and Sandberg, R. (2014). Full-length RNA-seq from single cells using smart-seq2. Nat. Protoc. *9*, 171–181.

Queisser, K.A., Mellema, R.A., Middleton, E.A., Portier, I., Manne, B.K., Denorme, F., Beswick, E.J., Rondina, M.T., Campbell, R.A., and Petrey, A.C. (2021). COVID-19 generates hyaluronan fragments that directly induce endothelial barrier dysfunction. JCI Insight *6*, e147472.

Rauch, A., Dupont, A., Goutay, J., Caplan, M., Staessens, S., Moussa, M., Jeanpierre, E., Corseaux, D., Lefevre, G., Lassalle, F., et al. (2020). Endotheliopathy is induced by plasma from critically ill patients and associated with organ failure in severe COVID-19. Circulation *142*, 1881–1884.

Schaum, N., Karkanias, J., Neff, N.F., May, A.P., Quake, S.R., Wyss-Coray, T., Darmanis, S., Batson, J., Botvinnik, O., Chen, M.B., et al. (2018). Single-cell transcriptomics of 20 mouse organs creates a tabula muris. Nature *562*, 367–372.

Singh, D.K., Winocour, P., and Farrington, K. (2008). Mechanisms of disease: the hypoxic tubular hypothesis of diabetic nephropathy. Nat. Clin. Pract. Nephrol. *4*, 216–226.

Sluimer, J., Gasc, J., Hamming, I., van Goor, H., Michaud, A., van den Akker, L., Jütten, B., Cleutjens, J., Bijnens, A., Corvol, P., et al. (2008). Angiotensin-converting enzyme 2 (ACE2) expression and activity in human carotid atherosclerotic lesions. J. Pathol. *215*, 273–279.

Sun, S.H., Chen, Q., Gu, H.J., Yang, G., Wang, Y.X., Huang, X.Y., Liu, S.S., Zhang, N.N., Li, X.F., Xiong, R., et al. (2020). A mouse model of SARS-CoV-2 infection and pathogenesis. Cell Host and Microbe. *28*, 124–133.e4.

Sungnak, W., Huang, N., Bécavin, C., Berg, M., Queen, R., Litvinukova, M., Talavera-López, C., Maatz, H., Reichart, D., Sampaziotis, F., et al. (2020). SARS-CoV-2 entry factors are highly expressed in nasal epithelial cells together with innate immune genes. Nat. Med. *26*, 681–687.

Teuwen, L.A., Geldhof, V., Pasut, A., and Carmeliet, P. (2020). COVID-19: the vasculature unleashed. Nat. Rev. Immunol. *20*, 389–391.

Vanlandewijck, M., He, L., Mäe, M.A., Andrae, J., Ando, K., del Gaudio, F., Nahar, K., Lebouvier, T., Laviña, B., Gouveia, L., et al. (2018). A molecular atlas of cell types and zonation in the brain vasculature. Nature *554*, 475–480.

Varga, Z., Flammer, A.J., Steiger, P., Haberecker, M., Andermatt, R., Zinkernagel, A.S., Mehra, M.R., Schuepbach, R.A., Ruschitzka, F., and Moch, H. (2020). Endothelial cell infection and endotheliitis in COVID-19. Lancet *395*, 1417–1418.

Wang, L., Sievert, D., Clark, A.E., Lee, S., Federman, H., Gastfriend, B.D., Shusta, E.v., Palecek, S.P., Carlin, A.F., and Gleeson, J.G. (2021). A human three-dimensional neural-perivascular 'assembloid' promotes astrocytic development and enables modeling of SARS-CoV-2 neuropathology. Nat. Med. *27*, 1600–1606.

Yiangou, L., Davis, R.P., and Mummery, C.L. (2021). Using cardiovascular cells from human pluripotent Stem cells for COVID-19 research: why the heart fails. Stem Cell Rep. *16*, 385–397.

Zeisel, A., Hochgerner, H., Lönnerberg, P., Johnsson, A., Memic, F., van der Zwan, J., Häring, M., Braun, E., Borm, L.E., la Manno, G., et al. (2018). Molecular architecture of the mouse nervous system. Cell *174*, 999–1014.

Ziegler, C.G.K., Allon, S.J., Nyquist, S.K., Mbano, I.M., Miao, V.N., Tzouanas, C.N., Cao, Y., Yousif, A.S., Bals, J., Hauser, B.M., et al. (2020). SARS-CoV-2 receptor ACE2 is an interferon-stimulated gene in human airway epithelial cells and is detected in specific cell subsets across tissues. Cell *181*, 1016–1035.e19.

Stem Cell Reports, Volume *17*

Supplemental Information

The SARS-CoV-2 receptor ACE2 is expressed in mouse pericytes but not endothelial cells: Implications for COVID-19 vascular research

Lars Muhl, Liqun He, Ying Sun, Maarja Andaloussi Mäe, Riikka Pietilä, Jianping Liu, Guillem Genové, Lei Zhang, Yuan Xie, Stefanos Leptidis, Giuseppe Mocci, Simon Stritt, Ahmed Osman, Andrey Anisimov, Karthik Amudhala Hemanthakumar, Markus Räsänen, Emil M. Hansson, Johan Björkegren, Michael Vanlandewijck, Klas Blomgren, Taija Mäkinen, Xiao-Rong Peng, Yizhou Hu, Patrik Ernfors, Thomas D. Arnold, Kari Alitalo, Urban Lendahl, and Christer Betsholtz

Figure S1

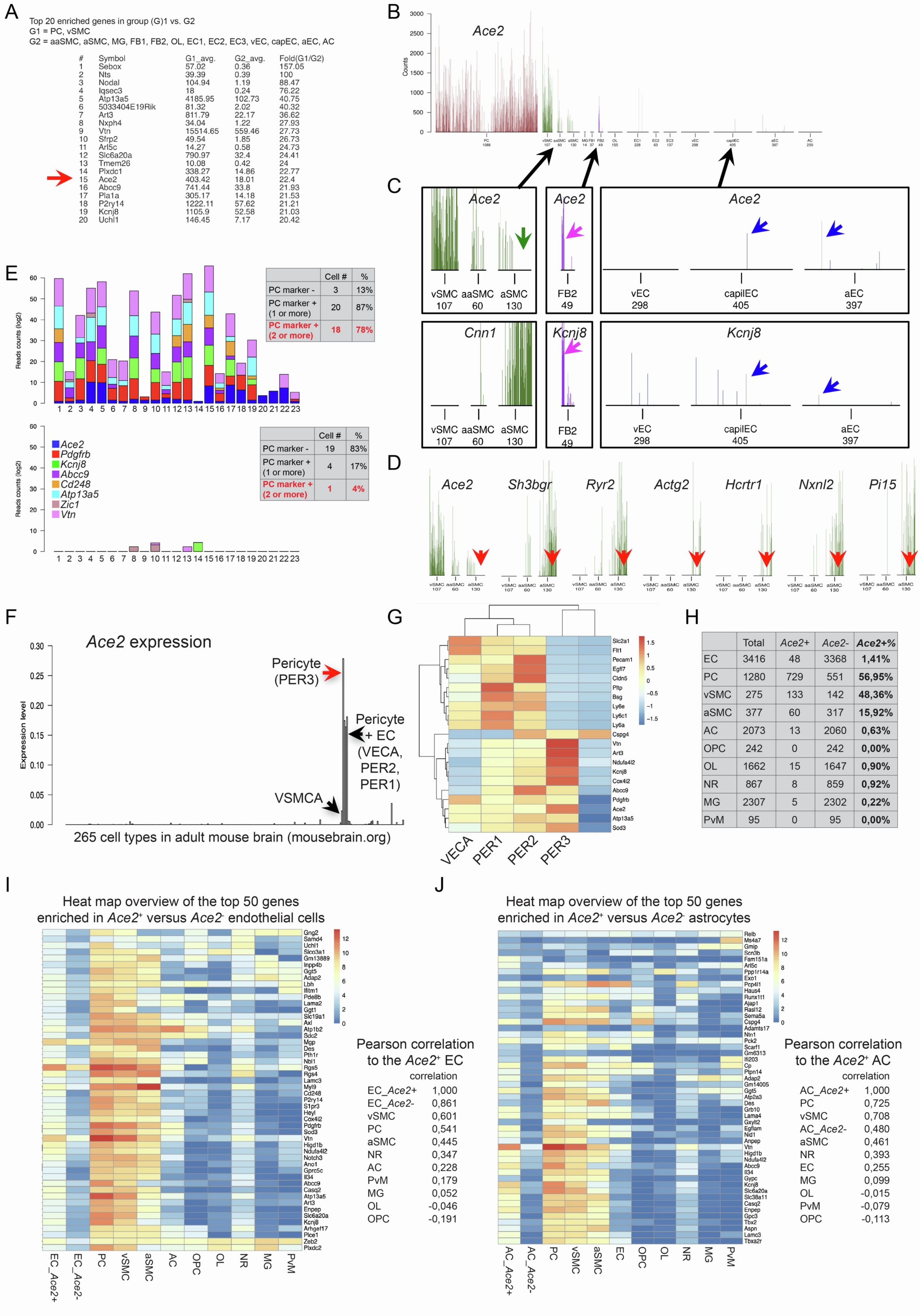

A
Top 20 enriched genes in group (G)1 vs. G2
G1 = PC, vSMC
G2 = aaSMC, aSMC, MG, FB1, FB2, OL, EC1, EC2, EC3, vEC, capEC, aEC, AC
# Symbol G1_avg. G2_avg. Fold(G1/G2)
1 Sebox 57.02 0.36 157.05
2 Nts 39.39 0.39 100
3 Nodal 104.94 1.19 88.47
4 Iqsec3 18 0.24 76.22
5 Atp13a5 4185.95 102.73 40.75
6 5033404E19Rik 81.32 2.02 40.32
7 Art3 811.79 22.17 36.62
8 Nxph4 34.04 1.22 27.93
9 Vtn 15514.65 559.46 27.73
10 Sfrp2 49.54 1.85 26.73
11 Arl5c 14.27 0.58 24.73
12 Slc6a20a 790.97 32.4 24.41
13 Tmem26 10.08 0.42 24
14 Plxdc1 338.27 14.86 22.77
15 Ace2 403.42 18.01 22.4
16 Abcc9 741.44 33.8 21.93
17 Pla1a 305.17 14.18 21.53
18 P2ry14 1222.11 57.62 21.21
19 Kcnj8 1105.9 52.58 21.03
20 Uchl1 146.45 7.17 20.42
B
Ace2
Counts
C
Ace2
vSMC 107
aaSMC 60
aSMC 130
FB2 49
vEC 298
capilEC 405
aEC 397
Cnn1
Kcnj8
D
Ace2
Sh3bgr
Ryr2
Actg2
Hcrtr1
Nxnl2
Pi15
E
Reads counts (log2)
Cell # %
PC marker - 3 13%
PC marker + (1 or more) 20 87%
PC marker + (2 or more) 18 78%
Ace2
Pdgfrb
Kcnj8
Abcc9
Cd248
Atp13a5
Zic1
Vtn
Cell # %
PC marker - 19 83%
PC marker + (1 or more) 4 17%
PC marker + (2 or more) 1 4%
F
Ace2 expression
Expression level
Pericyte (PER3)
Pericyte + EC (VECA, PER2, PER1)
VSMCA
265 cell types in adult mouse brain (mousebrain.org)
G
VECA
PER1
PER2
PER3
H
Total Ace2+ Ace2- Ace2+%
EC 3416 48 3368 1,41%
PC 1280 729 551 56,95%
vSMC 275 133 142 48,36%
aSMC 377 60 317 15,92%
AC 2073 13 2060 0,63%
OPC 242 0 242 0,00%
OL 1662 15 1647 0,90%
NR 867 8 859 0,92%
MG 2307 5 2302 0,22%
PvM 95 0 95 0,00%
I
Heat map overview of the top 50 genes enriched in Ace2+ versus Ace2- endothelial cells
Pearson correlation to the Ace2+ EC
correlation
EC_Ace2+ 1,000
EC_Ace2- 0,861
vSMC 0,601
PC 0,541
aSMC 0,445
NR 0,347
AC 0,228
PvM 0,179
MG 0,052
OL -0,046
OPC -0,191
J
Heat map overview of the top 50 genes enriched in Ace2+ versus Ace2- astrocytes
Pearson correlation to the Ace2+ AC
correlation
AC_Ace2+ 1,000
PC 0,725
vSMC 0,708
AC_Ace2- 0,480
aSMC 0,461
NR 0,393
EC 0,255
MG 0,099
OL -0,015
PvM -0,079
OPC -0,113

Figure S1. ***Ace2*** **mRNA in brain cells other than mural cells is due to pericyte contamination. Related to Figure 1.**

(A) Top enriched transcripts (red arrow indicates *Ace2* at 15th place) in pericytes and venous VSMCs as compared to other vascular and perivascular cell types deduced from the brain vascular scRNA-seq database. (B) Bar plot excerpt from http://betsholtzlab.org/VascularSingleCells/database.html showing the expression of *Ace2* in different vascular and perivascular cell types. (C) Magnified view of indicated part of A comparing the expression of *Ace2*, *Cnn1* and *Kncj8*. Abbreviations: PC, Pericytes; SMC, Smooth muscle cells; MG, Microglia; FB, Vascular fibroblast-like cells; OL, Oligodendrocytes; EC, Endothelial cells; AC, Astrocytes; v, venous; capil, capillary; a, arterial; aa, arteriolar. (D) Excerpts from http://betsholtzlab.org/VascularSingleCells/database.html showing bar plots across VSMC subtypes of arterial VSMC-specific genes that anti-correlate with *Ace2*. Red arrows point at the *Ace2*-negative part of the cluster. Cell type abbreviations: SMC, Smooth muscle cells; v, venous; a, arterial; aa, arteriolar. (E) Expression of selected known brain pericyte specific markers in 23 *Ace2*-positive ECs (top) and 23 randomly selected *Ace2*-negative ECs (bottom). Colors indicate pericyte marker as shown, and the frequency of their expression is provided in the tables. (F) *Ace2* expression in mouse brain single-cell transcriptomes from (Zeisel et al., 2018) (http://mousebrain.org/genesearch.html). Abbreviations PER - pericyte; EC - endothelial cells; VECA - vascular endothelial cells, arterial; VSMCA - vascular smooth muscle cells, arterial. (G) Heat map display of the expression of *Ace2*-positive, top 10 pericyte and top 10 endothelial markers, showing that PER1-2, VECA are pericyte contaminated. (H) Statistics of the number of cells, *Ace2*-positive cell percentage in each brain cell type. (I-J) Heat map overview of the top 50 genes enriched in *Ace2*-positive versus *Ace2*-negative ECs (I) or astrocytes (J), respectively. The *pearson* correlation was calculated based on the 50 genes.

Figure S2

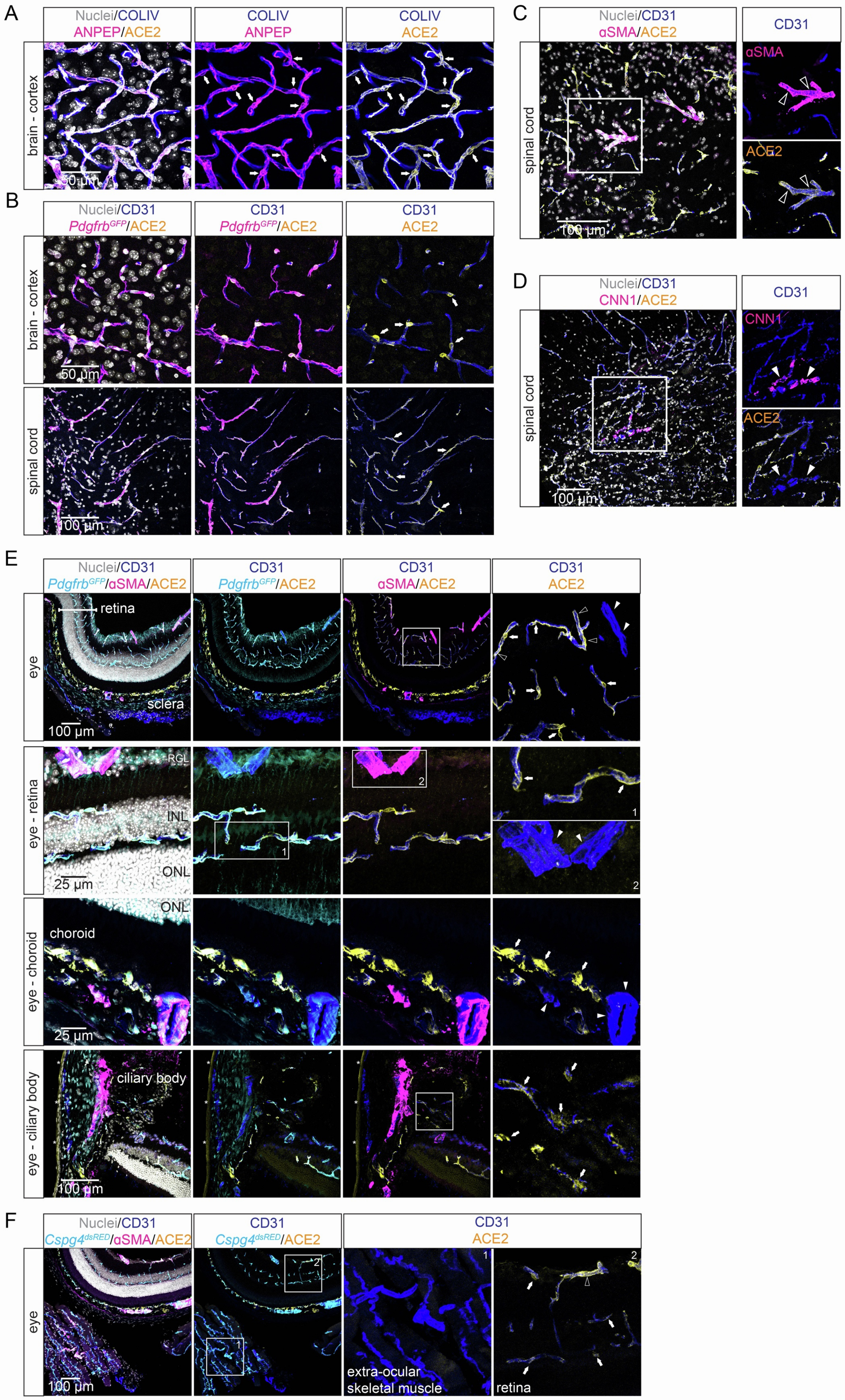

A
Nuclei/COLIV
ANPEP/ACE2
COLIV
ANPEP
COLIV
ACE2
brain - cortex
50 µm
B
Nuclei/CD31
Pdgfrb^GFP/ACE2
CD31
Pdgfrb^GFP/ACE2
CD31
ACE2
brain - cortex
50 µm
spinal cord
100 µm
C
Nuclei/CD31
αSMA/ACE2
CD31
αSMA
ACE2
spinal cord
100 µm
D
Nuclei/CD31
CNN1/ACE2
CD31
CNN1
ACE2
spinal cord
100 µm
E
Nuclei/CD31
Pdgfrb^GFP/αSMA/ACE2
CD31
Pdgfrb^GFP/ACE2
CD31
αSMA/ACE2
CD31
ACE2
eye
retina
sclera
100 µm
eye - retina
RGL
INL
ONL
25 µm
1
2
eye - choroid
ONL
choroid
25 µm
eye - ciliary body
ciliary body
100 µm
F
Nuclei/CD31
Cspg4^dsRED/αSMA/ACE2
CD31
Cspg4^dsRED/ACE2
CD31
ACE2
eye
100 µm
1
2
extra-ocular
skeletal muscle
retina

Figure S2. ACE2 protein expression in the adult mouse brain cortex, spinal cord and eye. Related to Figure 2.

(A-D) Confocal microscopy images of sections from brain cortex (A, B upper panel) and spinal cord (B lower panel, C-D) IF stained using antibodies and the *Pdgfrb*^{GFP} marker as indicated (ANPEP, aka CD13 and *Pdgfrb*^{GFP} for pericytes, αSMA and CNN1 for VSMCs). Note the strong ACE2 staining of capillary pericytes and the co-expression with ANPEP (A) and *Pdgfrb*^{GFP} (B), indicated by arrows. Mural cells in terminal arterioles are weakly ACE2-positive (C, open arrowheads), whereas mural cells of larger arterioles are ACE2-negative (arrowheads in D). (E-F) Confocal microscopy images from the eye, IF stained using antibodies and *Pdgfrb*^{GFP} or *Cspg4*^{dsRED} markers as indicated (*Pdgfrb*^{GFP} and *Cspg4*^{dsRED} for pericytes and αSMA for VSMCs). Boxed areas are shown magnified in the right panel. Note the strong expression of ACE2 in pericytes of the retina (E, upper two panels) as well as of the choriocapillaris and of the ciliary body (E, lower two panels). Similar as to the brain and spinal cord, terminal arterioles are weakly ACE2-positive (open arrowheads), while large arterioles are negative for ACE2 expression (arrowheads). Asterisks in lower panel indicate ACE2-positive cells of the surface epithelium of the conjunctiva and cornea. (F) Overview image including extra-ocular skeletal muscle. Note the absence on ACE2 expression in pericytes of the extra-ocular muscle (boxed area 1), in contrast to retinal pericytes (arrows in boxed area 2). Nuclei are visualized by DAPI or Hoechst 33342. Scale bars as indicated in the figure.

Figure S3

A

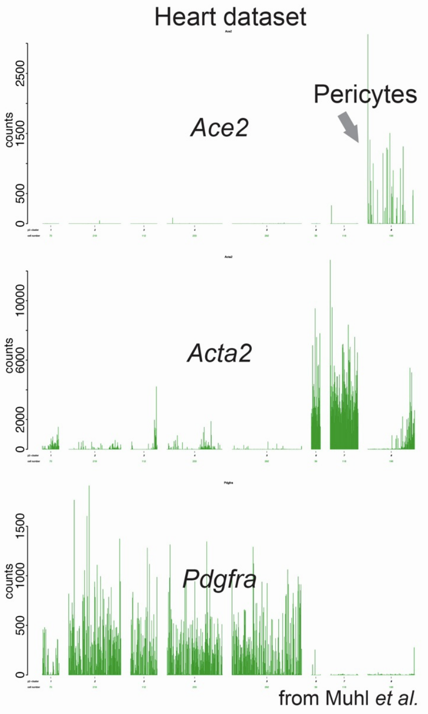

B

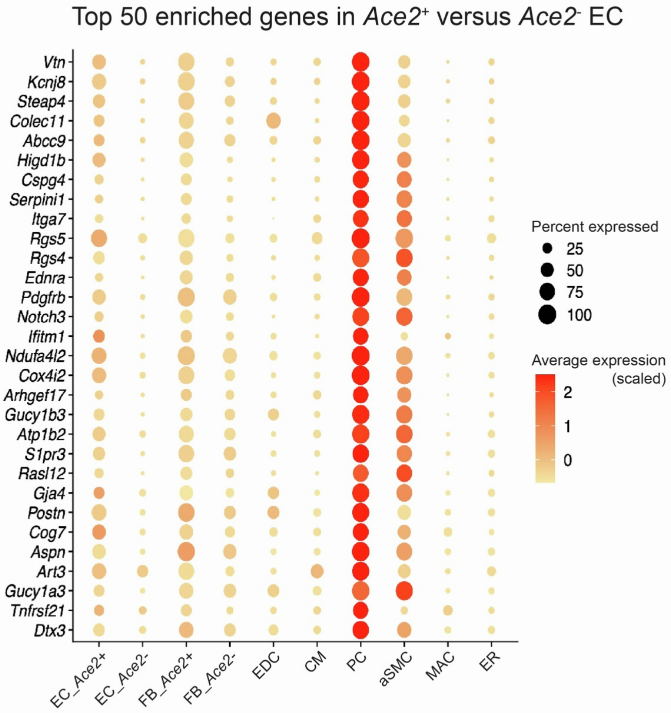

C

	Total	*Ace2+*	*Ace2-*	***Ace2+%***
EC	5017	18	4999	**0,36%**
FB	2978	63	2915	**2,12%**
EDC	208	1	207	**0,48%**
CM	307	3	304	**0,98%**
PC	369	137	232	**37,13%**
aSMC	188	6	182	**3,19%**
MAC	596	2	594	**0,34%**
ER	77	1	76	**1,30%**

D

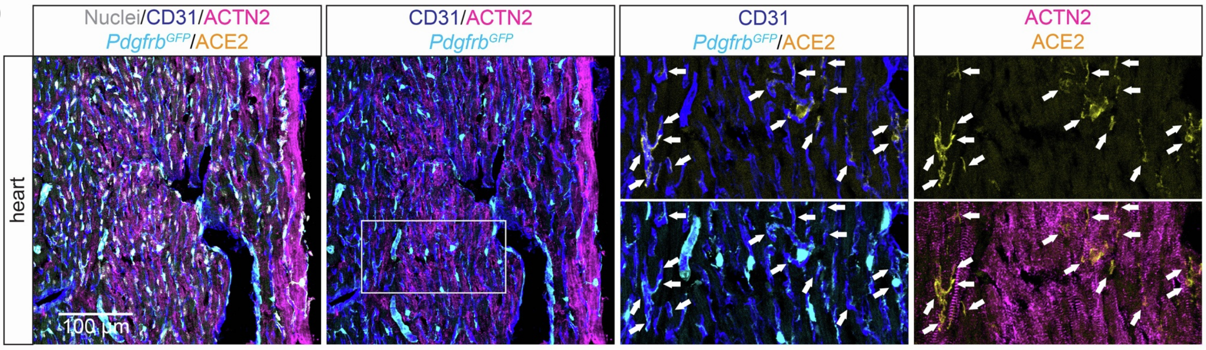

Figure S3. *Ace2* expression in mouse heart ECs is due to pericyte contamination. Related to Figure 3.

(A) *Ace2* expression in adult mouse heart scRNA-seq data enriched for stromal cells (Muhl et al., 2020). Bar plots show the expression in individual cells for *Ace2, the* VSMC marker *Acta2*, and the fibroblast maker *Pdgfra*. (B) Dot plot displaying the expression pattern of the top-50 transcripts enriched *Ace2*-positive versus *Ace2*-negative heart ECs. (C) Statistics of the number of cells, *Ace2*-positive cell percentage in each heart cell type. Abbreviations: EC, endothelial cells; FB, fibroblast; EDC, endocardial cells; CM, cardiomyocytes; PC, pericytes; aSMC, arterial smooth muscle cells; MAC, macrophages; ER, erythrocytes. (D) IF staining for ACE2 in adult mouse heart in combination with the indicated markers (*Pdgfrb*^{GFP} for pericytes, CD31 for the endothelium and sarcomeric alpha actinin (ACTN2) to mark cardiomyocytes). The arrows indicate ACE2-positive cells that are all in close contact with the vasculature and exhibit a positive *Pdgfrb*^{GFP} signal, indicating them as pericytes. In contrast, ACTN2-positive cardiomyocytes appear ACE2 negative. Nuclei are visualized by Hoechst 33342. The boxed area is shown magnified in the right panels. Scale bar is indicated in the figure.

Figure S4

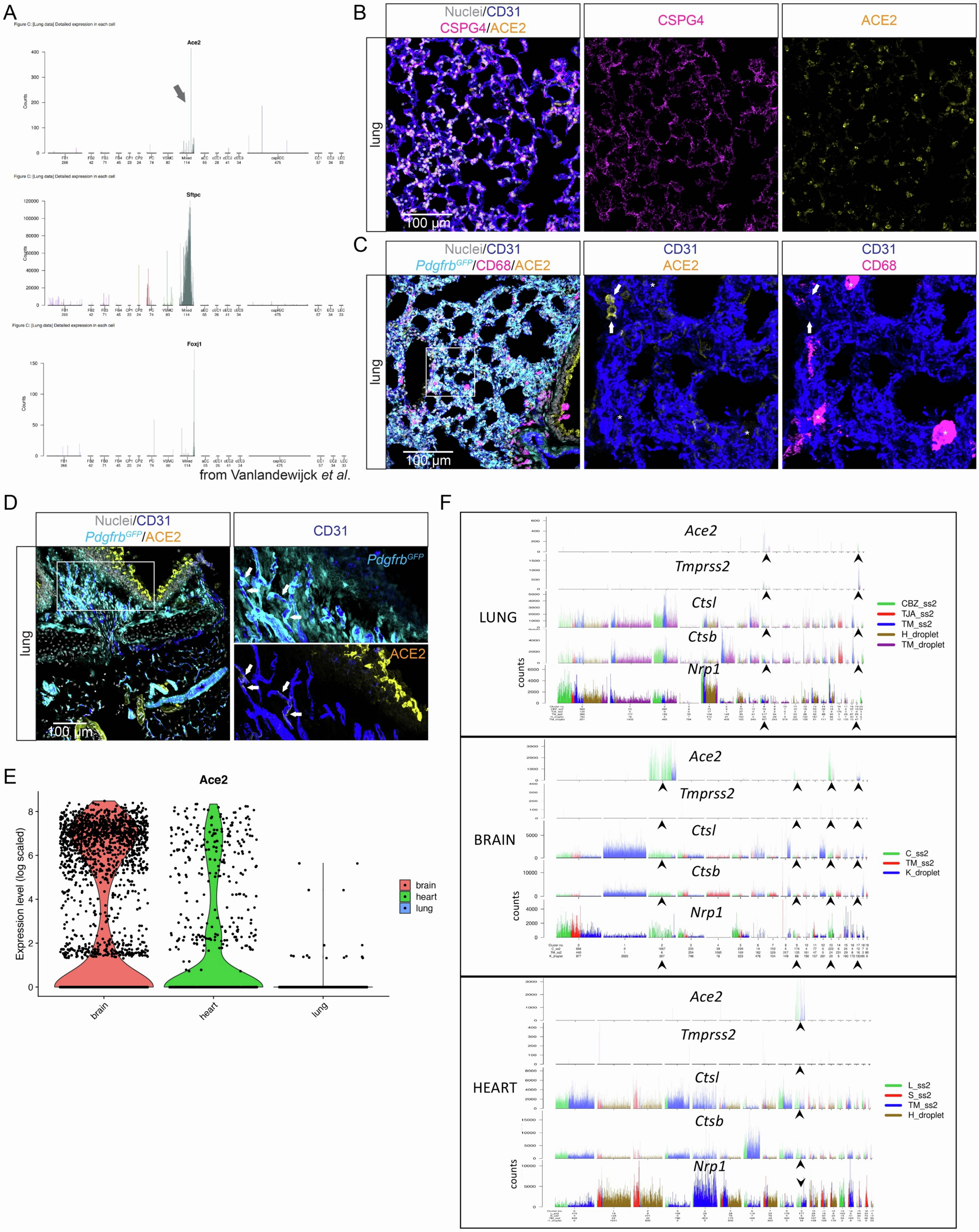
A
Ace2
Sftpc
Foxj1
from Vanlandewijck et al.
B
Nuclei/CD31
CSPG4/ACE2
CSPG4
ACE2
lung
100 µm
C
Nuclei/CD31
PdgfrbGFP/CD68/ACE2
CD31
ACE2
CD31
CD68
lung
100 µm
D
Nuclei/CD31
PdgfrbGFP/ACE2
CD31
PdgfrbGFP
ACE2
lung
100 µm
E
Ace2
Expression level (log scaled)
brain
heart
lung
F
LUNG
Ace2
Tmprss2
Ctsl
Ctsb
Nrp1
counts
CBZ_ss2
TJA_ss2
TM_ss2
H_droplet
TM_droplet
BRAIN
C_ss2
TM_ss2
K_droplet
HEART
L_ss2
S_ss2
TM_ss2
H_droplet

Figure S4. ACE2 protein expression in the adult mouse lung.

Related to Figure 4.

(A) *Ace2* expression in adult mouse lung scRNA-seq data enriched for ECs and pericytes (Vanlandewijck et al., 2018). Bar plots show the expression in individual cells for *Ace2*, the AT-II cell marker *Sftpc*, and the multiciliated cell marker *Foxj1*. (B-D) Confocal microscopy images of sections from adult mouse lung stained with the indicated antibodies (ACE2, CD31 for endothelium, CSPG4 (aka NG2) for mural cells, CD68 for macrophages) and the transgenic reporter *Pdgfrb*^{GFP} for mural cells. ACE2 IF signal is only detected in bronchial epithelium (strong) and AT-II cells (weak). Note the absence of ACE2 IF signal in ECs, in mural cells in the alveolar region and in CD68-positive macrophages (asterisk in C). (D) IF staining showing ACE2-positive pericytes (arrows) only at the capillary bed of large, primary bronchi. Nuclei are visualized by DAPI or Hoechst 33342. Scale bars as indicated in the figure. (E) Violin plot of the *Ace2* mRNA expression levels in pericytes from brain, heart and lung, as indicated. The Y-axis shows the log scaled normalized expression counts. The individual dots represent single cells, and the violin shape shows the distribution density. (F) Expression of *Ace2* and SARS-CoV-2 spike protein processing proteases – *Tmprss2*, *Ctsl* or *Ctsb* – as well as *Nrp1* across meta-analysis scRNAseq datasets from the lung (upper), brain (middle) or heart (lower) presented as bar plots. Each bar represents a single cell and is colored according to the indicated data source (see Supplementary Information - Methods). Cluster annotations have been described in previous figures. Arrows indicate *Ace2*-positive cell clusters: in the lung AT-II cells (left) and multiciliated cells (right), in the brain from left to right, pericytes, arteriolar VSMCs, venous VSMCs and pericyte-contaminated ECs, in the heart, pericytes.

Figure S5

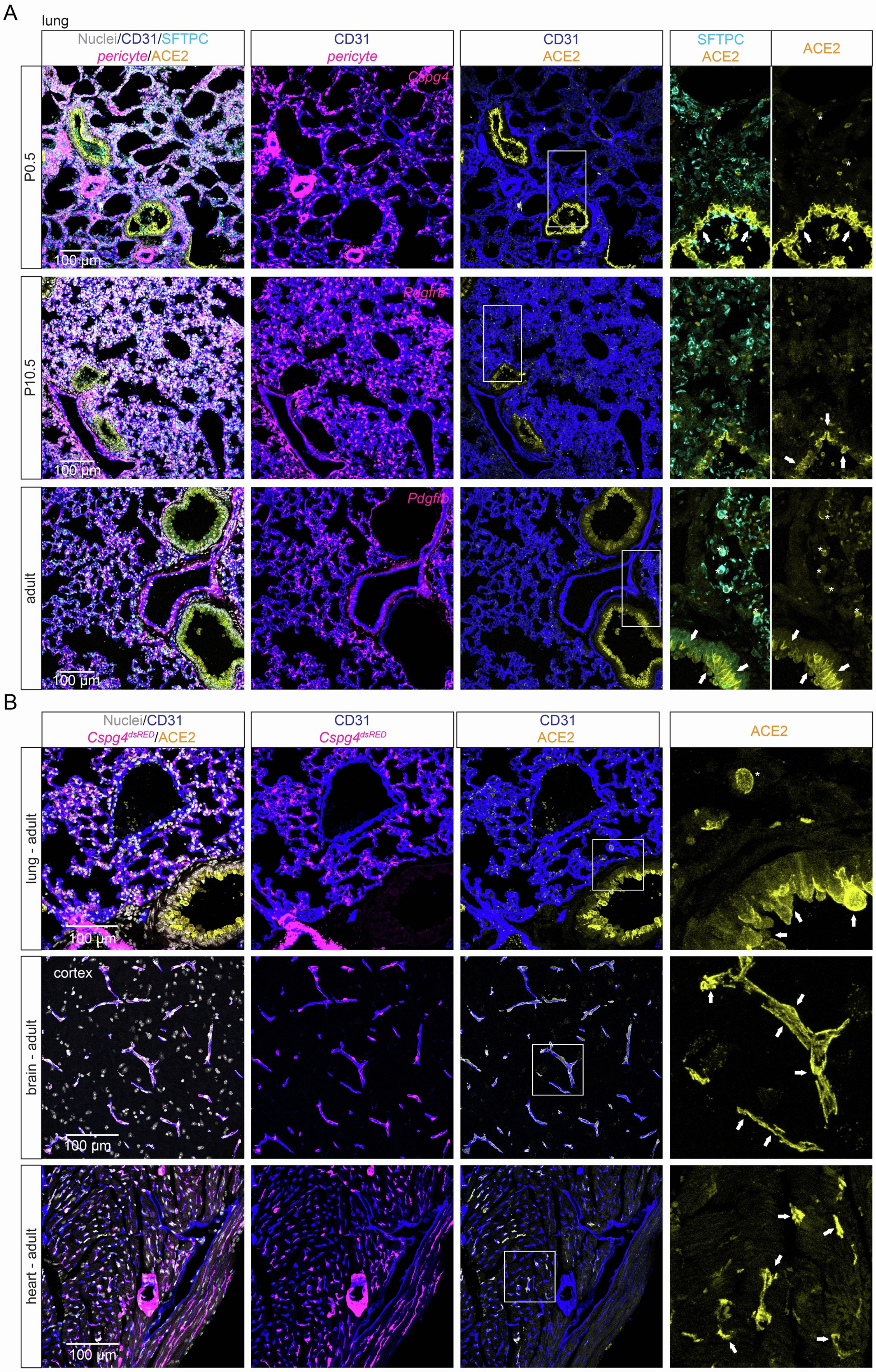
A
lung
Nuclei/CD31/SFTPC
pericyte/ACE2
CD31
pericyte
CD31
ACE2
SFTPC
ACE2
ACE2
P0.5
Cspg4
100 µm
P10.5
Pdgfrb
100 µm
adult
Pdgfrb
100 µm
B
Nuclei/CD31
Cspg4dsRED/ACE2
CD31
Cspg4dsRED
CD31
ACE2
ACE2
lung - adult
100 µm
brain - adult
cortex
100 µm
heart - adult
100 µm

Figure S5. ACE2 expression in the developing and adult lung, brain and heart. Related to Figure 5.

(A) IF staining for indicated proteins in mouse lung from P0.5, P10.5 and adult time-points. Note the sporadic expression of ACE2 in AT-II cells already visible at P0.5. Arrows indicate ACE2-positive bronchial epithelial cells, asterisks indicate ACE2-positice AT-II cells (also SFTPC-positive). Boxed areas are shown magnified in the right panel. (B) IF staining for the indicated proteins in the adult mouse lung, brain and heart. Note the strong expression of ACE2 in the bronchial epithelial cells (arrows, upper panel) and AT-II cells (asterisk, upper panel), or pericytes of the brain (middle panel) and heart (lower panel). Note the lack of ACE2 expression in cardiomyocytes in the heart. Nuclei are visualized by Hoechst 33342. Scale bars as indicated in the figure.

Figure S6

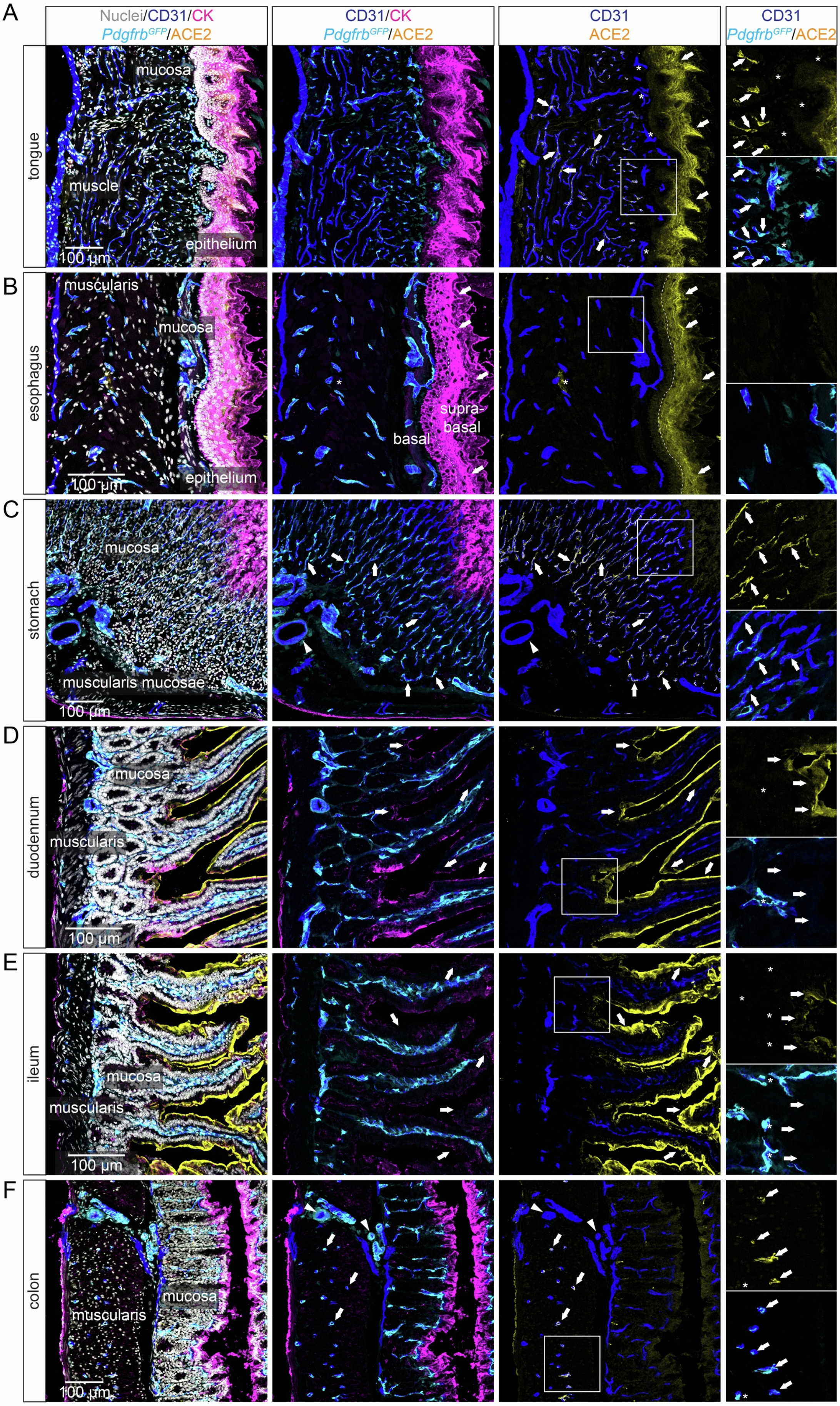

A
B
C
D
E
F
Nuclei/CD31/CK
PdgfrbGFP/ACE2
CD31/CK
PdgfrbGFP/ACE2
CD31
ACE2
CD31
PdgfrbGFP/ACE2
tongue
esophagus
stomach
duodenum
ileum
colon
mucosa
muscle
epithelium
100 µm
muscularis
basal
supra-
basal
muscularis mucosae

Figure S6. ACE2 protein expression in the adult gastrointestinal tract.

Related to Figure 7.

IF detection of ACE2 in adult mouse gastrointestinal (GI) tract in combination with the indicated markers (*Pdgfrb*^{GFP} for pericytes, CD31 for endothelial cells and cytokeratin (CK) is used for epithelial structures). (A) IF staining of adult mouse tongue, showing strong ACE2 expression, highlighted by arrows, in the surface epithelium as well as pericytes of the tongue muscle. Asterisks indicate ACE2-negative pericytes of the mucosal vasculature. (B) IF staining of adult mouse esophagus, showing strong ACE2 expression in the surface epithelium, highlighted by the arrows. The basal cell layer exhibits a weaker ACE2 signal, compared to the suprabasal cell layer. The border between basal and suprabasal cell layers is indicated by the dashed line. The asterisk indicates an ACE2-positive nerve. (C) IF staining of adult mouse stomach, showing ACE2 expression in pericytes of the mucosal vasculature, highlighted by arrows. (D) IF staining of adult mouse duodenum. The arrows highlight the ACE2-positive surface epithelium of the villi and crypts. The asterisk indicates ACE2 negative pericytes of the mucosal vasculature. (E) IF staining of adult mouse ileum. The arrows highlight the ACE2-positive surface epithelium of the villi. In the magnified box, arrows indicate the, compared to the duodenum, low signal of ACE2 at the crypt of the ileum and asterisks indicate ACE2-negative pericytes of the mucosa vasculature. (F) IF staining of adult mouse colon. The arrows indicate ACE2-positive pericytes of the muscularis vasculature. Not all pericytes of the muscularis vascular system are positive and an ACE2-negative pericyte is marked by the asterisk. Arrowheads highlight ACE2-negative arterial VSMC. Boxed areas are shown magnified in the right panel. Nuclei are visualized by Hoechst 33342. Scale bars are indicated in the figure.

Figure S7

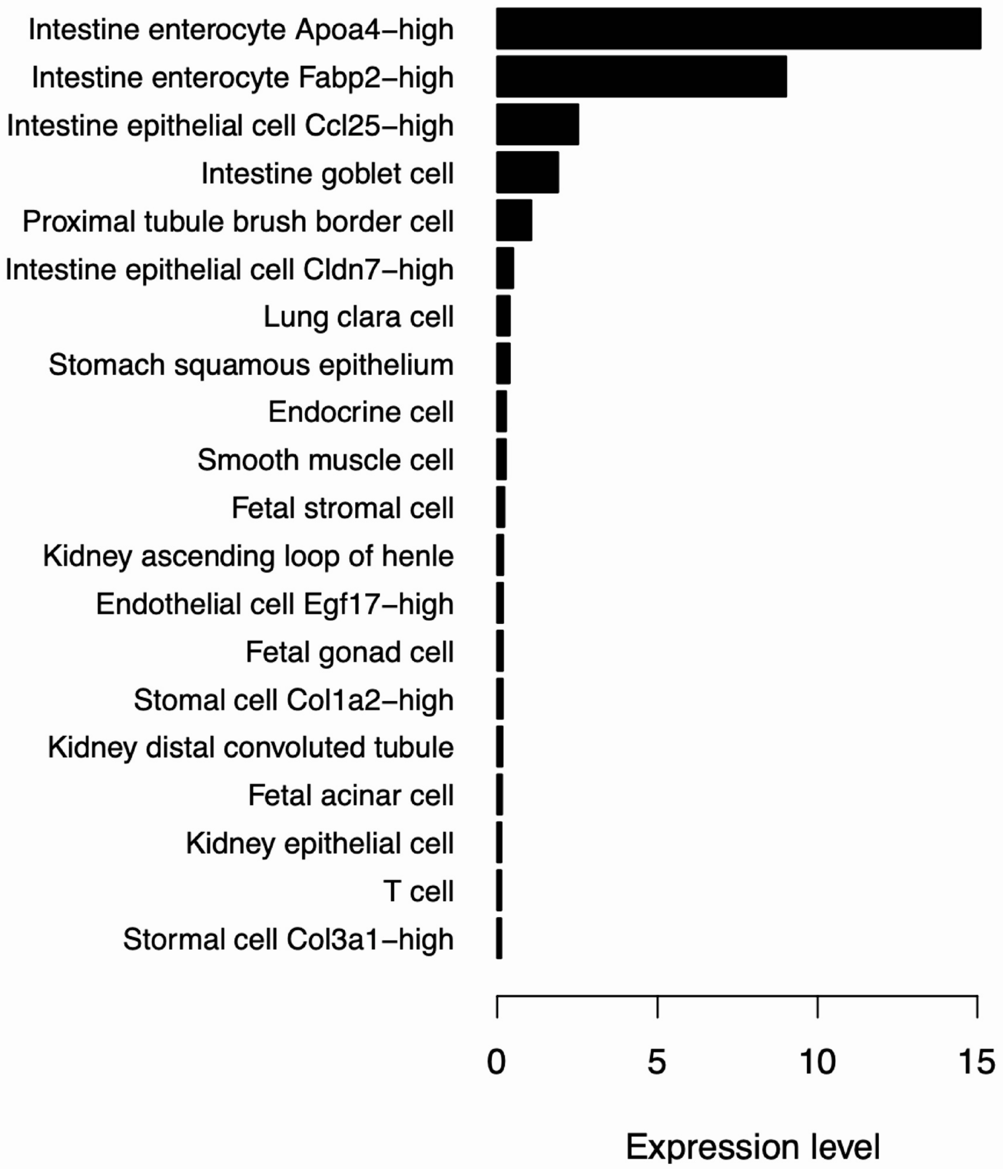

Figure S7. *Ace2* expression in the adult mouse gastrointestinal tract. Related to Figure 7.

Bar plot of the *Ace2* mRNA expression levels in main mouse cell types (data obtained from the Mouse Cell Atlas (http://bis.zju.edu.cn/MCA/index.html). The X-axis shows the average normalized expression level.

Table S1. List of used antibodies.

Primary antibody	Dilutions	Supplier	Catalog number
PECAM1, CD31	1:200	R & D Systems	AF3628
PECAM1, CD31	1:100	BD Pharmingen	553370
PECAM1, CD31	1:50	Abcam	ab28364
ANPEP	1:100	Bio-Rad	MCA2183EL
COLIV	1:100	Bio-Rad	2150-1470
ACTA2 (aSMA)-Alexa Fluor 647	1:200	Santa Cruz Biotechnology, Inc.	sc-32251
ACTA2 (aSMA)-FITC	1:200	Sigma	F3777
ACTA2 (aSMA)-Cy3	1:500	Sigma	C6198
PDGFRb	1:100	eBiosciences	553847
ACE2	1:100	R & D Systems	AF3437
SFTPC	1:100	Abcam	ab40879
CSPG4 (NG2)	1:200	Millipore	AB5320
CD68	1:200	BioLegend	137001
GLUCAGON (GCG)	1:200	Millipore	AB932
NGFR	1:200	Abcam	ab52987
THYROSIN HYDROXYLASE (TH)	1:200	Pel Freez Biologicals	P40101-0
SARCOMERIC ALPHA-ACTININ (ACTN2)	1:300	Abcam	ab137346
CYTOKERATIN (CK)	1:200	DAKO	Z0622

Supplemental Experimental Procedures:

Fixation, sectioning and antibody incubations:

Vibratome-sections: Brains were removed and post-fixed in 4% buffered formaldehyde for 4h at 4°C. Sagittal and coronal vibratome sections (50-75 µm) were incubated in blocking/permeabilization solution (1% bovine serum albumin, 2.5% donkey serum, 0.5% Triton X-100 in PBS) overnight at 4°C, followed by incubation in primary antibody solution for two nights at 4°C, and subsequently in secondary antibody (Jackson ImmunoResearch and Invitrogen) solution, overnight at 4°C. A list of the used primary antibodies is presented in Table S1. Sections were mounted in ProLong Gold Antifade mounting medium (cat. #P36930, Life Technologies). Micrographs were acquired with a Leica TCS SP8 confocal microscope (Leica Microsystems). All confocal images are represented as maximum intensity projections and were adjusted for brightness and contrast using Fiji v1.52p and Adobe Photoshop CC 2019.

Cryo-sections: Tissues were harvested from euthanized mice without perfusion and fixed by immersion in 4% formaldehyde for 4-12h at 4°C, followed by immersion in 20% sucrose/PBS solution for at least 24h at 4°C. Thereafter, tissues were embedded for cryo-sectioning and sectioned on a CryoStat NX70 (ThermoFisher Scientific) to 14 or 30 µm thick sections collected on SuperFrost Plus glass slides (Metzler Gläser) and stored at -80°C until usage. Sections were allowed to thaw at RT and thereafter blocked for > 60 min at RT with blocking-buffer (serum-free protein blocking solution, DAKO), supplemented with 0.2% Triton X-100 (Sigma Aldrich), followed by sequential incubation with primary antibodies (overnight at 4°C) (Table S1) and corresponding fluorescently conjugated secondary antibodies (1h at RT) together with 10 µg/ml *Hoechst 33342* (trihydrochloride, trihydrate, ThermoFisher Scientific). Sections were mounted with ProLong Gold Antifade mounting medium, and micrographs acquired and graphically handled as described above.
